# A chromosome-anchored eggplant genome sequence reveals key events in Solanaceae evolution

**DOI:** 10.1038/s41598-019-47985-w

**Published:** 2019-08-13

**Authors:** Lorenzo Barchi, Marco Pietrella, Luca Venturini, Andrea Minio, Laura Toppino, Alberto Acquadro, Giuseppe Andolfo, Giuseppe Aprea, Carla Avanzato, Laura Bassolino, Cinzia Comino, Alessandra Dal Molin, Alberto Ferrarini, Louise Chappell Maor, Ezio Portis, Sebastian Reyes-Chin-Wo, Riccardo Rinaldi, Tea Sala, Davide Scaglione, Prashant Sonawane, Paola Tononi, Efrat Almekias-Siegl, Elisa Zago, Maria Raffaella Ercolano, Asaph Aharoni, Massimo Delledonne, Giovanni Giuliano, Sergio Lanteri, Giuseppe Leonardo Rotino

**Affiliations:** 1 University of Torino - DISAFA - Plant Genetics and Breeding, Largo Braccini 2, 10095 Grugliasco, Torino Italy; 2 Italian National Agency for New Technologies, Energy and Sustainable Development (ENEA), Casaccia Res Ctr, Via Anguillarese 301, 00123 Roma, Italy; 3 Council for Agricultural Research and Economics (CREA), Research Centre for Olive, Citrus and Tree Fruit, 47121 Forlì, Italy; 4 Council for Agricultural Research and Economics (CREA), Research Centre for Genomics and Bioinformatics, 26836 Montanaso Lombardo, LO Italy; 5 Department of Biotechnology, University of Verona, Strada Le Grazie 15, 37134 Verona, Italy; 6 Department of Life Sciences, Natural History Museum, Cromwell Rd, Kensington, London United Kingdom; 7 Department of Agricultural Sciences, University of Naples Federico II, 80055 Portici, Italy; 8 Department of Plant and Environmental Sciences, Weizmann Institute of Science, Rehovot, 7610001 Israel; 9 UC Davis Genome Center-GBSF, 451 Health Sciences Drive, University of California, Davis, CA 95616 USA; 10 IGA Technology Services, Via J. Linussio, 51, 33100 Udine, Italy

**Keywords:** Genomics, Genomics, Sequencing, Sequencing

## Abstract

With approximately 450 species, spiny *Solanum* species constitute the largest monophyletic group in the Solanaceae family, but a high-quality genome assembly from this group is presently missing. We obtained a chromosome-anchored genome assembly of eggplant (*Solanum melongena*), containing 34,916 genes, confirming that the diploid gene number in the Solanaceae is around 35,000. Comparative genomic studies with tomato (*S*. *lycopersicum*), potato (*S*. *tuberosum*) and pepper (*Capsicum annuum*) highlighted the rapid evolution of miRNA:mRNA regulatory pairs and R-type defense genes in the Solanaceae, and provided a genomic basis for the lack of steroidal glycoalkaloid compounds in the *Capsicum* genus. Using parsimony methods, we reconstructed the putative chromosomal complements of the key founders of the main Solanaceae clades and the rearrangements that led to the karyotypes of extant species and their ancestors. From 10% to 15% of the genes present in the four genomes were syntenic paralogs (ohnologs) generated by the pre-γ, γ and T paleopolyploidy events, and were enriched in transcription factors. Our data suggest that the basic gene network controlling fruit ripening is conserved in different Solanaceae clades, and that climacteric fruit ripening involves a differential regulation of relatively few components of this network, including *CNR* and ethylene biosynthetic genes.

## Introduction

The Solanaceae family comprises around 2,700 plant species, adapted to vastly different environments, and grown for food (tomato, potato, eggplant, pepper), medicinal or recreational uses (belladonna, corkwood tree, mandrake, tobacco) as well as ornamentals (petunia and *Brunfelsia*). The “giant” genus *Solanum* includes around 1,500 species, among which three staple crops: tomato (*S*. *lycopersicum*), potato (*S*. *tuberosum*) and eggplant (*S*. *melongena*). Unlike tomato and potato, eggplant is native to the Old World, evolved from *S*. *insanum* and was independently domesticated in the Indian subcontinent and in China^1–3^. Eggplant is a representative of the subgenus *Leptostemonum* (spiny *Solanum* species), which with around 450 species is the largest monophyletic group in the whole family^4^. Unlike tomato and similar to pepper, eggplant fruits display ethylene-independent ripening. Several Solanaceae chromosome-anchored genome sequences have been generated to this date, including the ones of potato, tomato and pepper^5–8^, but a chromosome-anchored reference genome from the *Leptostemonum* subgenus is presently lacking. A highly fragmented, non chromosome-anchored draft sequence of eggplant is available^9^ with an N50 of 64 Kb and 85,446 predicted genes, a number much larger than the approximately 35,000 genes annotated in the other sequenced diploid Solanaceae genomes (Table 1). In this paper, we describe a reference, chromosome-anchored genome sequence of eggplant, and its use for comparative genomics studies with tomato, potato and pepper.

**Table 1:** Assembly and annotation metrics of the eggplant genome and its comparison with other high-quality Solanaceae genomes and the previous eggplant draft.

	Eggplant (this work)	Eggplant Kazusa	Potato (ITAG1.0)	Tomato (ITAG2.4)	Pepper (PGA v1.55)
Projected size (Gb)	1.21	1.13	0.84	0.90	3.30
Number of scaffolds	10,383	33,873	66,254	3,223	37,989
Ungapped length of scaffolds (Gb)	1.06 (88%)	0.78 (69%)	0.58 (69%)	0.74 (82%)	2.96 (90%)
Ungapped length of anchored Scaffolds (Gb)	0.89 (73%)	—	0.58 (69%)	0.72 (80%)	2.67 (81%)
N50 of anchored scaffolds (Mb)	2.9	0.065	1.3	16.5	2.4
Protein coding genes	34,916	85,446	35,004	34,725	34,899
Of which organellar (unique)	2,058	—	1,734	1,706	2,594
BUSCO genes present in the annotation	1,332 (96.9%)	1,028 (74.8%)	1,334 (97.0%)	1,370 (99.6%)	1,124 (81.8%)
Anchored genes	28,435	—	35,004	33,838	30,242
Total length of repeats (Mb)	772 (73%)	—	499 (59%)	426 (47%)	1.98 (60%)

## Results

### Genome assembly, anchoring and annotation

We sequenced and assembled the genome of the inbred eggplant line ‘67/3’, the male parent of a F6 Recombinant Inbred Line (RIL) population, using a combination of Illumina sequencing and single molecule optical mapping (Supplementary Information 1.1–1.5.3). A 1.16 Gb draft, with an N50 of 0.68 Mb, was obtained from Illumina paired end and mate pair libraries assembled using Soapdenovo2^10^, while the optical map^11^ covered 1.18 Gb, with an N50 of 2.56 Mb. Their hybrid assembly included 0.92 Gb (ungapped) and 1.22 Gb (gapped) sequence in 469 scaffolds with an N50 of 3.59 Mb (Table 1, Supplementary Tables S2 and S3). We estimated the eggplant haploid genome size at 1.21 Gb (by flow cytometry) and 1.04 Gb (by k-mer distribution) (Supplementary Fig. S1). The latter is probably an underestimate of the real genome size, as suggested by the presence of secondary peaks in the distribution due to repeats (Supplementary Fig. S1).

**Figure 1:**
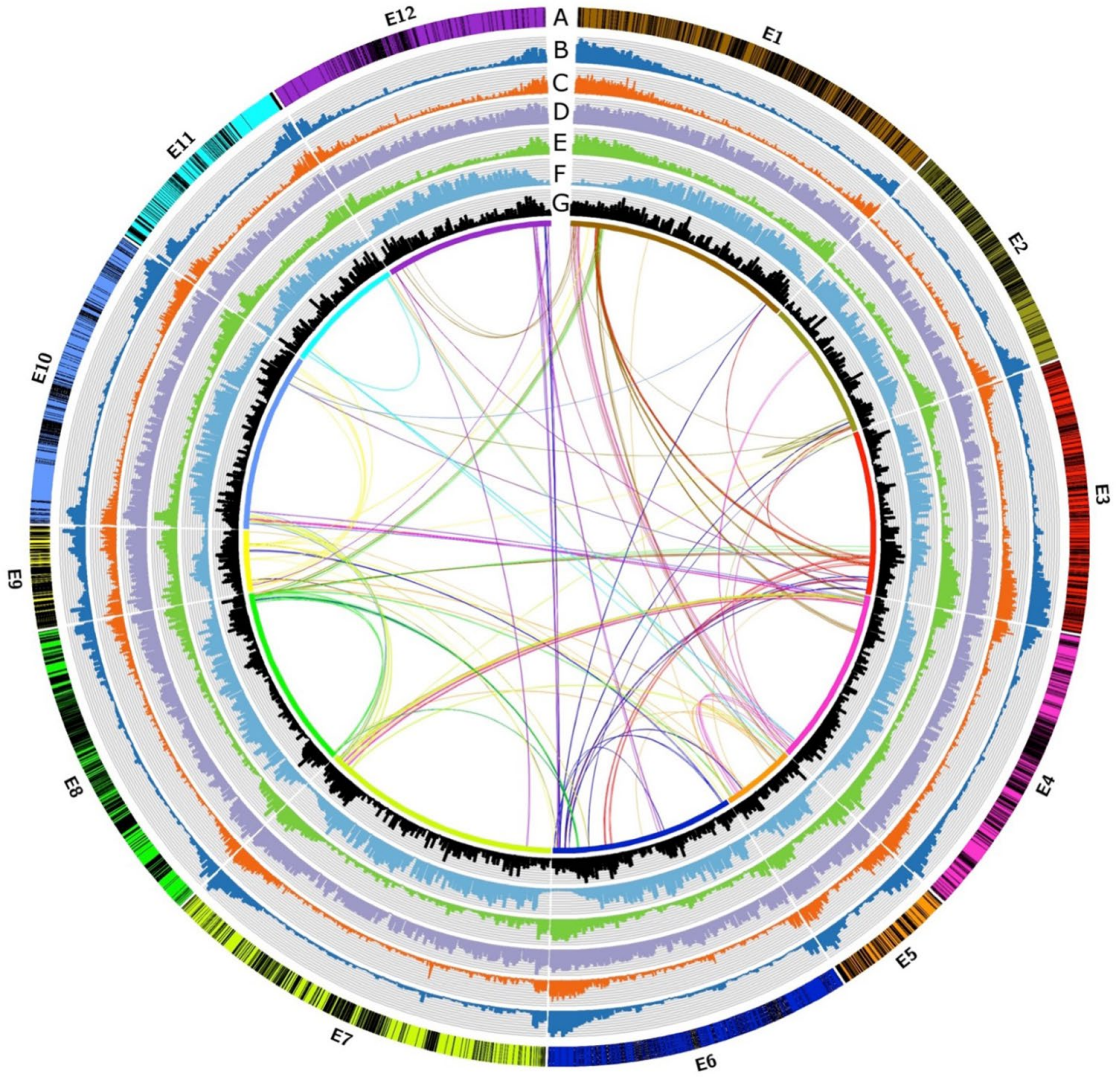
Topography of the eggplant genome. Track (**A**) Eggplant chromosomes with the genetic map (black bars represent the position of genetic markers used for anchoring scaffolds to pseudomolecules; Track (**B**) gene density; Track (**C**) RNA-seq expression data density; Track (**D**) overall repeat density; Track (**E**) DNA transposon density; Track (**F**) LTR-Gypsy transposon density; Track (**G**) LTR-Copia transposon density; Track (**H**) Intragenome syntenic regions originated from paleopolyploid events (for the parameters used to define syntenic thresholds, see Supplementary Information 2.7). Densities for tracks (**B**,**D**,**E**,**F**,**G**) are presented in 1-Mb intervals; for track (**C**) in 100-Kb intervals).

Using the SoiLoCo pipeline^12^ and a linkage map comprising 5,964 markers, developed from an F6 Recombinant Inbred Line (RIL) mapping population^13^, we anchored the hybrid scaffolds to chromosomes (Supplementary Information 1.5.4–15.5, Fig. 1). Anchored pseudomolecules comprised 1.14 Gb (gapped) and 0.82 Gb (ungapped) genome sequences. The quality of the new assembly is comparable to the ones of tomato, potato and pepper, and significantly improved the metrics of the previous eggplant draft (Table 1). A sequence assembly of line ‘305E40’, the female parent of the mapping population, was also obtained, with a total size of 1.09 Gb and an N50 of 6.9 Kb. Residual heterozygosity was estimated at 0.027% for ‘67/3’ and 0.067% for ‘305E40’ (Supplementary Information 1.6).

We annotated the ‘67/3’ assembly with the Maker pipeline^14^ and RNA-Seq data from 19 tissues of ‘67/3’, obtaining 34,916 high-quality protein-coding gene models (Supplementary Information 2.3). This number is comparable to the one of other sequenced Solanaceae genomes and lower than the one previously reported^9^ (Table 1). Quality controls, based on several pipelines, confirmed that the annotation quality is comparable to that of tomato, potato and pepper (Table 1, Supplementary Information 2.3.3). In particular, the annotation comprised 96.9% of Benchmarking Universal Single-Copy Orthologs (BUSCO)^15^ (Table 1, Supplementary Fig. S3). About 97% of the annotated ‘67/3’ CDSs were found also in the ‘305E40’ assembly. The genomic landscape of the 12 eggplant chromosomes, similarly to other Solanaceae, shows gene-rich distal chromosome arms and gene-poor peri-centromeric heterochromatin (Fig. 1). Based on whole genome data, we estimate the divergence of eggplant from tomato/potato at 15 mya, and from pepper at 20 mya (Supplementary Information 2.7). OrthoMCL^16^ analysis (Supplementary Information 2.4) showed that 667 gene families are exclusively found in the eggplant lineage with respect to other eudicot lineages (tomato, potato, pepper and Arabidopsis, Supplementary Fig. S4). The most common annotation in these families is “pentatricopeptide repeat-containing protein” a family of proteins binding to organellar transcripts and modulating organellar gene expression^17^ (Supplementary Table S18). The eggplant genome contains >800 genes encoding pentatricopeptide repeat proteins, about twice the number found in the other four genomes.

### Solanaceae genome dynamics

**Figure 2:**
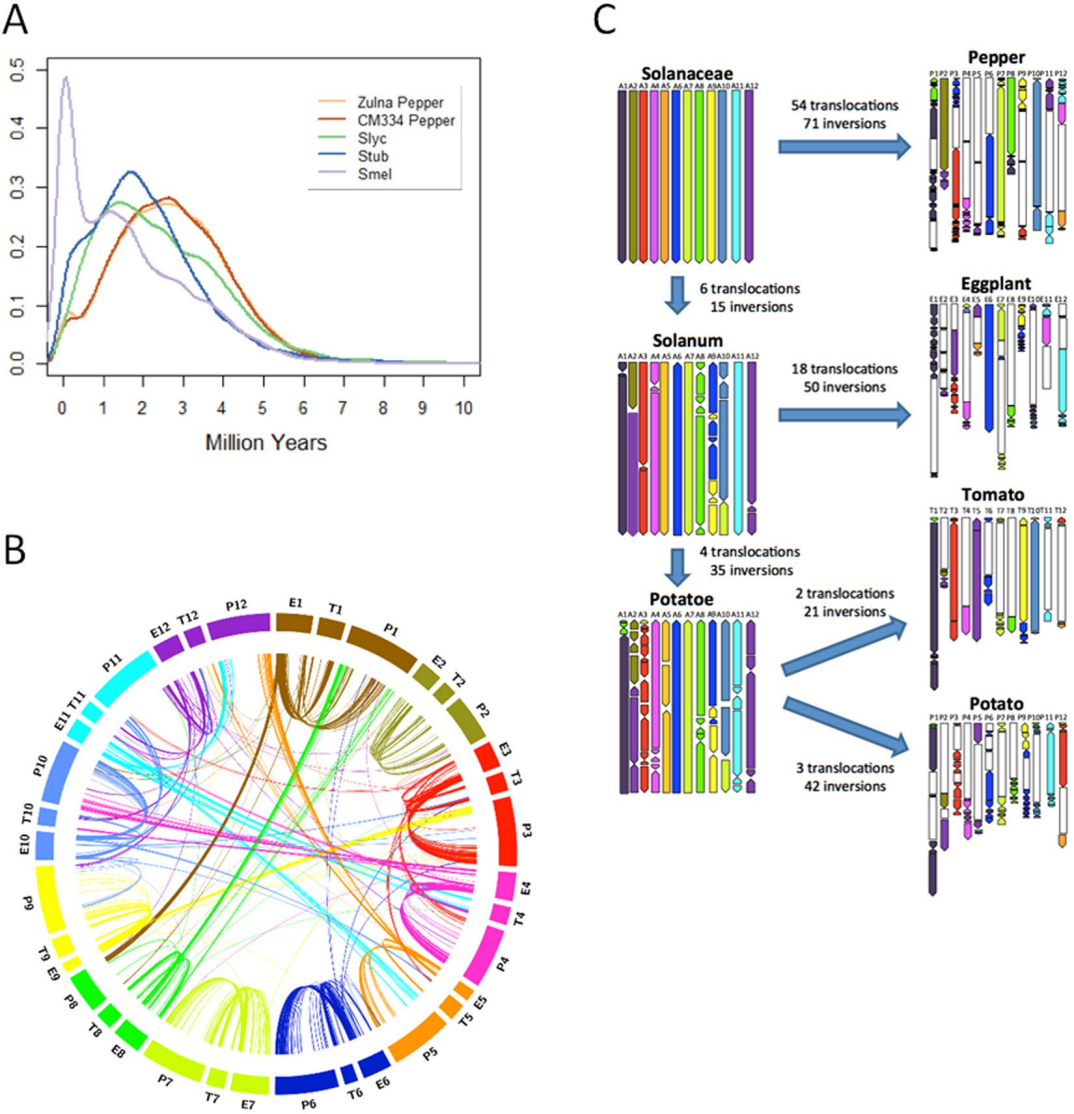
Solanaceae genome dynamics. (**A**) Dating of insertions for complete LTR retrotransposons in the various Solanaceae genomes. (**B**) Syntenic relationships between the chromosomes eggplant (E), tomato (T) and pepper (P). For the parameters used to define synthenic thresholds, see Supplementary Information 2.7. (**C**) Reconstruction of chromosome dynamics during Solanaceae evolution. Each chromosome of the hypothetical Solanaceae common ancestor is assigned a different colour. The reconstructed karyotypes of evolutionary intermediates are shown on the left, the actual karyotypes of extant species on the right. Chromosomal segments derived from each ancestral chromosome are painted with the corresponding colour, and the arrows indicate the orientation with respect to the ancestral chromosome segment. The minimum number of translocations and inversions occurring between two karyotypes are reported.

Although similar number of genes were found in the four genomes, the eggplant and pepper genome sizes are respectively ≈1.3-fold and ≈3.5 -fold larger than those of tomato and potato. Genome expansion of pepper was mainly attributed to a transposition burst of Gypsy LTR retrotransposons, whose dating varies from ≈13 mya^7^ to ≈0.3 mya^8^. We re-evaluated the transposon abundance and dating in the four Solanaceae genomes (Supplementary Information 2.2) and confirmed that larger genomes tend to be enriched in Gypsy, followed by Copia elements. The timing of the main burst of LTR transposition is ≈3 mya for pepper, ≈2 mya for tomato and potato, and ≈0.3 mya for eggplant (Fig. 2A), furthermore we confirmed the presence of multiple retrotransposition bursts as observed in both monocotyledons and dicotyledons^12,18–24^.

Like tomato and potato, both the eggplant and pepper genomes carry signs of the ‘T’ whole genome triplication first described in the tomato genome^6^, which we dated at 45 to 55 mya (Supplementary Information 2.7). The ‘T’ triplication occurred in the common ancestor of all Solanaceae, as confirmed by the petunia genome sequence^25^. A set of 3,234 eggplant, 5,099 tomato, 4,659 and 2,163 pepper ohnologs (paralogous genes generated by whole genome polyploidization) are still recognizable in the four genomes. Only 478 ohnologs share the same orthoMCL^16^ group in the four species, suggesting that genome fractionation following the ‘T’ triplication was lineage-specific. Gene Ontology (GO) enrichment analyses indicated that genes encoding transcription factors are selectively enriched in the extant ohnologs of eggplant, tomato, potato and *Capsicum* (Supplementary Information 2.7; Supplementary Table S27).

During evolution, the members of the Solanaceae family underwent inter- and intra-chromosomal translocations and inversions^6,26,27^, which are reflected in the synteny of the extant eggplant, tomato and pepper genomes (Fig. 2B). We used these genomes, plus that of potato and of the outgroup coffee^28^ to reconstruct chromosomal dynamics during Solanaceae evolution (Supplementary Information 2.7; Fig. 2C). Using parsimony analysis, we first reconstructed the ancestral genomes of the common Solanaceae, *Solanum* and *Potatoe* ancestors, and deduced the chromosomal rearrangements leading to the extant genomes in respect to their direct ancestor. *Capsicum* experienced the highest number of translocations and inversions (54 and 71, respectively), followed by eggplant (18 and 50), while the lowest number, with respect to the common ancestor *Potatoe*, was detected in potato (3 and 42) and tomato (2 and 21). Several lineage-specific rearrangements were identified: e.g. the translocation of A1 to chromosome (CH) 8 occurred one time both in the pepper and *Potatoe* lineages, so that pepper, tomato and potato CH1 carry fragments of the ancestral A1 and A8 chromosomes, while eggplant does not. Eggplant CH11 also carries a translocation between A4 and A11, not found in the other three genomes. By computing the frequencies of chromosomal inversions (2.87~5.7 per million years) and translocations (0.27~2.7 per million years), pepper and eggplant showed the highest translocation frequencies (2.7 and 1.22/million years, respectively), much higher than in previous estimates^26,27^.

### miRNA-based gene regulation

Using the MIReNA software^29^, we identified 158 high confidence miRNAs belonging to 42 families (Supplementary Information 2.5, Supplementary Table S20), of which 19 families are conserved in many taxonomic groups, while 3 (miR1919, miR5745 and miR6020) are mainly present in Solanaceae (Supplementary Table S21). Putative miRNA targets identified using Tapir^30^ in eggplant, resulted in the formation of 1,445 miRNA:mRNA duplex between 146 miRNA and 992 genes (Supplementary Table S22). The miRNA families targeting the highest number of genes were 172 and 156, targeting 483 and 144 genes respectively.

**Figure 3:**
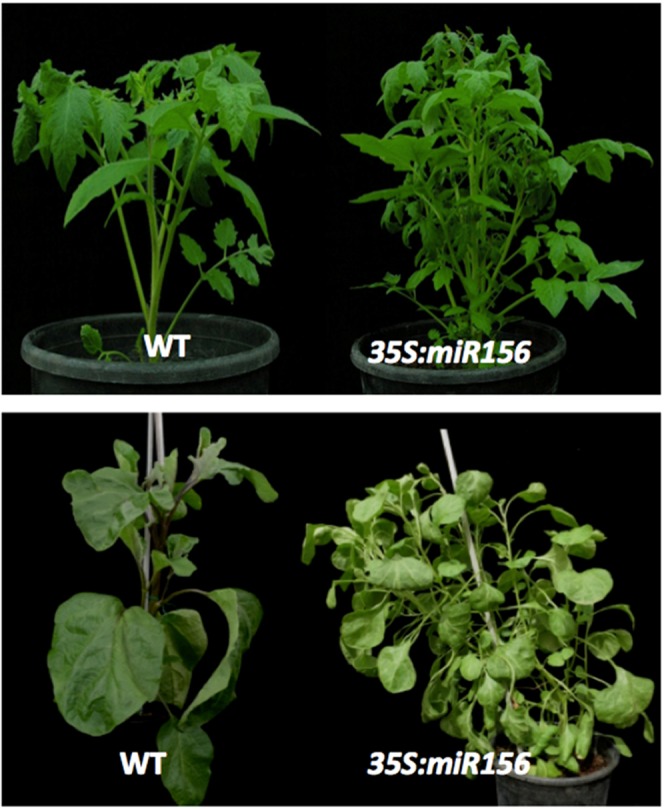
miR156 overexpression affects tomato and eggplant development in similar ways. MiR156 – mediated regulation of vegetative growth in tomato (upper panel) and eggplant (lower panel). Wild type plants (left) and plants overexpressing Arabidopsis miR156a (right) under the CaMV *35S* promoter.

We then zoomed into the function of miR156/157, which belongs to a highly conserved regulatory module in angiosperms, involving *SQUAMOSA PROMOTER BINDING* (*SPB*) genes^31^. The miRNAs 156/157 were predicted to target 9 *SPB* genes in tomato and 6 in eggplant (Supplementary Table S22). Both in eggplant and tomato, the ectopic expression of the *Arabidopsis* miR156/157 caused early release of apical dominance, delayed vegetative phase change (most evident in eggplant miR156/157 plants displaying a light pigmentation typical of the juvenile phase) and delayed onset of flowering (Fig. 3), in agreement with the conserved functions of the miR156/157*-SPB* module in these processes^31^.

### Regulation of fruit ripening, pigmentation, and cuticle biosynthesis

**Figure 4:**
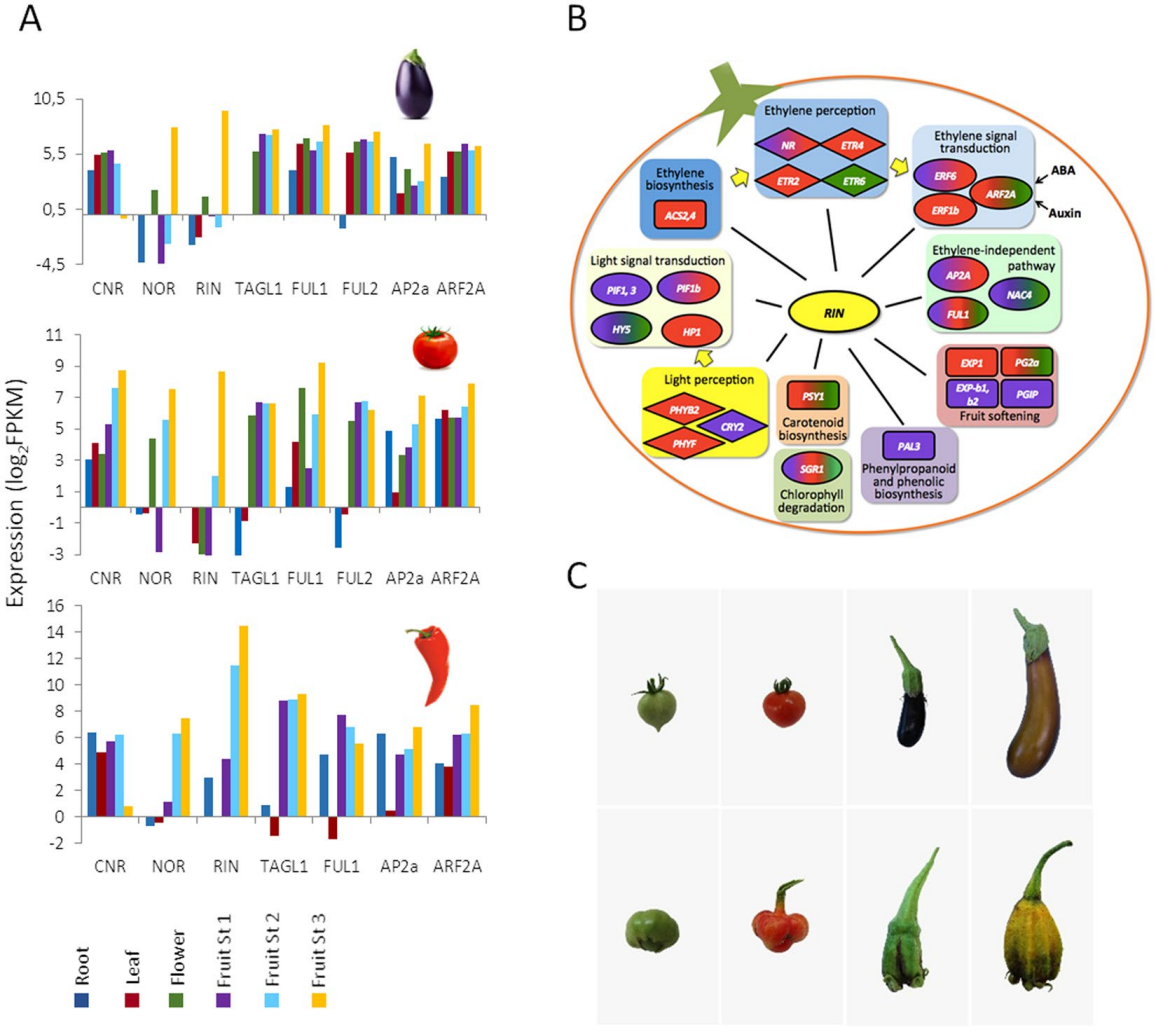
Evolution of fruit ripening control in the Solanaceae. (**A**) Expression (log_2_ FPKM) of ripening transcription factor regulators *CNR*, *NOR*, *RIN*, *TAGL1*, *FUL1/2*, *AP2a* and *ARF2A* in different tissue types (root, leaf and flower) plus different stages of fruit ripening (stage 1, stage 2 and stage 3) of eggplant (upper panel), tomato (middle panel) and pepper (lower panel). (**B**) Co-expression (R-value ≥ 0.8) of ripening -related genes with the *RIN* ripening regulator; ellipses, diamonds and rectangles indicate, respectively, transcription factor-, receptor-, and enzyme-encoding genes; purple, red and green shading indicate, respectively, co-expression with *RIN* in eggplant, tomato and pepper. Detailed co-expression values are shown in Supplementary Table S31. (**C**) Wild-type tomato fruit at two stages of ripening (upper left two panels); wild-type eggplant fruit at two stages of ripening (upper right two panels); and over-expression of the *TOMATO AGAMOUS-LIKE 1* (*TAGL1*) gene in tomato fruit at two ripening stages (lower left two panels) and in eggplant fruit at two ripening stages (lower right two panels).

We analyzed the expression pattern of the known tomato ripening regulators in tomato (ethylene-dependent ripening), eggplant and pepper (ethylene-independent ripening, Fig. 4A). Most of the transcripts, namely *RIPENING INHIBITOR* (*RIN*), *NON RIPENING* (*NOR*), *APETALA*2*a* (*AP2a*), *FRUITFULL1* and *2* (*FUL1-2*), displayed similar, ripening-associated expression patterns in all three species. *AUXIN RESPONSE FACTOR2* (*ARF2*) was ripening-associated only in pepper and tomato, and *COLORLESS NON-RIPENING* (*CNR*) only in tomato.

We also performed co-expression analysis of the whole complement of tomato, eggplant and pepper transcripts using as a bait *RIN*, a master regulator of ripening in both climacteric and non-climacteric fruits^32,33^ (Supplementary Table S31). The genes displaying high co-expression with *RIN* are shown in Fig. 4B. Genes involved in ethylene perception and signal transduction co-expressed across the three species. The *ACC SYNTHASE* genes (*ACS2* and *ACS4*) co-expressed with *RIN* only in tomato.

Light is a known regulator of fruit biochemical composition. Accordingly, *LONG HYPOCOTYL 5* (*HY5*) transcription factor^34^ was highly co-expressed with *RIN* in eggplant and pepper (Fig. 4B) and, to a lesser extent, in tomato (Supplementary Table S31). In contrast, cryptochrome and phytochrome photoreceptors as well as *PHYTOCHROME INTERACTING FACTORS* (*PIF*s) show a more species-specific regulation (Fig. 4B). *EXPANSIN* 1 (*EXP1*) and *POLYGALACTURONASE 2A* (*PG2A*) encode important cell wall-modifying enzymes implicated in tomato fruit softening^35^. The former showed significant co-expression with *RIN* only in tomato, while two *EXP* isoforms were co-expressed in eggplant and none in pepper (Fig. 4B). *PG2A* was co-expressed with *RIN* in tomato and pepper, but not in eggplant.

Consistent with the high content in phenolics of ripe eggplant fruits, an isoform of *PHENYLALANINE AMMONIA LYASE* (*PAL3*) was highly co-expressed with *RIN* in eggplant, but not in tomato and pepper (Fig. 4B). The *PHYTOENE SYNTHASE 1* (*PSY1*), the first dedicated step in carotenoid biosynthesis, showed high levels of co-expression with *RIN* in tomato and pepper, which are rich in these compounds, but not in eggplant. Lastly, the *STAY GREEN 1* (*SGR1*) transcription factor, involved in chlorophyll degradation^36^, was highly co-expressed with *RIN* across the three species, in which active chlorophyll degradation occurs during ripening (Fig. 4B).

Ectopic expression of the tomato *TAGL1* gene^37^ resulted in sepal inflation as well as other ripening-associated features in tomato and eggplant (Fig. 4C). The inflated sepals accumulate species-specific pigments: in tomato, at first chlorophyll and leaf-type carotenoids and then lycopene, while in eggplant at first anthocyanins and then orange chalcone and flavonols. This indicates that in both species, *TAGL1* likely controls the expression of similar sets of developmental genes, but different sets of pigmentation pathway genes.

**Figure 5:**
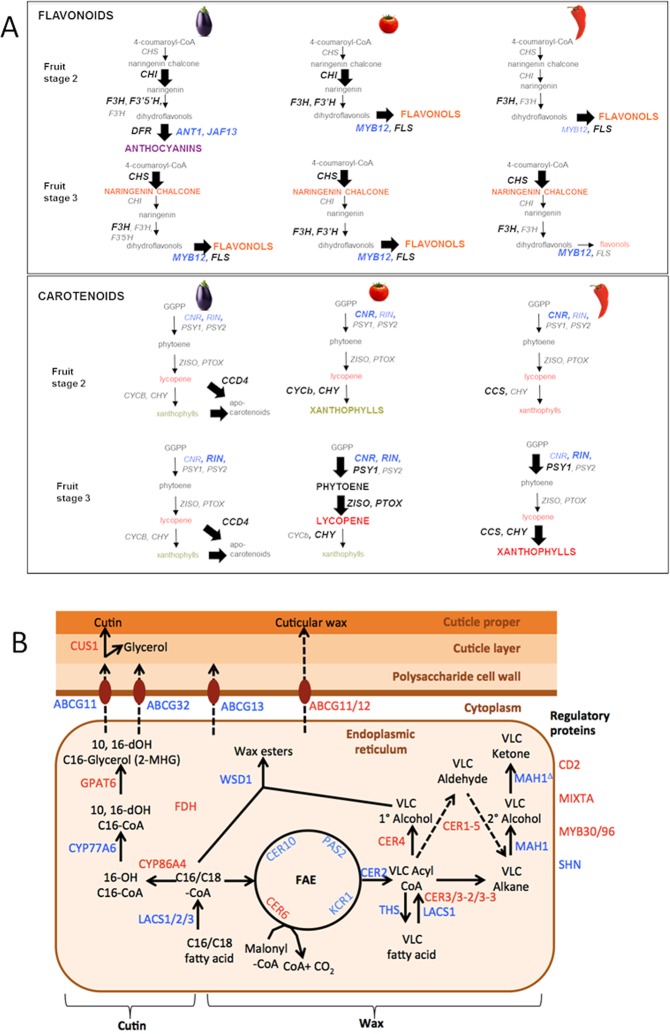
Control of fruit biochemical composition. (**A**) Schematic pathways for flavonoid (left) and carotenoid (right) biosynthesis in eggplant fruits. Genes and compounds overexpressed at each stage are shown in large bold character. Regulatory genes are indicated in blue. (**B**) Schematic cuticle biosynthesis pathway. Shortened gene names are indicated in blue. Genes showing fruit cuticle-enriched expression in both eggplant and tomato are indicated in red.

In eggplant, commercially ripe fruits (stage 2) accumulate mainly purple/black anthocyanins, while in physiologically ripe (stage 3) fruits the biosynthesis shifts towards orange-colored flavonoids such as naringenin chalcone (Chappell-Maor *et al*., unpublished data). Several phenylpropanoid biosynthesis genes contribute to the pigmentation pattern (Supplementary Information 3.4). The biosynthetic shift from anthocyanin to flavonoid pigments, occurring between stages 2 and 3, correlates with the down-regulation of the *ANTHOCYANIN1* (*ANT1*) and *JOHNANDFRANCESCA13* (*JAF13*) transcription factors and the *DIHYDROFLAVONOL 4-REDUCTASE* (*DFR*) structural gene as well as the up-regulation of the *MYB*12 and *FLAVONOL SYNTHASE* (FLS) genes (Fig. 5A). Most genes in the carotenoid pathway showed detectable, albeit low expression in ripening eggplant fruits, while *CAROTENOID CLEAVAGE DIOXYNENASE 4* (*CCD4*) was highly expressed throughout eggplant fruit ripening (Fig. 5A).

We identified orthologs of genes known to be involved in wax and/or cutin biosynthesis, and whose expression was enriched in fruit skin of tomato and eggplant (Supplementary Information 3.5). In several gene families, a single ortholog showed a similar degree of fruit skin enrichment in both species, such as *ECERIFERUM 6* (*CER6*), Cytochrome P450 (*CYP86A4*), *GLYCEROL-3-PHOSPHATE ACYLTRANSFERASE 6* (*GPAT6*) and *CUTIN SYNTHASE* (*CUS1*) in the cutin pathway, *FIDDLEHEAD* (*FDH*) in the wax biosynthesis, and the ABC transporters *ATP-BINDING CASSETTE G 11/12* (*ABCG11/12*) in transport to the fruit extracellular domain (Fig. 5B). Three transcription factors involved in cuticle formation in tomato and/or *Arabidopsis*, i.e. *CUTIN DEFICIENT 2* (*CD2*), *MYB30/96* and *MIXTA-LIKE*^38,39^ also showed skin-enriched expression in tomato and eggplant fruits.

### Evolution of pathogen resistance and glycoalkaloid biosynthesis

**Figure 6:**
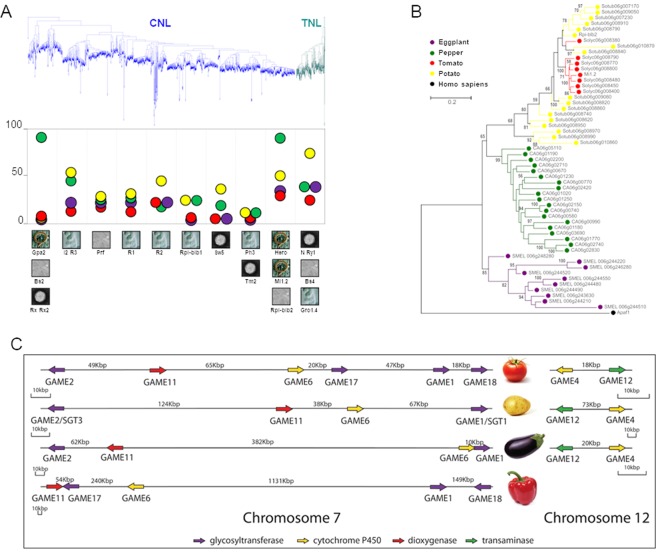
Gene family evolution in the Solanaceae. (**A**) Phylogenetic tree of CNL (in blue) and TNL (in green) *NB-LRR* genes. The distinctive evolutionary dynamics of ten *NB-LRR* groups in eggplant (purple), potato (yellow), tomato (red) and pepper (green) genomes are displayed. The number of *NB-LRR* genes included in each group is indicated on the vertical axis. The plant pathogens (fungi, virus, bacteria or nematodes) to which known genes in the clade confer resistance are indicated by pictures. *Gro1*.*4* and *Hero* confer resistance to *Globodera rostochiensis*. *Gpa2* and *Mi1*.*2* confer resistance to *Globodera pallida* and *Meloidogyne incognita*, respectively. *Rx* and *Rx2* confer resistance to *Potato Virus X*. *N*, *Ry1*, *Tm2* and *Sw5* confer resistance to Tobacco Mosaic Virus, Potato Virus Y, Tomato Mosaic Virus and Tomato Spotted Wilt Virus, respectively. *Bs2* and *Bs4* confer resistance to *Xanthomonas campestris* and *Prf* to *Pseudomonas syringae*. *Rpi-blb1*, *Rpi-blb2*, *R1*, *R2* and *R3* confer resistance to *Phytophthora infestans* and *I2* to *Fusarium oxysporum*. (**B**) Independent expansion of *Mi1*.*2*/*Rpi-blb2* homologous genes in eggplant, pepper, potato and tomato. The human APAF1 (Apoptotic Protease Activating Factor 1), is a human cytoplasmic protein showing similar structure to nucleotide binding site–leucine rich repeat proteins of plants, and was used to re-root the tree of plant NLR phylogenetic analysis^40,79^. (**C**) The steroidal glycoalkaloid metabolic gene cluster in four Solanaceous genomes. In tomato, six genes (*GAME1*, *GAME2*, *GAME6*, *GAME11*, *GAME17*, *GAME18*) are located on CH7 and two (*GAME4* and *GAME12*) on CH12. In potato, four-SGA associated genes (*SGT3*, *GAME6*, *GAME11* and *SGT1*/*GAME1*) are found in CH7 and two in CH12 (*GAME4* and *GAME12*). In eggplant, four putative SGA genes namely, *GAME1*/*SGT1*, *SGT3*, *GAME6* and *GAME11* are also physically linked to each other on CH7 and two, *GAME4* and *GAME12* on CH12. In pepper, five genes (*GAME1*, *GAME6*, *GAME11*, *GAME17* and *GAME18*) were found to be present on CH7. *GAME4* and *GAME12* homologous sequences were not detected in the pepper genome.

Annotation of main resistance protein classes highlighted significant amplifications in the four analyzed species^40^. The potato and pepper genomes showed a significant amplification of *NUCLEOTIDE-BINDING LEUCINE-RICH REPEAT* (*NB-LRR*) genes involved in pathogen defense (Supplementary Information 3.1). Two large expansions of *NB-LRR* genes, involving respectively the *Gpa2/Bs2/Rx/Rx2* and *Mi1*.*2/Hero/Rpi-blb2* subfamilies, are present in the pepper genome, while five subfamilies, including genes conferring resistance to *Phytophthora infestans*, are expanded in potato (Fig. 6A). Solanaceae genomes evolved preserving highly active R-islands in which the internal variability is regulated in species-specific manner. Species-specific diversification at individual resistance loci was mediated by tandem duplication of distinct founder paralogs in each species, as exemplified by the cluster on CH6, comprising the potato *Rpi-blb2* gene (resistance to *P*. *infestans*), and the tomato *Mi1*.*2* gene (nematode resistance) (Fig. 6B).

In the tomato and potato genomes, most core genes for Steroidal Glycoalkaloid (SGA) biosynthesis genes form two metabolic gene clusters, on CH7 and CH12^41^ (Supplementary Information 3.2; Fig. 6C). The cluster on CH7 was also found in eggplant and pepper, while the one on CH12, which contains *GLYCOALKALOID METABOLISM 4* and *12* (*GAME4 and GAME12*), only in eggplant (Fig. 6C). A BLAST search of the pepper genome did not yield any genes closely related to *GAME4* or *GAME12*. Since these two genes catalyze, respectively, the first and second step in the conversion of a furostanol-type saponin precursor into SGAs, their absence in pepper is likely responsible for the absence of SGAs in this species.

## Discussion

With around 450 species, the spiny Solanums represent the largest monophyletic group in the Solanaceae family. We obtained a high-quality, anchored eggplant genome sequence that fills an important gap for comparative genomics studies in the Solanaceae. The sequence was obtained through assembly of Illumina reads and further scaffolding/error correction using optical mapping. Gene annotation was assisted by RNA-Seq data from 19 different eggplant tissues/organs and resulted in 34,916 high-quality gene models, similarly to what was observed in other Solanaceae species.

### Chromosome dynamics and their contribution to Solanaceae genome diversity

We found signs of the ‘T’ triplication in the eggplant and pepper genomes and dated it between 45–55 mya, i.e. slightly more recent than previous estimates^6^. This, and the recent discovery of signs of the ‘T’ triplication also in petunia^25^, may indicate that Solanaceae radiation is more recent than previously reported. One of the main effects of the ‘T’ triplication is the generation of paralogous genes, or ohnologs, a fraction of which are still nowadays triplicated or duplicated. In such ohnologs, we detected an enrichment of genes encoding transcription factors. It has been suggested that one of the effects of gene-balanced polyploidizations is to leave behind duplicate “functional modules”, such as interacting transcription factor groups, which in turn increase morphological complexity^42^. This is what we observed in extant Solanaceae genomes, and may explain the extreme morphological variation and ecological adaptability of this plant family.

In the Solanaceae genomes analyzed, we detected signs of multiple retrotransposition bursts. The main burst in eggplant is the most recent (≈0.3 mya) while in pepper is the most ancient (≈3 mya). Since the main pepper burst occurred much later than the *Solanum*-*Capsicum* divergence (20 mya), our data did not confirm the hypothesis that retrotransposition bursts contributed to the reproductive isolation of different Solanaceae clades^7^. Using COS markers, 0.1~1 inversions per million years and 0.2~0.4 translocations per million years were estimated in the four lineages^26^, with the eggplant lineage experiencing an approximately double inversion rate than the other three. The frequencies we calculated, based on the whole genome sequences, were much higher for both translocations (0.27~2.7 per million years) and inversions (2.87~5.7 per million years). This is probably due to the higher resolving power of the high-quality genomes used in our analysis with respect to the COS maps. Compared to the other three Solanaceae, pepper shows a very high rate of putative translocations (2.7/million years), followed by eggplant (1.22/million years). Pepper and eggplant also carry the highest number of retrotransposons, suggesting that chromosomal translocations could have been mediated by recombination between homologous retrotransposons located on different chromosomes, as reported for yeast^43^.

An additional mechanism contributing to the functional plasticity of Solanaceae genomes is gene duplication, exemplified by R gene diversification, which occurred at very different rates in different species^44^. Tomato and eggplant show relatively low rates of R gene duplication, while potato and pepper show much higher ones. Tandem duplications of R genes are generally lineage-specific, with the majority of events occurring after the separation of the major Solanaceae clades, however our data also highlighted additional tandem duplications which resulted in eggplant-specific gene clusters sharing homology with characterized TNL resistance loci.

### Evolution of secondary metabolism

In angiosperms, gene clusters encoding enzymes for specialized secondary metabolites mediate the synthesis of defense compounds, such as hydroxamic acid derivatives, alkaloids, cyanogenic glucosides, and SGAs^45^. Several hypotheses may explain the evolution of these clusters, including co-regulation and/or co-inheritance of clustered genes. SGAs are involved in the defense against herbivores and are produced by numerous members of the *Solanum* genus, including tomato, potato and eggplant^46^ (Supplementary Information 3.2). In contrast, pepper does not produce glycoalkaloids but steroidal glycosides, saponins and capsaicinoids^47^. As previously reported in tomato and potato^41^, also in eggplant the SGAs biosynthesis genes are clustered on CH7 and CH12 and are co-regulated. However, the CH12 cluster, which encodes the first two dedicated steps in the SGA pathway after the common precursor of SGAs and steroidal saponins, is missing in pepper, suggesting that the gain/loss of this cluster served as an evolutionary switch mediating the rerouting of steroidal metabolism from steroidal saponins to SGAs and *vice versa*.

Pigmentation of fleshy fruits is strongly influenced by coevolution with the frugivorous animals that perform seed dispersal, with red and black fruits prevailing in plants whose seeds are dispersed by birds^48^. Our data indicate a similar regulation in tomato, potato and pepper fruits of the *STAY GREEN* gene, encoding a plastid-localized protein that enhances both chlorophyll degradation and carotenoid biosynthesis^36,49^. This, and the induction in fruits of all three species of a *PSY* gene which encode the rate-limiting step of carotenoid biosynthesis^49^, indicates that chlorophyll degradation and carotenoid biosynthesis is regulated in a similar way in the three species during fruit ripening. The lack of carotenoids in eggplant fruits can probably be attributed to high expression of carotenoid-cleaving enzymes such as CCD4, as already described in white peach fruits^50^.

Fruits, like other aerial plant parts, are coated with a lipophilic cuticle largely composed of waxes and cutin, which impacts many pre- and post- harvest processes including fruit water relations, expansion and the response to biotic and abiotic stresses^38^. Our data indicate that structural and regulatory genes controlling cuticle biosynthesis in tomato and/or Arabidopsis also showed skin-enriched expression in eggplant fruits, suggesting that the underlying regulatory network is highly conserved in eudicots.

### Fruit development and ripening

Tomato, eggplant and pepper fruits undergo physiological changes during ripening, which are ethylene-dependent in tomato and ethylene-independent in eggplant and pepper. Ripening of the tomato fruit is well studied, and is controlled by a complex signal transduction pathway, involving several transcriptional regulators^32^. Our transcriptional and co-expression studies suggest that more similarities than differences exist in the mechanisms controlling fruit ripening in different Solanaceae clades. The mRNAs encoding known regulators of ripening are upregulated during ripening in tomato, eggplant and pepper, with the exception of the *CNR* gene^51^, which is upregulated in climacteric tomato, but not in non-climacteric eggplant and pepper fruits. This observation partially contrasts with the proposed role of *CNR* in regulating ripening upstream of ethylene synthesis^52^. With the exception of *CNR*, the main ripening regulators appear to be regulated in a similar fashion in climacteric and non-climacteric fruits, and they also appear to have similar functions, as highlighted by the very similar developmental phenotypes obtained by ectopic expression of the *TAGL1* transcription factor in tomato and eggplant.

The main components of the network controlling fleshy fruit ripening across different Solanaceae include members of the ethylene receptor gene family, as well as *ETHYLENE* and *AUXIN RESPONSE FACTORS* (*ERF*s and *ARF*s) in both climacteric and non-climacteric fruits. Apart from *CNR*, the genes showing different regulation in climacteric versus non-climacteric fruits are those involved in ethylene biosynthesis (*ACS* and, to a lesser extent, *ACO*). This, together with the fact that climacteric fruit ripening in the Solanaceae is of polyphyletic origin,  suggests that the two different types of fruit ripening arose recently during evolution, through a modification in the regulation of relatively few components, including *CNR* and ethylene biosynthetic genes.

## Methods

### Sequencing, assembly and anchoring

The *S*. *melongena* 67/3 line was obtained as cross between ‘Purpura’ × ‘CIN2’ and 305E40 line was derived from the somatic hybrid *Solanum aethiopicum* gr. Gilo(+)*S*. *melongena* cv. Dourga, F5 and F6 progenies were derived from the cross between this two lines, 305E40 as female parent and 67/3 as male parent. High molecular weight nuclear DNA was extracted from leaf tissue of young plants according to Carrier *et al*.^53^ for 67/3 line and using a modified CTAB for lines 305E40 and the RILs population (Supplementary Information 1.1). Small-insert libraries were produced using the TruSeq DNA protocol and long-insert mate-pair libraries were prepared using the Nextera Mate Pair protocol. Libraries were sequenced on an Illumina HiSeq1000 instrument with 2 × 100 nt protocol at the Functional Genomics Centre, University of Verona, Italy) (Supplementary Information 1.2). The reads have been submitted to the NCBI Sequence Read Archive under the accession number SRP078398.

Raw reads underwent a quality filtering process (Supplementary Information 1.3) and error corrected using the SOAP error corrector (V1.00). Assembly and scaffolding were performed using SOAPdenovo2^10^ using a multi k-mer strategy. Gaps in scaffolds were filled with GapCloser (Supplementary Information 1.4). Quality of the assembly was assessed by BUSCO v3^15^ pipeline and by blast search of ESTs downloaded from NCBI (Supplementary Information 1.4). Next-generation genome map of the line ‘67/3’ was performed with BioNano technology at Bionano Genomics (San Diego, California, US), high-molecular-weight DNA was extracted from leaves, labeled and stained using the IrysPrep Kit (Supplementary Information 1.5). Maps were assembled from optical reads with IrysView software (Supplementary Information 1.5) and assembled with Illumina assembly data in hybrid scaffolds using HybridAssembler tool (Supplementary Information 1.5).

RILs segregation patterns were analyzed with SOILoCo pipeline^12^, and linkage analysis was performed with “R/qtl” package^54^ and ordered with Joinmap 4 software^55^ (Supplementary Information 1.5). Pseudomolecules were obtained by combining both linkage and optical mapping information (Supplementary Information 1.5). A *de novo* assembly of ‘305E40’ was generated with Abyss^56^ and aligned to ‘67/3’ genome assembly using BLAT^57^.

### Transcriptome sequencing, genome annotation and SNP functional classification

The 67/3 plants were grown in greenhouse at CREA-GB (Montanaso Lombardo, IT) in standard conditions. RNA from 20 tissues was isolated (Trizol®), directional libraries constructed (Illumina TruSeq Stranded mRNA Library Prep Kit) and sequenced on an Illumina HiSeq. 1000 sequencer. Transcripts were constructed with the Velvet + Oases^58^ pipeline and EvidentialGene (http://arthropods.eugenes.org/EvidentialGene/). MAKER-P^14^ pipeline was adopted, and only genes with an AED ≤ 0.48 were retained, whose quality was evaluated with different pipelines. RNA-Seq reads from each experiment were aligned to the eggplant genome using TopHat 2^59^ and expression values (FPKM) for each gene model calculated (Cufflinks 2^60^). Proteins function assignment was performed with Hmmer^61^ and InterProScan^62^. Finally, genetic differences between the reference genome and the ‘305E40’ genotype were evaluated with SnpEff suite^63^ (Supplementary Information 2.1–2.6).

### Comparative analyses among eggplant, tomato, potato and pepper

Eggplant, tomato^6^, potato^5^ and pepper^7^ TE-related repeats were masked by building up species specific *de novo* repeat libraries with RepeatModeler^64^ and combined with Repbase^65^-viridiplantae. The LTR dating pipeline was completed on eggplant, tomato^6^, potato^5^, and peppers^7,8^ following the methods described elsewhere^66^ (Supplementary Information 2.2).

Differential gene expression analyses were carried out by comparing FPKMs (Cufflinks 2^60^) of eggplant with those of tomato, potato and pepper^5–7^. RNA-Seq reads were aligned to the respective genomes with TopHat 2^59^ (Supplementary Information 2.3).

Putative insertions of organelle genes were identified by blasting the four Solanaceae proteomes against the NCBI database (plastidial and mitochondrial genes; Supplementary Information 2.3).

MIReNA^29^ was used to identify miRNA-coding sequences in the four Solanaceae, by homology with known miRNAs (miRBase 21^67^). Target and mimic genes of the identified miRNAs were spotted with Tapir^30^, and GO enrichments were obtained through AGRIGO^68^ (Supplementary Information 2.5).

The CoGe platform^69^ was used to detect orthologous genes among the four species, as well as ohnologs (for dating “T” triplication^6^). Ks-values were calculated for gene pairs using CodeML (PAML package^70^) implemented in SynMap^69^, and used to estimate the divergence time between the four Solanaceae (Supplementary Information 2.7).

The hypothetical ancestral chromosomes of the common ancestor of pepper, tomato, potato and eggplant, using coffee as an outgroup, were based on shared genes obtained from COGE^69^ outputs among the five species. GRIMM-Synteny^71^ was used to identify syntenic blocks among the 5 species, and were analysed with MGRA^72^ and ProCARs^73^ pipeline (Supplementary Information 2.7).

### Gene family analyses

The distribution of orthologous gene families was calculated using OrthoMCL^16^ version 2.0.9 on annotations from eggplant, pepper (PGA v1.55), tomato (iTAG v2.4), potato (iTAG v1) and Arabidopsis (TAIR10) (Supplementary Information 2.4). Orthologs were identified using CoGe^69^ (Supplementary Information 2.7); if not possible, putative orthologs were identified as best-hits by reciprocal BLAST (Supplementary Information 2.4).

A script developed in-house, based on a BLASTp analysis, was employed to identify eggplant (*Solanum melongena*) pathogen recognition proteins (PRPs) (Supplementary Information 3.1). The set of predicted PRPs identified was further analyzed using InterProScan^62^ software. The phylogenetic relationships of Solanaceae CNL and TNL proteins was calculated separately and similarities were determined performing a MAFFT^74^ (E-INS-i algorithm) multiple alignment. Clades were collapsed and numerated based on a bootstrap value over 85. Evolutionary analyses were conducted in MEGA6^75^.

The bootstrap consensus tree was inferred from 100 replicates. The PRGs cluster analysis was conducted using R software^76^. Evolutionary relationships were inferred by using the Maximum Likelihood method based on the JTT matrix-based model^77^. Heatmaps were produced using Genesis^78^.

Coexpression analysis was carried out with CoExpress (http://sablab.net/coexpress.html) using Tomato MADS-box encoding gene RIN and its orthologs in Eggplant and in Pepper as ‘baits’; the resulting lists of co-expressed genes which were filtered by r-value ≥ 0.6 (Supplementary Information 3.3).

## Supplementary information

Supplementary information

Supplementary Tables

## Data Availability

The reads have been submitted to the NCBI Sequence Read Archive under the accession number SRP078398. Upon acceptance, the assembly and annotation will be made available, in downloadable form, on GenBank and the Solanaceae Genome Network. Further information, including the ‘67/3’ genome assembly, pseudomolecules, annotations and Gbrowse are available through the website at www.eggplantgenome.org. For reviewing purposes, access can be obtained using the following credentials: User: anonymous; Password: geite0Ja. Eggplant biological materials can be requested to G.L.R. (giuseppeleonardo.rotino@crea.gov.it) and A.Ah. (asaph.aharoni@weizmann.ac.il).
