# Supplementary material for: A chromosome-anchored eggplant genome sequence reveals key events in Solanaceae evolution
Source: Sci Rep. 2019 Aug 13;9:11769. doi: 10.1038/s41598-019-47985-w (PMC6692341; doi:10.1038/s41598-019-47985-w)
Supplement: Supplementary file 1 — Supplementary information [file 41598_2019_47985_MOESM1_ESM.docx]

**SUPPLEMENTARY INFORMATION APPENDIX**

**A chromosome-anchored eggplant genome sequence reveals key events in Solanaceae evolution**

Lorenzo Barchi^1^, Marco Pietrella^2,3a^, Luca Venturini^4,5^, Andrea Minio^4^, Laura Toppino^3b^, Alberto Acquadro^1^, Giuseppe Andolfo^6^, Giuseppe Aprea^2^, Carla Avanzato^4^, Laura Bassolino^3b^, Cinzia Comino^1^, Alessandra Dal Molin^4^, Alberto Ferrarini^4^, Louise Chappell Maor^7^, Ezio Portis^1^, Sebastian Reyes-Chin-Wo^8^, Riccardo Rinaldi^1^, Tea Sala^3b^, Davide Scaglione^9^, Prashant Sonawane^7^, Paola Tononi^4^, Efrat Almekias-Siegl^7^, Elisa Zago^4^, Maria Raffaella Ercolano^6^, Asaph Aharoni^7^, Massimo Delledonne^4^, Giovanni Giuliano^2^*, Sergio Lanteri^1*^ and Giuseppe Leonardo Rotino^3b^

1. University of Torino - DISAFA - Plant Genetics and Breeding, Largo Braccini 2, 10095 Grugliasco, Torino, Italy
2. Italian National Agency for New Technologies, Energy and Sustainable Development (ENEA), Casaccia Res Ctr, Via Anguillarese 301 - 00123 Roma, Italy
3. Council for Agricultural Research and Economics (CREA), aResearch Center for Olive, Citrus and Tree Fruit 47121 Forlì, and bResearch Center for Genomics and Bioinformatics, 26836 Montanaso Lombardo (LO), Italy.
4. Department of Biotechnology, University of Verona, Strada Le Grazie 15, 37134 Verona, Italy.
5. Department of Life Sciences, Natural History Museum, Cromwell Rd, Kensington, London United Kingdom
6. Department of Agricultural Sciences, University of Naples Federico II, 80055 Portici, Italy
7. Department of Plant and Environmental Sciences, Weizmann Institute of Science, Rehovot 7610001, Israel
8. UC Davis Genome Center-GBSF, 451 Health Sciences Drive, University of California, Davis, CA 95616, USA
9. IGA Technology Services, Via J. Linussio, 51, 33100 Udine, Italy

*Corresponding authors

S1 Sequencing, assembly and anchoring 4

S1.1 Plant materials and preparation of high molecular weight (HMW) DNA 4

S1.2 Genomic DNA library preparation and sequencing 4

S1.3 Raw data processing and estimation of genome size 5

S1.4 Illumina Genome assembly and quality control 5

S1.5 Genome anchoring 6

S1.5.1 Next generation mapping 6

S1.5.2 Genome map analysis 6

S1.5.3 Hybrid Assembly and manual verification 7

S1.5.4 Linkage anchoring (SoiLoCO pipeline) 7

S1.5.5 Pseudomolecule construction after optical mapping 8

S1.6 Comparative analysis of parental lines genomes 9

S1.6.1 SNP/Indel mining, evaluation of functional SNPs and assessment of heterozygous SNPs in parental lines 9

S1.6.2 De novo assembly of the ‘305E40’ genome 10

S2.1 Transcriptome sequencing 11

S2.2 Transposable elements 12

S2.3 Annotation 15

S2.3.1 Expression analysis 15

S2.3.2 Functional annotation 16

S2.3.3 Validation of Annotation 17

S2.3.4 Identification of chloroplast and mitochondrial sequences 18

S2.4 OrthoMCL and besthit analyses 18

S2.5 miRNAs and miRNA target mRNA prediction 19

S2.6 SNP functional classification: impact on coding sequences 21

S2.7 Synteny, whole genome triplication analyses and ancestral genome reconstruction 22

S2.7.1 Synteny among the Solanaceae 22

S2.7.2 Ancestral Solanaceae genome reconstruction 24

S2.7.3 Ancestral genome evolution 27

S2.7.4 Species-specific ancestor genome evolution 28

S3 Targeted gene family analyses 28

S3.1 Disease resistance response genes 28

S3.2 Steroidal glycoalkaloids 29

S3.3 Fruit ripening regulators and coexpression analyses 31

S3.4 Pigment biosynthesis 31

S3.4.1 Phenylpropanoid biosynthesis 31

S3.4.2 Carotenoid biosynthesis 34

S3.5 Cuticle biosynthesis 34

SUPPLEMENTARY FIGURES 37

References 47

# S1 Sequencing, assembly and anchoring

## **S1.1 Plant materials and preparation of high molecular weight (HMW) DNA**

The *S. melongena* inbred line ‘67/3’ was obtained from an intra-specific cross between ‘Purpura’ x ‘CIN2’ followed by 9 cycles of selfing. The other parental line '305E40' is an androgenetic doubled haploid line, obtained through anther culture, derived from an inter-specific somatic hybrid between *Solanum aethiopicum* gr. *Gilo* and *S. melongena* cv. Dourga, and includes in its pedigree the inbred lines ‘DR2’ and ‘Tal1/1’. For whole genome sequencing, HMW nuclear DNA from line ‘67/3’ was extracted from isolated leaf nuclei starting from 6 grams of leaf tissue collected from young plants according to the protocol described by Carrier and colleagues^1^. Quality of isolated DNA was checked by spectrophotometric analysis by determining 260/280 and 260/230 ratios and integrity was assessed by electrophoresis on agarose gel (0.8%). For high density genetic map construction, 157 F6 RILs progenies derived from the cross between the two eggplant breeding lines ‘305E40’ (female parent) and ‘67/3’ (male parent), and differing from one another with respect to a number of key agronomic traits^2–4^, were used for genome anchoring via genetic linkage map construction using a genotyping by sequencing approach (GBS^5^). Nuclear DNA from lines ‘305E40’ and the RILs population was extracted from young leaves using a modified CTAB protocol^6^, and quality was assessed using PFGE.

## **S1.2 Genomic DNA library preparation and sequencing**

For the small-insert libraries, 1 µg of genomic DNA was sheared using the Covaris S220 instrument (Covaris Inc. Woburn, MA) and libraries were produced using the TruSeq DNA Sample Prep Kit (Illumina, San Diego, CA) according to manufacturer’s instructions. The ligated fragments were size selected at 400bp, 500bp and 600bp on 2% agarose gel prior to library enrichment by PCR. Long-insert mate-pair libraries (3.4Kb, 5Kb, 6.5Kb, 7.8Kb, 10Kb, 11Kb, 12Kb, 14.5Kb, 20Kb) were prepared using the Nextera Mate Pair kit (Illumina, San Diego, CA) according to the Gel-Plus manufacturer protocol starting from 4 μg HMW DNA. Quality control of the libraries was performed using an Agilent Bioanalyzer with the DNA High Sensitivity kit (Agilent Technologies, Germany). Libraries were quantified using the KAPA SYBR FAST Universal 2X qPCR Master Mix (Kapa Biosystems, Boston, MA, USA) and sequenced at the sequencing facility of the Functional Genomics Centre (University of Verona, Italy) as 2x100 nt runs on a HiSeq1000 instrument using the TruSeq PE Cluster v3 kit (Illumina, San Diego, CA) according to manufacturer’s, instructions yielding 153 Gb from standard paired-end (PE) libraries and 135 Gb from mate-pair (MP) libraries (**Supplementary Table S1**). The reads have been submitted to the NCBI Sequence Read Archive under the accession number SRP078398.

## **S1.3 Raw data processing and estimation of genome size**

To avoid assembly errors, low quality reads with more than 10% of undetermined bases (Ns) or with more than 50 bases called with a Phred-scored basecall quality < 7 were filtered out. Reads were then de-duplicated using a custom in-house script. To remove non-MP reads, data obtained from long-insert MP libraries was processed with Deloxer using default parameters^7^. Sequencing reads were then clipped with scythe (version 0.994;https://github.com/vsbuffalo/scythe) using default parameters and low quality 3' ends of reads were quality trimmed with sickle (https://github.com/vsbuffalo/sickle) using a quality threshold of 20 over a window of 10 bases. The genome size was estimated using a k-mer counting approach^8^. Filtered PE reads from 400bp and 500bp libraries of *S. melongena* line ‘67/3’ were divided into sliding short sequences (k-mers) of 23 bp, overlapping by 18 bp yielding 39.4billion k-mers. These were combined into a histogram, which revealed a major coverage peak at 32 (**Supplementary Figure S1a)** which showed heterozygous ratio is about 0.052, and indicated an expected genome size of at least 1.035 Gigabases (Gb). This value is likely an underestimate of the real genome size as suggested by the presence of secondary peaks in the distribution due to repeats. The genome size was also estimated by flow cytometry (**Supplementary Figure S1b)** as 2C=2.49 pg=2.43 Gb.

## **S1.4 Illumina Genome assembly and quality control**

The filtered PE reads were error corrected using the SOAP error corrector (V1.00) using “-k 23 -l 2 -e 1 -w 1 -q 30 -r 45 -j 1 -Q 64 -o 1” parameters. Assembly and scaffolding were performed using SOAPdenovo2 (V2.04) using a multi k-mer strategy^9^. Multiple assemblies were performed by using k-mers ranging from 71, 75, 79, 81, 83 as minimum values up to 95 and 99 as maximum values. An optimal k-mer value range from 83 to 95 was chosen based on maximization of N50 scaffold value and total assembly length. Contaminant-derived scaffolds were identified by the BLAST suite (BlastN) against Refseq bacterial, fungi and oomycetes databases, and removed. Finally, gaps in scaffolds were filled with GapCloser using “-l 150 -p 31” parameters (http://soap.genomics.org.cn/down/GapCloser_release_2011.tar.gz). The assembly metrics are shown in **Supplementary Table S2**.

To assess the completeness and correctness of gene regions in the assembly we used the BUSCO pipeline^10^. This algorithm performs a quantitative assessment of transcriptome completeness based on evolutionarily informed expectations of the presence of clade-specific sets of core genes using the near-universal single-copy orthologs selected from OrthoDB^11^. Default settings were applied with the option --genome and a plant-specific lineage profile (Embryophyta odb10 dataset, obtained by the software developers). Of the 1,375 genes of the dataset, 1,291 (93.9 %) could be identified within the assembled genome, with 1,278 of those (92.9 %) being full-length. However, since BUSCO analysis is limited to conserved genes, we also aligned 98.089 *S. melongena* ESTs downloaded from NCBI (GeneBank, downloaded on 2014/06/03) to the assembled genome. As a result, we confirmed that 93.5% of the sequences aligned to the *de novo* assembly of *S. melongena* genome.

## S1.5 Genome anchoring

### **S1.5.1 Next generation mapping**

The BioNano Genomics technology (Irys System) was used to construct a next-generation genome map of the line ‘67/3’. DNA preparation, data collection, and de novo assembly were performed at BioNano Genomics in San Diego. High-molecular-weight DNA was extracted from leaves as described^12^. Briefly, leaves where chopped with a razor, fixed with formaldehyde and blended with a tissue homogenizer. Nuclei were purified with a Percoll cushion and embedded into low-melting point agarose (Invitrogen) plugs. The plugs were treated with Proteinase K and RNase (Qiagen), washed, melted, solubilized with GELase (Epicentre), and drop dialyzed. DNA was quantified using the PicoGreen Assay Kit (Invitrogen). 300 ng of DNA was labeled and stained using the IrysPrep Kit (Bionano Genomics) according to the product manual^13^. 7U of endonuclease Nt.BspQI (New England BioLabs) were used, and a 20 min. 80°C nickase heat inactivation step following nicking was added. Loading of DNA into Irys Chip nanochannels via electrophoresis, and imaging of single DNA molecules were automated by the Irys system. The analysis was performed at the Bionano Genomics facility in San Diego, CA.

### S1.5.2 Genome map analysis

Image detection, molecule alignment, and assembly were performed using the IrysView software. Briefly, the DNA backbone and fluorescent labels were detected and converted into single-molecule genome maps. *De novo* assembly of genome maps was performed using a pairwise comparison followed by a graph-based assembly and five rounds of refinement and extension using moderately stringent arguments (>150Kb molecules and p-value thresholds of 1e^-9^ or better). The result of this assembly was used to estimate the error profile of the single molecule data by alignment of the single molecules to the consensus and these error values were used to improve the p-value calculation (this step is called autonoise) in a second round of *de novo* assembly, higher stringency was used (>150Kb molecules and p-value of 1e^-10^ or better). The result of the final *de novo* assembly was a 1.186 Gbp assembly with a map N50 of 2.56 Mb (**Supplementary Table S3**).

### S1.5.3 Hybrid Assembly and manual verification

The HybridAssembler tool from IrysView Software was used to assemble *in silico* digested sequence scaffolds and optical genome maps using the overlap of high scoring sequence motif pattern match groups^14^. Genome maps were aligned with sequence scaffolds and genome map lengths are calibrated. Conflicting alignments, i.e. those with match groups adjacent divergent patterns were identified and subsequently manually checked and corrected when possible. Out of 458 hybrid scaffolds, 101 (representing 36% of the hybrid assembly) showed 121 potential conflicts which would result in the total or partial exclusion of 119 genes from the hybrid scaffolds. Visual inspection of assembled optical maps, optical map reads, NGS scaffolds, PE read mappings and MP read mappings was employed to analyze and resolve the conflicts. Out of 121 conflicts detected, 5 were due to inconsistent assembly of optical maps without NGS sequences support, 103 were due to an error in the assembly of NGS sequences which resulted in chimeric NGS contigs with unreliable or absent support from PE and MP reads. The conflict was solved by splitting the erroneous NGS scaffold in correspondence of the chimeric junction. The remaining 13 conflicts showed no evident NGS scaffolding error and the Hybrid Scaffold sequence was not supported by any MP and PE read in correspondence of the conflicting junction. These conflicts were solved by splitting the hybrid scaffolds in correspondence of the conflict. Sequence contigs were finally merged according to overlapping matches and AGP and FASTA files are generated. This resulted in a 1.22Gb assembly consisting of 469 sequences with an N50 of 3.58 Mb. Final assembly statistics are reported in **Supplementary Table S3**.

### S1.5.4 Linkage anchoring (SoiLoCO pipeline)

Following the Illumina low coverage resequencing of a F6 mapping population of 157 recombinant inbred lines (RILs), obtained by the cross of the ‘67/3’ and the ‘305E40’ lines, the segregation information was generated for genome anchoring with adoption of the SOILoCo pipeline^15^. Parental lines were aligned on the assembly scaffolds with the BWA aln algorithm^15^ (using default parameters) and used to call SNP sites using the mpileup command integrated in Samtools^17^ with both alignments. We validated homozygous positions carried by the ‘67/3’ line contrasting to any polymorphism in the ‘305E40’ by retaining those positions with the genotype likelihood “PL” for the homozygous reference state equal to zero and the heterozygous state greater than 10. The progeny reads were aligned as described above and SNPs called in a multi-sample fashion as done for the two parents. SNP positions were then filtered by retaining only those as confirmed to be ‘67/3’ homozygous with any polymorphism in ‘305E40’. Positions were segmented in windows of 100 candidate segregating sites. The SoiLoCo script “vcf2string” was used to convert positions along windows in call strings ("https://bitbucket.org/dscaglione/soiloco") constraining to consider positions with allele frequency of 0.250 to 0.750 in the progeny. The ‘gt-hmm’ and ‘calls2csvr’ scripts were run with default values, only using a maximum ratio of missing data of 0.40 and an alpha to test segregation distortion set to 0.0001 in order to retain also mildly distorted loci.

Linkage analysis was performed by grouping phase-block markers with “R/qtl” package^18^, with minimum LOD=15, rec ≤ 0.10. For each linkage group identified, identical loci were removed with the Exclude identical function and the remaining loci were ordered with Joinmap software^19^ (version 4), using the Kosambi function^20^ to estimate distance and Maximum-likelihood function to infer correct order. The ordering step was iterated several times, each time by correcting genotype calls with the “SMOOTH.pl” script, which is a Perl implementation of the SMOOTH software as developed by van Os et al.^21^. Finally, visual inspection of genotypes was applied to identify and correct remaining genotype errors.

A total of 17,688 markers were generated and used for mapping purposes. Twelve major and 3 minor linkage groups (LGs) were identified. By blasting scaffolds against the previously developed RAD markers used to build the genetic map^22^, and considering syntenic information as well as information from optical mapping (see **S1.5.3** and **S2.7**), all the LGs were assigned to the eggplant chromosomes, although chromosomes (CH) 2, 8 and 11 each comprise two non-joined portions. Globally, 5,964 markers were placed on the genetic linkage map, covering 2,665.84 cM and corresponding to 2,073 unique scaffolds and 847.5 Mb of anchored sequence (**Supplementary Table S4**), which represents 72.8% of the 1,163 Mb genome. The scaffold position was identified by averaging all the genetic positions within a single phase block, while the correlation of genetic over physical distances with absolute Pearson coefficient > 0.50 allowed to infer scaffold orientation.

Most of the obtained scaffolds (72%) include sequences longer than 100Kb, with an average length of 405 Kb. The un-anchored portion of the genome is represented by 315.5 Mb (28.1%) and includes 9,131 scaffolds with an average length of 36 Kb. Globally, 26,366 genes (75.5%) are present in the anchored fraction of the genome.

### S1.5.5 Pseudomolecule construction after optical mapping

New pseudomolecules were obtained by combining both linkage and optical mapping information. Firstly, the map position of NGS scaffolds (1,290) in the 469 hybrid scaffolds were identified. For 89 hybrid scaffolds, it was not possible to find a position on the genetic linkage map, and thus they were not anchored to pseudomolecules. Furthermore, we did not use 4 hybrid scaffolds for which a unique map position could not been established.

Globally, 376 hybrid scaffolds containing 1,062 NGS scaffolds could be assigned to chromosomes. Finally, 1,120 NGS scaffolds not present in hybrid scaffolds could be assigned to chromosomes using their linkage map position.

The position of hybrid and NGS scaffolds on pseudomolecules was identified by averaging all the genetic positions from linkage map information, while the correlation of genetic over physical distances with absolute Pearson coefficient > 0.50 allowed to infer on scaffold orientation. Split chromosomes were joined, taking into account syntenic information and BlastP analyses (see Genome anchoring section). This resulted in: CH2A (top part) and CH2B (bottom part) in the final CH2, CH8A (top part) and CH8 (bottom part) in the final CH8 and CH11A (top part) and CH11 (bottom part) in the final CH11. The final eggplant pseudomolecules span 1.14Gb (gapped) of the eggplant genome, corresponding to 0.82 Gbp of ungapped sequence. On the other side, the unanchored portion of eggplant genome is 332 Mb (gapped; 250 Mb ungapped), with a N50 value of 3.18 Mb (**Supplementary Table S5**). We hypothesize that the unanchored sequences likely correspond to gaps in pseudomolecules and we estimate a total genome size of ~1.2 Gbp. Overall, 28,435 genes (81%) are present in the final anchored fraction of the genome.

## S1.6 Comparative analysis of parental lines genomes

### S1.6.1 SNP/Indel mining, evaluation of functional SNPs and assessment of heterozygous SNPs in parental lines

Both ‘67/3’ and ‘305E40’ reads were aligned against the assembled genome using BWA^16^ and mem command with default parameters. The bam file was processed with SAMtools mpileup (v1.1^17^) using default parameters with the exclusion of minimum mapping quality equal to 25. Results were filtered on the basis of two parameters: SNPs call quality and depth. SNPs having mapping quality lower than 20 were removed, while mapping depth lower and upper limit values were set to 8 and 200 respectively. The same parameters were applied for Indels detection.

In ‘67/3’ 309,097 SNPs/indels were identified, of which 10,633 were indels (**Supplementary Table S6**). The estimated heterozygosity of the reference eggplant genotype was 0.027% (heterozygous SNP frequency: 1/3,689 bp). The resequencing of ‘305E40’ led to identify 766,637 heterozygous SNPs/indels. The heterozygosity level in ‘305E40’ is 0.067% (heterozygous SNP frequency: 1/1,487 bp). Both values (0.027% and 0.067%) are comparable to the ones obtained in other inbred species.

Overall, the resequencing of ‘305E40’ revealed the presence of 2,605,011 SNPs and 93,590 small indels with respect to ‘67/3’ (**Supplementary Table S6**), corresponding to a frequency of 1 SNP every 436 bp and 1 indel every 12,184 bp. ^19^ was identified between domesticated and wild tomato. Finally, in potato^20^ one SNP every 40 bp/29bp and one insertion/deletion (indel) every 394 bp/253bp, according to genomes compared.A previous study^22^ aimed at developing RAD-tag markers of the same eggplant genotypes, estimated a SNP frequency of 1 SNP every 1,250 bp: presumably this discrepancy is due to RAD-tag protocol applied, which was based on the use of one methylation sensitive enzyme and the obtained metrics mainly refer to a reduced representation of the eggplant genome. The SNP frequency is similar, to the ones previously reported in other diploid Solanaceae. Kim et al.^23^ reported an average SNP/Indel frequency of 1 in 262/4,761 and 240/4,424bp between two *C. annuum* varieties and 1 SNP/Indel every 51/1,250bp between *C. annuum* and *C. chinense*. Qin et al.^24^ obtained a SNP frequency of 0.65% (one SNP every 153 bp) and an indel frequency of 0.02% (one indel every 4,833 bp) between the reference line Zunla (*C. annuum* line) and a *C. annuum* var. *glabriusculum* (considered as wild) line. In tomato, a SNP frequency of 0.6% was reported between *S. lycopersicum* and *S. pimpinellifolium*^25^. In potato^26^, much higher frequencies of one SNP every 40 bp/29bp and one indel every 394 bp/253bp were reported, according to the genomes compared.

### S1.6.2 De novo assembly of the ‘305E40’ genome

A de novo assembly of ‘305E40’ was generated with Abyss^27^ v.2.0 using ~39Gb (35.46X) of raw PE reads. The filtering and trimming pipeline recovered ~37,31Gb in about 375M reads for PE sequencing (33.9X). After evaluating different kmer length metrics, the one of 76 was chosen for genome assembly.

The final assembly covers about 1.09 Gbp in 1,838,452 scaffolds with a N50 of 6,919 bp. The assembly includes over 142,600 sequences longer than 1Kbp and more than 20,700 sequences longer than 10Kbp. To assess the validity of the newly assembled genome, the 34,916 protein models annotated in the ‘67/3’ genome were aligned using BLAT^28^ against the newly assembled ‘305E40’ genome. A total of 34,586 (99%) proteins were mapped to the *de novo* contigs with at least 95% of identity (and with at least 5% hit coverage). By applying more stringent criteria (98% of minimum identity) a total of 34,007 (97%) annotated proteins mapped to the ‘305E40’ assembly, values similar to the ones obtained in tomato by mapping *S. pimpinellifolium* against the *S. lycopersicum* assembly^25^.

To evaluate the quality of the contigs, BLAT^28^ was used to align the assembly of ‘305E40’ against the ‘67/3’ assembly (including CH0) after repeat masking using RepeatMasker^29^. The axtChain program in the chainNet package (v2)^30^ was run with default parameters to connect the BLAT alignments and create longer syntenic blocks. Then, to obtain a single-coverage chain file, which can reduce error when linking scaffolds into chromosomes, the procedure from UCSC^31^ was followed. Chains with aligned lengths that were less than 10% of their corresponding scaffolds were discarded. When more than one scaffold mapped to a specific locus in a reference chromosome, only the one with the longest aligned length was retained. Globally, 732.54 Mb (67%) of the ‘305E40’ assembly (in 202,439 contigs) were assigned to the reference genome (~50%; **Supplementary Table S7**), of which about 581.23 Mb (50.86%) to pseudomolecules, including 33,429 genes (96%) at 95% identity and 32,556 (93%) at 98% identity. On the other hand, by considering the ungapped genome the percentage of ‘67/3’ sequence covered by the ‘305E40’ assembly reached 69.20%. This percentage raised to 70.4% by considering only the pseudomolecules. As expected, the chromosome less covered by the ‘305E40’ assembly was CH2.

**S2 Annotation, polyploidization and synteny analyses**

## S2.1 Transcriptome sequencing

Samples of 20 tissues (**Supplementary Table S8**), including roots, cotyledons, flowers, leaves, stems and fruits at different developmental stages were collected from *S. melongena* line ‘67/3’. Root- tips tissues (of about 5 cm length) were collected from at least 10 different seedlings 8-10 days after germination of 40 chlorine-sterilized seeds sown in Petri dishes on wet paper and maintained at 25 ±2 °C under 16h illumination at 700 µE m^−2^ s^−1^. Cotyledons were collected from ten plantlets grown under greenhouse conditions at the 4-5th true leaf stage. For *Verticillium* inoculated and non-inoculated roots, plantlets were grown under greenhouse conditions in a 54 hole tray and artificially inoculated at the 2-3th true leaf stage by dipping the roots, cleaned from the peat, in a *Vd* conidial suspension of 1.0x10^6^/ml or in water (mock inoculation) for ten minutes and then re-transplanted. For each sample, roots were collected from ten plantlets 6 hours after inoculation (hpi), cleaned in sterile water and immediately frozen. For the other tissues, 20 plants were grown in pots (30 cm diameter) in greenhouse at CREA-ORL (Montanaso Lombardo) in standard conditions from March to September. Skin and flesh of the fruits were collected at the commercial and physiological ripening stage according to Mennella et al.^32^ (**Supplementary Figure S2)**. Samples of each tissue were collected from at least 5 different plants and pooled. All fresh tissues were immediately frozen in liquid nitrogen and stored at -80°C. Samples were ground in liquid nitrogen. Total RNA was isolated from 1 g of powdered material from each tissue using Trizol® (Life technologies, Carlsbad, USA) following the manufacturer protocol and stored at -80°C.

RNA was quantified with a Nanodrop 1000 spectrophotometer (Thermo Scientific, Wilmington, DE, USA) and its integrity assessed with the Bioanalyzer RNA 6000 Nano Kit (Agilent, Santa Clara, CA, USA) according to manufacturer’s instructions. The TruSeq Stranded mRNA Library Prep Kit (Illumina) was used for the construction of directional libraries (insert size 130–350bp), which were sequenced as 101x2 nt paired-end reads using the TruSeq PE Cluster Kits v3 kit (Illumina, San Diego, CA) and the TruSeq SBS kit v3 (Illumina, San Diego, CA) on an Illumina HiSeq 1000 sequencer, following manufacturer instructions. Sequencing resulted in an average number of 19 million fragments per sample (**Supplementary Table S8**). The reads have been submitted to the NCBI Sequence Read Archive under the accession number SRP078398.

In order to perform comparative analyses on gene expression for selected tissues on the aforementioned Solanaceae species, expression data were retrieved from the related genome projects^22,26,33^ and compared with those generated in this work. Tomato expression data were retrieved as-is, while raw data obtained from potato and pepper have been re-mapped on iTAG v1.0^33^ and PGA v1.55^23^ genome and annotations, respectively. Briefly, RNA-Seq reads from each experiment were trimmed with Trimmomatic v0.33^34^ using parameters ILLUMINACLIP:adapters.fa:2:30:10 LEADING:10 TRAILING:10 and aligned to the respective genome sequences using TopHat 2^35^ with the following parameters: --b2-very-sensitive, --no-novel-juncs, -i 10. FPKM (Fragments Per Kilobase of exon model per Million mapped reads) were calculated with the Cufflinks 2^36^ package using the following parameters: -u, --min-intron-length 30. For comparative expression analyses, public RNA-Seq data from *S. lycopersicum*, *S. tuberosum* and *C. annuum* were selected to represent similar developmental stages (root, leaf, fully open flower, immature fruit, and two ripening stages), and different stages were renamed using for comparative purposes (**Supplementary Table S9**) The resulting expression metrics, including those in eggplant, are reported in **Supplementary Table S10**.

Putative transcripts were constructed from the data obtained from the 20 eggplant RNA-Seq libraries using the Velvet+Oases pipeline^37^, producing in average 94,093 putative transcripts grouped in 23,632 genes (**Supplementary Table S11**). Datasets were merged and redundant sequences were collapsed into 39,408 primary isoforms, each putatively corresponding to a single gene, and 87,836 alternative isoforms, using EvidentialGene (http://arthropods.eugenes.org/EvidentialGene/). To define a set of gene models useful for training gene predictors, the primary transcripts were compared with the proteomes of the four following species: *N. benthamiana*, *S. lycopersicum*, *S. tuberosum* and *A. thaliana.* Only the ones with more than 50% identity and 99% reciprocal coverage with at least one known protein were retained The resulting 14,353 sequences were aligned against the reference genome using MAKER-P v. 2.31.3^38^ and only those with 95% identity, 95% coverage and 70% of the Exonerate maximal score threshold were retained. Predicted coding sequences were once again compared with the four above mentioned proteomes and, to eliminate any potential artifact introduced by MAKER-P^38^ a total of 8,751sequences with 50% identity and 99% reciprocal coverage with at least one protein were retained.

## S2.2 Transposable elements

Before performing gene prediction, the reference genome was masked using RepeatMasker^29^ using a combination of homology-based and *de novo* approaches. Firstly, RepeatModeler^39^ (version 1.0.5) was used to build up the *de novo* repeat library based on the ‘67/3’ genome. The new library was then combined with Repbase^40^viridiplantae (20.05) to identify TEs.

TEs were classified into two main classes (typical of plant genomes): Class I (retrotransposon elements) and Class II (DNA transposons). Class I elements primarily consisted of long terminal repeat (LTR) retroelements, short interspersed nuclear elements (SINEs) and long interspersed nuclear elements (LINEs). Class II included hAT, En-Spam, Mudra and related sequences. Globally ~73% of the eggplant genome was masked (**Supplementary Table S12**).

For a comparison, the same approach was used to mask the tomato (v2.5)^41^, potato (ITAGv1.0)^25^ and pepper^23^ genomes, resulting in 67,74%, 45.59% and 74.51% masking of ungapped genomic sequences respectively (**Supplementary Table S12**), in line with previously findings^23,24,26,33^. In eggplant the most represented TE class was LTR Gypsy (~48%), confirming that this class is the most widespread within the Solanaceae family.

The LTR dating pipeline was completed on *S. lycopersicum*^41^, *S. tuberosum*^26^, *C. annum* cv CM334^23^, *C. annum* cv Zulna^24^ and *S. melongena*. LTR elements were characterized and dated following the methods described elsewhere^42^. Genomic sequences were mined for LTR elements using the program ltr_harvest^43^ from the Genome Tools suite to obtain a dataset of predicted LTR elements (with conditions ‘-mintsd 4 -mindistltr 4000 -maxlenltr 4000 -outinner’). Ltr_digest^44^ was used to annotate the predicted elements for their main components (PPT, PBS) and the inner protein coding domains. For predictions of the inner protein coding domains, 43 domains were selected from PFam^45^ starting with those employed by Steinbiss^44^. Additional domains were searched using the terms “retrotransposon”, “reverse transcriptase”, “gag transposon”; and also obtaining domains related to the previews results through the PFam ‘domain organization’ tool. Sequence alignments for the Seed dataset and Representative Proteomes at 35% and 75% cut-off were downloaded and HMM’s were generated from them using HMMER2.3.2^45^. For clustering of the predicted elements each of the domains were grouped independently using VMatch^47^ and then LTR’s were grouped using the method used elsewhere^44^. Family classification of all the predicted elements was done as in Wicker et al.^48^, using BLASTn and BLASTx searches against the TREP database^49^. After clustering and annotating the predicted LTR elements into repeat families, the age was calculated as previously described^50,51^ with modifications. A multiple sequence alignment of all the 3′ LTR and 5′ LTR was done using Clustal-Omega 1.2^52^. The alignment was then used to calculate the estimated sequence divergence using BaseML from PAML^53^ based on the similarity between the LTR’s. The estimated divergence between the 3′ LTR and 5′ LTR of each element was used to calculate the age using the formula T = d/2r, with d as the divergence estimate and where r is 1 × 10−8 as calculated elsewhere^54^ for host-encoded genes and adjusted for the higher evolution rate of TEs compared with genes.

The total number of found complete LTR elements correlate with the genome size: the two pepper genomes (CM334 and Zulna) contain the highest number of elements (17,562 and 21,318), followed by eggplant (14,293), whereas Potato/Tomato are in the lower range (5,338 and 9,690, respectively). The difference in elements found between the two pepper genomes could be an assembly artifact, since the CM334 assembly covers 2.7Gb and the Zulna assembly 3.4Gb and would it would be expected that the more difficult portion to assemble is the repeat rich. On the other hand, there is a more significant difference between tomato and potato (double amount of elements in potato). Out of the total elements, for each genome it was possible to classify between 54-62% of them into Copia or Gypsy families. Proportion of Copia/Gypsy elements was variable across the genomes, larger genomes tend to be Gypsy rich (13-21% Copia elements against 38-48% Gypsy elements) while the smaller the genomes are more balanced (23-29% Copia elements against 28-31% Gypsy elements). Estimation of insertion date was done for all the LTR elements that could be classified into Copia or Gypsy.

The age distributions for the pepper species are very close, and dated around 3 Million years ago (mya) (**Figure 2A**). These findings suggest that there are no major differences in the repeat profile between these two varieties; in addition the estimated LTR insertion time was older with what previously reported by Qin et al.^24^ (estimated genome expansion around 0.3 mya) and younger with what reported by Kim et al.^23^ (estimated genome expansion around 12.7 mya). Tomato and potato overall conserve the same shape and ages in global insertion time (around 2 mya), with differences mostly in the scale which is related to the difference in number of elements present in the genome; this estimated overall insertion time is just slighter younger than previously estimations for tomato and potato (2.8 mya^25^).

Eggplant has a very different age profile, with a first burst of transposition ~1.5 mya and a more recent one, affecting both Gypsy and Copia elements, less than 0.5 mya. These latter phenomena seem to affect most of Copia subfamilies and some of the Gypsy subfamilies. Eggplant originated in the ‘old world’, while potato, tomato and pepper all originated in the American continent. The great burst of LTR elements which eggplant experienced around 0.3-0.5 mya might be related to some environmental stress in its centers of origin. Recent (0.3-4 mya) proliferation of retrotransposon families has been observed also in other plant species ^12,50,55–60^ and could be the consequence of genomic stress events imposed by environmental factors. Indeed, within 1-4 mya, a multi-peak burst for eggplant and tomato (at ≈ 0.5, 1.5, 3 and 3.5 mya; **Figure 2A**) as well as a distinguishable multi-peak profile for potato and pepper were detected.

## S2.3 Annotation

Prior to *ab initio* prediction, a set of 111 control genes was defined from manually curated coding sequences or from FL cDNA alignments (**Supplementary Table S13)**. Out of the 8,751 high quality gene models previously defined from the assembly and analysis of RNA-Seq data, 70 matched genes in the control gene set and were excluded. The remaining 8,681 gene models were used for the first round of training of 3 *ab initio* predictors: (i) GeneID^61,62^,(ii) Augustus^63^ and (iii) TwinScan^64^, accordingly to authors instructions. Accuracy of *ab initio* predictions was assessed by comparison with the 111 control genes.

The protein and EST sequences of eggplant, other Solanaceae and other well annotated species such as *Arabidopsis thaliana* were downloaded from public databases and aligned to the reference genome using the MAKER-P^38^ pipeline (**Supplementary Table S14**). Eggplant EST alignments with 95% of identity, 95% coverage, and 70% of Exonerate maximal score threshold were retained. Alignments of ESTs and proteins from other species were filtered for a coverage ≥ 70%, identity ≥50%, and Exonerate maximal score threshold≥ 70%. The alignments were then filtered to remove any BLAST alignment with an intron longer than 10 Kb so that to minimize possible chimeric alignments.

The MAKER-P^38^ pipeline was applied to integrate the gene models from *ab initio* prediction, with proteins and EST alignments. Moreover, 127,244 contigs produced by *de novo* assembly of RNA-Seq data (see **S2.1**), and representing 39,408 primary and 87,836 alternative isoforms, were also provided to MAKER-P pipeline for alignment with 95% identity, 95% coverage and 70% of the Exonerate maximal score thresholds set. The annotation produced 48,412 transcripts in 44,618 gene loci. For the final annotation, only gene models with an Annotation Edit Distance (AED) of 0.48 or lower were retained. The 99% of the transcripts with a clear ortholog in another proteome (50% identity or higher, 99% or higher reciprocal BLASTP coverage, length ratio between 99% and 101%) showed an AED lower or than this threshold. By filtering the whole annotation, a final dataset of 34,916 gene models was obtained. Alternative transcripts were excluded from the final annotation. Final annotation metrics and their comparison to other Solanaceae are shown in **Supplementary Table S15**.

### S2.3.1 Expression analysis

RNA-Seq reads from each experiment were aligned to the eggplant genome sequences using TopHat 2^35^ with “--b2-very-sensitive” parameter set. Following alignment, expression values for each gene model in the *S. melongena* annotation were calculated as FPKM (Fragments Per Kilobase of exon model per Million mapped reads) with *cuffnorm* program in Cufflinks 2^36^ package using “-library-norm-method geometric” parameter^65^ (**Supplementary Table S16**). Equal numbers of reads from skin and flesh libraries were combined for fruit samples at stage B and C before alignment.

### S2.3.2 Functional annotation

The assignment of gene functions to protein sequences was performed via phmmer from the Hmmer package^46^ (3.1.b1), on Swissprot^66^ and TrEMBL^66^ databases, using default parameters with the exception of sequence E-value = e-10 and domain E-value = e^-5^. InterProScan^67^ (version 5.9-50.0) was used to scan protein sequences against the protein signatures from InterPro^67^ (version 50.0). The latter integrates protein families, domains and functional sites from different databases: Pfam^45^ (Ver 27.0), PROSITE^68^ (20.105), PRINTS^69^(42), ProDom^70^ (2006.1), SMART^71^ (6.2), TIGRFAMs^72^ (13), PIRSF^73^ (3.01), SUPERFAMILY^74,75^ (1.75), Gene3D^76^ (3.5.0), and PANTHER^77^ (9.0). InterProScan integrates the searching algorithms of all these databases. Based on Hmmer UniProtKB/Swiss-Prot search (higher confidence database), the highest score protein description was assigned to each query. Afterwards, information from Trembl analysis was added to proteins lacking gene-function description. Filters and corrections include a black list of uninformative words, e.g. ”hypothetical protein”, “Putative” or “similarity to”. When InterProScan results were available, the domain name and IPR codes was extracted and appended to the description line.

Globally, a description of 28,858 genes of the 34,916 predicted eggplant genes was assigned. InterProScan identified 193,950 protein domains and, on the whole, ~76% of the genes (26,411 out of 34,916) were assigned with at least one domain (6,408 unique IPR domain). The top 20 SUPERFAMILY domains are listed in **Supplementary Table S17.** The most abundant superfamily domain was SSF52540 (P-loop containing nucleoside triphosphate hydrolase); this superfamily is involved in several UniPathways, like chlorophyll biosynthesis or coenzyme A biosynthesis. By comparing the top 20 superfamily domains of eggplant and tomato^25^ (**Supplementary Table S17**), some species-enriched domains were found. In particular, domain SSF53098 (Ribonuclease H-like domain) was found to be the most abundant exclusive eggplant superfamily domain, which includes proteins involved in many biological processes, including replication, homologous recombination, DNA repair, transposition, RNA interference, and self-incompatibility.

A total of 54,781 (1,886 unique) GO^78^ terms were assigned to 19,622 (56%) of the 34,916 eggplant proteins. The mapping of GO terms to Plant GO slim categories allowed to highlight that the most representative GO slim terms for ‘Biological process’ were: GO:0008152 (metabolic process), GO:0009987 (cellular process), GO:0008150 (biological process), and GO:0009058 (biosynthetic process). For ‘Cellular component’ the most representative GO slim terms were GO:0005575 (cellular component), GO:0016020 (membrane), GO:0005622 (intracellular), and GO:0005623 (cell), while for ‘Molecular function’: GO:0005488 (binding), GO:0005515 (protein binding), GO:0000166 (nucleotide binding), and GO:0003824 (catalytic activity).

### S2.3.3 Validation of Annotation

In order to evaluate the quality of the annotation, in terms of both completeness and accuracy, the predicted protein sets were validated; (i) against the Arabidopsis TAIR10 annotation; (ii) against a set of eukaryotic conserved genes and (iii) by using single-copy orthologs.

First, eggplant proteins (34,916 loci) alongside with those of tomato (ITAG2.4^33^, 34,725 proteins), potato (ITAG v1^33^, 35,004 proteins), pepper (PGA v1.55^23^, 34,899 proteins) and the eggplant annotation generated in Hirakawa et al.^79^Sme_r2.5.1, 42,035 proteins) were compared against the Arabidopsis nuclear proteome (TAIR10^80^, 27,206 proteins) by BLASTP and the relative length ratios of the best protein pairs was computed. The Arabidopsis annotation was chosen as a comparison due to its high quality level. Similar to other Solanaceae (**Supplementary Figure S3A**), most eggplant proteins clustered within the 1-1.1 bins, thus showing similar lengths to their Arabidopsis counterparts. A low percentage of proteins (ca. 2%) showed lengths about twice the Arabidopsis ortholog. Proteins not finding a hit to the Arabidopsis proteome were retrieved in similar numbers in all Solanaceae annotations, with the exception of the eggplant annotation produced by Kazusa^79^, which had an unusually high number of such proteins, some of which are probably due to annotation artifacts.

In a second approach, aimed at checking completeness of the annotation, the Arabidopsis Core Eukaryotic Genes^81^ (CEGs, n= 248) were blasted to the various annotations (**Supplementary Figure S3B**). Again, most Solanaceae annotations contained very high proportions of the CEG proteins (≥247) with the exception of the Kazusa and pepper ones.

The third approach was based on the use of the near-universal single-copy orthologs selected from OrthoDB^11^ and exploited in the software BUSCO^10^. This algorithm performs a quantitative assessment of transcriptome completeness based on evolutionarily informed expectations of the presence of clade-specific sets of core genes. Default settings were applied with the option –trans and a plant-specific lineage profile (Embryophyta odb10 dataset) was obtained with the software developers for assessing BUSCO-specific profiles. Again, the results (**Supplementary Figure S3C**) highlight a higher number of complete single copy proteins in our annotation and the iTAG potato and tomato ones, and a lower one in the pepper and eggplant (Kazusa) ones.

### S2.3.4 Identification of chloroplast and mitochondrial sequences

To evaluate the number of putative insertions of chloroplast and mitochondrial genes, eggplant, tomato, potato and pepper predicted proteins were compared with the NCBI database of plastidial and mitochondrial genes (http://www.ncbi.nlm.nih.gov/genome/organelle/") using BLASTP (e-value < E-10). In eggplant, 1,713 and 1,302 predicted proteins (2,058 unique) showed a significant hit against chloroplastic and mitochondrial proteins, respectively. Of these, 1,132 chloroplastic and 821 mitochondrial predicted proteins were encoded by genes located on sequences anchored on chromosomes. For comparison, 1,127, 1,259 and 1,760 predicted proteins had a significant hit against chloroplastic proteins for potato, tomato and pepper respectively. Of these, 1,127, 1,237 and 1,488 were on sequences anchored on chromosomes. Significant hits against mitochondrial proteins were found for 995, 952, and 1,781 predicted proteins for potato, tomato and pepper respectively. Of these 995, 883 and 1,485 were anchored on chromosomes.

We searched the assembly for scaffolds belonging to the mitochondrial genome, using the tomato mitochondrial genomes (accession n. NC_035963.1). However, due to the use of nuclear DNA for preparation of the libraries, no scaffold covering the whole mitochondrial genome was retrieved.

## S2.4 OrthoMCL and besthit analyses

A total of 131,315 protein sequences from eggplant, pepper, tomato, potato and Arabidopsis were clustered into 23,123 gene families (except singletons) using OrthoMCL^81^ version 2.0.9 (**Supplementary Figure S4, Supplementary Table S18**). Among the 34,916 protein-coding sequences predicted in eggplant, 27,128 were clustered in 17,774 families. A total of 3,194 families containing 17,798 genes were exclusive of the four Solanaceae, 353 families (1,663 genes) from berry-producing species (eggplant, tomato and pepper), and 1,100 families (3,844 genes) from the *Solanum* species (eggplant, tomato and potato), 667, 422, 526, 795 and 1,004 families were unique to eggplant, tomato, potato, pepper and Arabidopsis, respectively. The 669 eggplant-specific clusters contained 1,964 genes of which 857 have at least one InterPro domain. The eggplant sequences which did not fall into any cluster (singletons) made up a total of 7,788 genes of which 4,080 have at least one InterPro domain.

Since OrthoMCL analysis does not identify the best hit for a given protein in different annotations, Blast^82^ was used to find the best hits for *S. melongena* proteins in *S. lycopersicum*, *S. tuberosum* and *C. annuum* coding sequences using a threshold e-value set at 1e^-3^. These matches were then used as a query for a reciprocal BLAST and the results were tabled using a custom script. As a result, a total of 32,736 (21,217 unique hits) orthologous gene sets were found with tomato, 32,383 (21,168 unique) with potato, 31,599 (19,051 unique) with pepper and 29,373 (14,413 unique) with Arabidopsis (**Supplementary** **Table S19)**.

## S2.5 miRNA**s and** miRNA target mRNA prediction

The MIReNA software^83^ was used for the identification of high confidence miRNA-coding sequences (miRBase release 21^84^ and slymirna157^85^) in each pseudomolecule and CH0, by homology with known miRNAs from an array of 13 species (plants and algae) including: *Solanum lycopersicum, Solanum tuberosum, Nicotiana tabacum, Vitis vinifera, Arabidopsis thaliana, Oryza sativa, Populus trichocarpa, Medicago truncatula, Zea mays, Picea abies, Triticum aestivum, Physcomitrella patens, Chlamydomonas reinhardtii.* MIReNA was run with default parameters and the maximum number of allowed mismatches between known miRNAs and putative miRNAs was set to 10. From the search against miRBase 21^83^ high confidence database, 158 *Solanum melongena* miRNAs (identified in 152 genomic regions and coded by 144 unique pre-miRNA sequences from 42 families) were predicted and used for further analyses (**Supplementary Table S20**).

The eggplant miRNA sequences identified were named on the basis of the miRNA family with the addition of *sme* suffix. miRNA families identified in eggplant are deeply conserved among plant phyla. Based on phylogenetic classification performed by Cuperus et al.^88^ and the work by Chávez Montes et al.^87^, 19 families are largely present in many taxonomic groups, while one is poorly enriched in monocots, 2 are enriched in dicots and 3 (miR1919, miR5745, miR6020) are mainly present in the Solanaceae family (**Supplementary Table S21**).

The Tapir standalone software^88^ was applied to identify the targets of the identified eggplant miRNA on the predicted *S. melongena* CDS as well as 3’UTR.

The Tapir_hybrid search for target genes of the 158 eggplant miRNAs identified 1,445 putatively miRNA:mRNA duplex formations between 146 miRNA and 992 genes (**Supplementary Table S22**). Approximately 90% genes encoding predicted target transcripts have functional InterPro annotations. The family mainly involved in miRNA:mRNA duplex formation was 172, which interacts with 316 transcripts, followed by 156, which interacts with 164 transcripts.

To identify putative gene enrichment, AGRIGO^89^ was used on the miRNA target sequences, using the whole eggplant GO terms identified with the Interproscan^67^ pipeline. For the ontology class ‘biological process’ the top 5 enriched terms (on the basis of the p value) were GO:0009808 (lignin metabolic process), GO:0046274 (lignin catabolic process), GO:0046271 (phenylpropanoid catabolic process), GO:0009698 (phenylpropanoid metabolic process) and GO:0042219 (cellular amino acid derivative catabolic process). For the ontology class ‘cellular component’, the top 5 enriched terms (base on p-value) were GO:0048046 (apoplast), GO:0005634 (nucleus), GO:0030123 (AP-3 adaptor complex), GO:0005576 (extracellular region), and GO:0030117 (membrane coat), while for the ‘molecular function’ the top 5 enriched terms (base on p-value) were GO:0030554 (adenyl nucleotide binding), GO:0032559 (adenyl ribonucleotide binding), GO:0032555 (purine ribonucleotide binding), GO:0032553 (ribonucleotide binding) and. GO:0001883 (purine nucleoside binding).

The same target identification procedure carried out for eggplant was conducted also in tomato (including 3’UTR sequences), potato and pepper. miRNA sequences were identified with MIReNA software^83^. In tomato, the Tapir_hybrid^88^ search for target genes of the 146 tomato miRNAs identified 1,415 putatively miRNA:mRNA duplex formation between 128 miRNA and 1076 genes (**Supplementary Table S22**). The family mainly involved in miRNA:mRNA duplex formation was 172, which interacts with 439 genes, followed by 396, which interacts with 146 genes. In pepper, out of the 143 miRNAs, 117 miRNA were putatively involved in 1,139 miRNA:mRNA duplex formation with 995 transcripts (**Supplementary Table S22**). The family mainly involved in miRNA:mRNA duplex formation was 169, which interacts with 202 genes, followed by 167, which interacts with 74 genes. Finally, in potato, out of the 170 miRNAs available, 1,383 putatively miRNA:mRNA duplex formation between 133 miRNA and 1,015 genes were identified (**Supplementary Table S20**). The family mainly involved in miRNA:mRNA duplex formation was 172, which interacts with 483 genes, followed by 156, which interacts with 144 genes.

To identify putative gene enrichment, AGRIGO^89^ was used on the miRNA target and mimic sequences. InterProScan^67^ (version 5.9-50.0) was used to scan protein sequences of pepper, tomato and potato against the protein signatures from InterPro to retrieve GO terms. For target tomato genes, regarding the ontology class ‘biological process’ the top 5 enriched terms (on the basis of the p value) were GO:0007275 (multicellular organismal development), GO:0032502 (developmental process), GO:0032501 (multicellular organismal process), GO:0007165 (signal transduction), and GO:0023046 (signaling process). For the ontology class ‘cellular component, the top 5 enriched terms (base on p-value) were GO:0005634 (nucleus), GO:0043231 (intracellular membrane-bounded organelle),GO:0043227 (membrane-bounded organelle), GO:0031974 (membrane-enclosed lumen) and GO:0043233 (organelle lumen), while for the ‘molecular function’ the top 5 enriched terms (base on p-value) were GO:0005515 (protein binding), GO:0043531 (ADP binding), GO:0030554 (adenyl nucleotide binding), GO:0017076 (purine nucleotide binding), and GO:0032555 (purine ribonucleotide binding).

For target pepper genes, regarding the ontology class ‘biological process’ the top 5 enriched terms (on the basis of the p value) were GO:0032502 (developmental process), GO:0032501 (multicellular organismal process), GO:0023052 (signalling), GO:0009808 (lignin metabolic process), and GO:0046274 (lignin catabolic process). For the ontology class ‘cellular component’, the top 5 enriched terms (base on p-value) were GO:0005634 (nucleus), GO:0048046 (apoplast), GO:0031224 (intrinsic to membrane), GO:0016021 (integral to membrane), and GO:0009522 (photosystem I), while for the ‘molecular function’ the top 5 enriched terms (base on p-value) were GO:0043531 (ADP binding), GO:0032559 (adenyl ribonucleotide binding), GO:0030554 (adenyl nucleotide binding), GO:0032555 (purine ribonucleotide binding), and GO:0032553 (ribonucleotide binding).

For target potato genes, concerning the ontology class ‘biological process’ the top 5 enriched terms (on the basis of the p value) were GO:0009808 (lignin metabolic process), GO:0046274 (lignin catabolic process), GO:0046271 (phenylpropanoid catabolic process), GO:0009698 (phenylpropanoid metabolic process), and GO:0042219 (cellular amino acid derivative catabolic process). For the ontology class ‘cellular component’, the top 3 enriched terms (base on p-value) were GO:0048046 (apoplast), GO0005576 (extracellular region), and GO:0005634 (nucleus), while for the class ‘molecular function’ the top 5 enriched terms (base on p-value) were GO:0043531 (ADP binding), GO:0032559 (adenyl ribonucleotide binding), GO:0032559 (purine ribonucleotide binding), GO:0032553 (ribonucleotide binding), and GO:0030554 (adenyl nucleotide binding).

## S**2.6 SNP functional classification: impact on coding sequences**

The analysis of genetic differences between the ‘67/3’ reference genome and the ‘305E40’ genotype was conducted using the SnpEff suite^90^. In ‘67/3’ the role of missense and non-sense mutations in heterozygous state was investigated, while in ‘305E40’ was possible to highlight missense and non-sense mutations in both homozygous/heterozygous states (**Supplementary Table S23**).

*Mutations in ‘67/3’ genes.* A total of 6,564 heterozygous SNPs/indels were detected in the annotated gene space of the ‘67/3’ genotype (**Supplementary Table S23**). A total of 2,078 SNPs/indels were silent, 4,377 were classified as missense while 109 resulted as nonsense mutations.

*Mutations in ‘305E40’ genes.* A total of 28,768 SNPs/indels were observed in the annotated gene space of which 11,365 were heterozygous while 17,403 homozygous (**Supplementary Table S23**). A total of 9,491 SNPs/indels (5,910 homozygous; 3,581 heterozygous) were silent, 18,579 (11,097 homozygous; 7,482 heterozygous) were classified as missense and 698 (396 homozygous; 302 heterozygous) resulted as nonsense mutations.

## S2.7 Synteny, whole genome triplication analyses and ancestral genome reconstruction

### S2.7.1 Synteny among the Solanaceae

The CoGe platform for comparative genomics^91^ was used to detect orthologous genes between the berry-producing species *S. melongena,* *S. lycopersicum*, and *C. annuum* as well as *S. tuberosum* (**Figure 2B**). To compute chains of syntenic genes found within complete genome sequences the DAGchainer software^92^ (with ‘Relative gene order’ option and maximum distance between two matches parameter set to 5) was used together with Quota-Align algorithm^93^ (with maximum distance between two blocks sets to 4 genes), both implemented to the SynMap^91^ function within CoGe. To reduce background noise due to successive duplication(s), Quota-Align ratios for coverage depth was set to 2:2. The rates of synonymous substitutions (Ks-values) was calculated using CodeML of the PAML package^53^ implemented in SynMap^91^. Finally, a tandem duplication distance of 10 and a C-score (to filter low quality blast hits) of 0.40 were used. 492 syntenic blocks (14,206 genes) were identified between eggplant (~366Mb) and tomato (~199Mb), 645 (involving 13,807 genes) between eggplant (~344Mb) and potato (~220Mb) and 589 (9,227 genes) between eggplant (~174Mb) and pepper (539Mb) (**Supplementary Table S24**).

The synteny among the Solanaceae species highlighted the occurrence of 38 translocations between eggplant (~28Mb) and tomato (~12,6Mb), 176 translocations between eggplant (~102Mb) and potato (~49Mb) and 262 translocations between eggplant (~88Mb) and pepper (~288Mb). Furthermore, 213 inversions between eggplant (~147 Mb) and tomato (~81Mb), 329 between eggplant (~164Mb) and potato (~106Mb), and 263 between eggplant (~71Mb) and pepper (~211Mb) were also identified.

Ks was used to estimate the divergence time between eggplant with tomato, potato and pepper. Divergence times (T) were calculated using a synonymous mutation rate of 6.1 × 10^−9^ substitutions per synonymous site per year as T = Ks/(2 × 6.1 × 10^−9^)^94^. The obtained results suggested that the ancestral divergence between eggplant and pepper occurred around 20 mya, while the divergence between eggplant with both tomato and potato was dated between 14.0-14.7 mya.

The same COGE^91^ procedure previously described was used to detect paralogous genes in *C. annuum*, *S. lycopersicum, S. melongena* and *S. tuberosum* as well as orthologous genes in the four sequenced Solanaceae (eggplant, pepper, tomato and potato) with *C. canephora* and *V. vinifera*. To reduce background noise, due to successive duplication(s), Quota-Align^93^ ratios for coverage depth was set to 2:2 for orthology analyses, and to 1:3 for Grape/Coffee Vs. Solanaceae analyses.

From the eggplant side, the orthology analyses identified 434 syntenic blocks (5277 genes) between eggplant and grape and 432 (6,594) between eggplant and coffee. Divergence time of the Solanaceae species based on Ks estimation was found to be between 110-114 mya for grape and 100-114 mya for coffee respectively. These findings are in line with what previously reported^25^ for tomato, while Wikstrom et al.^95^ reported a more recent divergence between Solanaceae and Coffee (89 mya).

Orthologous analyses in the Solanaceae family allowed to date a triplication event (“T” event) at ~45-55 mya, accordingly to what previously reported^24,25^. The “T” triplication is also evident on the basis of *S. melongena-S. melongena, S. lycopersicum-S. lycopersicum, S. tuberosum-S. tuberosum, C. annuum-C. annuum, S. melongena-C. canephora and S. melongena-V. Vinifera* comparisons. The orthologous analyses also revealed that single genes on the grape and coffee chromosomes mapped to three genes in eggplant genome.

The eggplant-eggplant comparison revealed a total of 3,234 ohnologs (paralogous genes that were generated by the whole genome triplication event^96^) (**Supplementary Table S25, Figure 1**) of which just a fraction is still triplicated, while the majority underwent loss of one or two copies after the “T” event ^23–25^. These findings were confirmed by the identification of 5,099 tomato, 4,659 potato and 2,163 pepper ohnologs (**Supplementary Table S26)**. For each pair of ohnologs, KaKs_Calculator^97^ was used to estimate rates of synonymous substitutions (Ks-values).

The eggplant, tomato, potato and pepper ohnologs were analyzed for GO term enrichment using AGRIGO^89^. For eggplant, in the ontology class ‘biological process’ the top 3 enriched terms (base on p-value) were GO:0010556 (regulation of macromolecule biosynthetic process), GO:0045449 (regulation of transcription) and GO:0019219 (regulation of nucleobase, nucleoside, nucleotide and nucleic acid metabolic process. For ‘molecular function’ the top 3 enriched terms (base on p-value) were GO:0003700 (transcription factor activity), GO:0030528 (transcription regulator activity) and GO:0043565 (sequence-specific DNA binding). For tomato, in the ontology class ‘biological process’ the top 3 enriched terms (base on p-value) were GO:0009889 (regulation of biosynthetic process), GO:0045449 (regulation of transcription) and GO:0010556 (regulation of macromolecule biosynthetic process). For ‘molecular function’ the top 3 enriched terms (base on p-value) were GO:0003700 (transcription factor activity), GO:0030528 (transcription regulator activity) and GO:0043565 (sequence-specific DNA binding). For potato, in the ontology class ‘biological process’ the top 3 enriched terms (base on p-value) were GO:0010556 (regulation of macromolecule biosynthetic process), GO:0045449 (regulation of transcription) and GO:0009889 (regulation of biosynthetic process), while for ‘molecular function’ the top 3 enriched terms (base on p-value) were GO:0003700 (transcription factor activity), GO:0030528 (transcription regulator activity) and GO:0043565 (sequence-specific DNA binding). Finally, for pepper, it was found that in the ontology class ‘biological process’ the top 3 enriched terms (base on p-value) were GO:0009058 (biosynthetic process), GO:0050789 (regulation of biological process) and GO:0050794 (regulation of cellular process), whereas in ‘molecular function’ the top 3 enriched terms (based on p-value) were GO:0043565 (sequence-specific DNA binding), GO:0003700 (transcription factor activity) and GO:0050136 (NADH dehydrogenase (quinone) activity).

AGRIGO cross comparison of SEA (SEACOMPARE) was used to identify common and different enrichment GO terms between the four Solanaceae (**Supplementary Table S27**). A total of 28 biological process GO terms were shared among eggplant, pepper, tomato and potato, including the significantly enriched GO:0010556 (regulation of macromolecule biosynthetic process) and GO:0045449 (regulation of transcription). A total of 6 molecular function GO terms were shared among eggplant, pepper, tomato and potato, with GO:0003700 (transcription factor activity) and GO:0030528 (transcription regulator activity) being significantly enriched. It is well established that genome fractionation (elimination of duplicated gene redundancy^98,99^) follows a polyploidization event. The fate of different protein-coding gene familes varies during genome fractionation. Transcription factors (TFs) or regulators (TRs), are mainly retained following a polyploidization event and thus not negatively selected during evolution^100,101^. On the contrary, other genes are highly fractionated and return to single copy^100–103^. Pont et al.^103^ suggested that transcription factors are putatively involved in the response to rapidly changing biotic and abiotic factors compared with genes involved in relatively more stable processes, thus providing a selective advantage during evolution and adaptation. Some enriched ohnologs of eggplant, pepper, potato and tomato belong to the same gene ontology terms (transcription factor activity), confirming this evolutionary mechanism following the ‘T’ triplication occurred in the Solanaceae family.

### S2.7.2 Ancestral Solanaceae genome reconstruction

Wu and Tanksley^104^ first tried to reconstruct the ancestral chromosomal composition of Solanaceae. Their approach was based on the use of COSII markers, the use of *Nicotiana* as an outgroup, and the assumption that the Solanaceae have evolved largely in the absence of polyploidization. Thanks to the availability of anchored genomes for eggplant, pepper, tomato, potato and coffee, as well as the well-established genome triplication in the Solanaceae family firstly detected in tomato and potato^25^ and confirmed in eggplant and pepper (this paper), an identification of contiguous ancestral regions (CARs^106^) in the Solanaceae species was carried out.

The hypothetical ancestral chromosomes of the common ancestor of pepper, tomato, potato and eggplant was based on shared genes obtained from COGE^91^ outputs, between the four species as well as coffee, which was chosen as an outgroup. All the duplicated/triplicated matches (in the same species) obtained from COGE syntenic analysis were removed and a whole of 4,465 common unique genes between the five species was used for CARs identification. GRIMM-Synteny^106^ was used to identify common syntenic blocks shared between the 5 species with the following parameters: minimum block size = 6 units (in permutation metric) and maximum gap threshold per-species = 7 units (in permutation metric). The syntenic blocks identified were analyzed with two different pipelines: MGRA^107^ and ProCARs^108^ in order to compare and confirm the putative identified ancestral chromosomes. MGRA^107^ allows to detect 12 ancestral chromosomes, while ProCARs^108^ detected almost the same ancestral CARs but with a higher granularity (which means that some MGRA CARs were split in several small CARs by ProCARs). As the target was the identification of the 12 putative CARs at pepper divergence level, the MGRA output was used.

An overview of the CARs obtained, compared to the chromosomes of the four Solanaceae species is reported in **Supplementary Figure S5** and **Supplementary Table S28**.

CAR A1 corresponds to the majority of CH1 in eggplant, potato and tomato, while in pepper is syntenic to both CH1 and CH8 that are syntenic to A1, as previously reported by Wu and Tanksley^104^.

CAR A2 corresponds to CH2 of the four Solanaceae in this study

CAR A3 mainly matches to current pepper, tomato potato and eggplant CH3, but some translocations were found (a portion of A3 is currently on CH12 of eggplant, tomato and potato, and on CH9 in pepper, suggesting a different translocation event occurred only in the pepper lineage).

CAR A4 was not involved in relevant translocation events in the last 25 mya, as it corresponds to CH4 in all the species (just a small translocation occurred in eggplant on CH11 and pepper on CH12).

CAR A5 corresponds to CH12 in tomato, potato and pepper and to CH5 in *S. melongena,* thus the latter appears to have followed a different evolution pattern.

CAR A6 shows a ‘semi-conservative’ evolution pattern as it originated CH6 and CH9 in all the four species. However, it also originated CH7 in eggplant and CH3 in pepper in which other two specific translocations, on CH1 and CH5, occurred.

CAR A7 was highly conserved in all the four species and, with the exception of a small translocation (current eggplant CH4 or tomato/potato/pepper CH10), it corresponds to CH7 in all four Solanaceae.

CAR A8 corresponds to both CH1 and CH8 in the four Solanaceae. Most of the CAR8 correspond to CH1 in pepper, while in eggplant, tomato and potato it mainly corresponds to CH8, confirming that in pepper a specific evolution on CH1 and 8 occurred after its speciation^104^.

CAR A9 corresponds to the current CH9 in pepper, eggplant, tomato and potato.

CAR A10 is highly conserved in the four Solanaceae species. The only exception is eggplant, in which a portion of A10 translocated to the current CH4.

CAR A11 shows a ‘semi-conservative’ evolution pattern: it originated CH11 in all the four species as well as a portion of CH12 in eggplant and pepper.

The same procedure (MGRA) allowed to estimate the ancestral eggplant-potato-tomato (genus *Solanum*) genome and the ancestral tomato-potato (subgenus *Potatoe*) genome. For the former, the CARs obtained, compared to the chromosomes of the three *Solanum* species are reported in **Supplementary Table S28**.

CAR A1 correspond to CH1 in eggplant, potato and tomato.

CAR A2 corresponds to CH2 in the three *Solanum* species.

CAR A3 mainly corresponds to the current tomato, potato and eggplant CH3, however some translocations were identified (a portion of A3 is currently on CH12 of eggplant, tomato and potato).

CAR A4 was not involved in significant translocation events in the last 20 mya, as it corresponds to CH4 in all the species (just a small translocation was highlighted in eggplant on CH11).

CAR A5 evolved independently in eggplant, as it corresponds to CH12 in tomato and potato, while to CH5 in *S. melongena*.

CAR A6 originates CH6 in all the three species.

CAR A7 was highly conserved and corresponds to the present CH7 in all the three Solanaceae with the exception of a small translocation on eggplant CH10 and on tomato/potato CH12)

CAR A8 corresponds to both CH1 and CH8 in the *Solanum* genus*.*

CAR A9 shows no species-specific evolution during the *Solanum* history, as it corresponds to CH9 in tomato, potato and eggplant (in which a small lineage specific translocation of CH7 was also found).

CAR A10 is broadly conserved in all the three species, although a portion of A10 translocated to current CH4 in eggplant.

CAR A11 shows a ‘semi-conservative’ evolution pattern: it originated CH11 in all the three species but also a small portion of CH12 in eggplant and CH5 in potato.

CAR A12 substantially corresponds to CH5 of the three *Solanum* species, although portions of A12 translocated to current CH3 and CH10 in eggplant.

At the tomato-potato divergence level, the CARs obtained, compared to the chromosomes of the two species, are reported **Supplementary Table S28.**

CAR A1 correspond to CH1 in potato and tomato.

CAR A2 corresponds to CH2 in both Solanum species.

CAR A3 mainly correspond to current tomato and potato CH3, although, in both species, a small translocation was found on CH12.

CAR A4 was not involved in important translocation events in the last 20mya, as it corresponds to CH4 in all the species.

CAR A5 correspond to CH12 in both tomato and potato.

CAR A6 originates CH6 in all the two species.

CAR A7 reflects the current CH7 in potato and tomato.

CAR A8 corresponds to both CH1 and CH8 in the two species.

CAR A9 reflects the current tomato and potato CH9.

CAR A10 is the current potato and tomato CH10.

CAR A11 shows a ‘semi-conservative’ evolution pattern: it originated CH11 in all the two species and a portion of CH5 in potato as well, suggesting a lineage-specific translocation.

Finally, CAR A12 corresponds to CH5 in both species.

### S2.7.3 Ancestral genome evolution

During Solanaceae evolution, the three hypothesized ancestor genomes (Solanaceae*, Solanum and Potatoe*) faced a minimum of 6 translocations and 15 inversions from the *Solanaceae-Solanum* journey (average of 0.85 translocation and 2.1 inversions per mya) and at least 4 translocations and 35 inversions (average of 0.57 translocations and 5 inversions per mya) from the *Solanum* to the *Potatoe* ancestor. By using the Solanaceae ancestor genome as reference, it appears that some chromosomes within the ancestor genomes did not undergo any significant translocations (**Figure 2C)**. This is the case of CAR A4, A7, A11 and A12, which mainly correspond to the same chromosomes in different Solanaceae species at any divergence levels. On the contrary, other ancestral chromosomes rearranged extensively during ancestor evolution. As an example, CAR A9 in *Solanum* as well as in *Potatoe* contains segments of CH6 and CH9 of the common Solanaceae ancestor and underwent at least 5 inversions in the transition from the Solanaceae to the *Solanum* ancestor, and 3 additional inversions in the transition from the latter to the *Potatoe* ancestor.

### S2.7.4 Species-specific ancestor genome evolution

Pepper experienced at least 54 translocations and 71 inversions after its divergence from the Solanaceae ancestor, while eggplant faced a minimum of 18 translocations and 50 inversions during its speciation from the *Solanum* ancestor. On the other hand, tomato and potato underwent respectively to at least 2 and 3 translocations as well as 21 and 42 inversions during their evolution from their common *Potatoe* ancestor (**Figure 2C)**.

With respect to the *Solanaceae* ancestor genome, some extensive rearrangements were spotted during speciation. A peculiar example is the CH3, which in tomato and potato contains only segments of CAR A3 while in pepper and eggplant consists of segments from both A3 and A6 and from A3 and A12 respectively. Other lineage-specific translocation events occurred in eggplant and pepper, in both of which translocations of CAR A11 to CH12 were detected. In pepper, both CH1 and CH8, include CAR A1 and A8 regions: this confirms the previously detected illegitimate pairing and crossing over event between two non-homologous, metacentric chromosomes^21,101^. However, in CH1 of potato, tomato and eggplant a previously unreported small translocation of the ancestral Solanaceae A8 was also detected. This implies that the four species faced, with different magnitude, an A8 translocation to CH1, while the translocation of A1 to CH8 seems exclusive of *C. annuum*. Finally, some translocations occurred side-by-side in potato and tomato after their separation from the common ancestor (see CAR A3 or A8).

# S3 Targeted gene family analyses

## **S3.1 Disease resistance response genes**

Over 950 pathogen receptor genes (PRGs), were annotated in the eggplant genome using BLASTp search and InterPro domain composition scanning. They represent approximately 2,5 % of the total eggplant gene complement, i.e. more than in tomato (2%) but less than in potato and pepper (3.6% and 4.4% respectively). Most of them belong to the main resistance protein classes and were annotated as full-length genes. In particular, 131 sequences were assigned to CC-NB-LRR (CNL), 36 to TIR-NB-LRR (TNL), 46 to Receptor Like Proteins (RLP) and 261 to Receptor Like Kinase (RLK) classes. Proteins composed by incomplete PRG-domain arrangement (partial classes) were also found and annotated (**Supplementary Table S29)**. The phylogenetic relationships of Solanaceae CNL and TNL proteins were inferred separately by comparing our dataset with fifty-four well-characterized reference *CNL* genes and fifteen *TNL* genes, respectively. A maximum likelihood analysis based on the Jones et al.^109^ model was carried out on 937-CNL and 128-TNL Solanaceae proteins (full-length genes) together with 54-*CNL* and 15-*TNL* plant reference *R*-genes, and the model with the lowest BIC score (Bayesian Information Criterion) was considered to describe the substitution pattern the best (**Supplementary Figure S6**). Sequence similarities were determined performing a MAFFT^110^ (E-INS-i algorithm) multiple alignment. Clades were collapsed and numerated based on a bootstrap value over 85. Evolutionary analyses were conducted in MEGA6^111^.

TNL members located on eggplant CH5 and 11 were subject to duplication (Expansions 1 and 2 in **Supplementary Figure S6)** and on CH9 of potato (Expansion 3 in **Supplementary Figure S6**). In previous studies, copy number expansions/contractions at important Solanaceae *R*-gene loci, involved in *Phytophthora infestans* and *Fusarium oxysporum* f. sp. *lycopersici* resistance, were evidenced ^112–114^. Such findings suggest that adaptive diversification is induced by species-specific pathogen pressure. In general, larger clusters are generated for promoting diversification of main resistance loci in a given species^115^. Solanaceae *R*-loci enlargement (i.e. I2, R2 and Mi1.2 loci) and diversifications were identified in common chromosome regions (**Figure 6B**), suggesting that the genome location of genes, that exert resistance function, is not established by chance^116^.

## S3.2 Steroidal glycoalkaloids

Steroidal glycoalkaloids (SGAs) are nitrogen-containing secondary metabolites produced by numerous members of the Solanaceae family. Some examples of these compounds are α-tomatine and dehydrotomatine in tomato, α-chaconine and α-solanine in potato, and studied to lesser extent, solamargine and α-solasonine in eggplant. Another important Solanaceae food crop, pepper (*Capsicum annuum*), is well known for capsicosides (steroidal glycosides) and their derivatives, steroidal saponins and capsaicinoids (e.g. capsaicin), the latter being a unique group of phenylpropanoid-derived secondary metabolites^117^, but there is hardly any information on steroidal glycoalkaloids produced in pepper. SGAs play a protective role in plants against a wide range of pathogens and predators, including bacteria, fungi, oomycetes, viruses, insects and animals^118^. Moreover, the anti-carcinogenic potential of various *Solanum* alkaloids was tested against the development and progression stages of various cancers^119^. Despite these properties, some of the SGAs are considered as anti-nutritional compounds due to toxic effects to humans.

Cholesterol serves as a key precursor in SGA biosynthesis. Itkin et al.^120^ identified a set of *GLYCOALKALOID METABOLISM* (*GAME*) genes that partake in SGA biosynthesis in tomato and potato. Furthermore, a detailed pathway for SGA biosynthesis starting from cholesterol to SGAs was suggested in Solanaceae plants. Based on comparative co-expression analysis, GAME genes participating in SGA biosynthesis were found to be positioned closely in the tomato and potato genomes, forming a metabolic gene cluster. In tomato (*GAME1*, *GAME2*, *GAME6*, *GAME11*, *GAME17*, *GAME18*) and potato (*SGT1*, *GAME6*, *GAME11* and *SGT3*) *GAME* genes involved in SGAs biosynthesis form a major cluster on CH7, with a further two *GAME* genes (*GAME4* and *GAME12*) next to each other on CH12 (**Figure 6C**). We further examined the eggplant genome for the presence of a metabolic gene cluster similar to that of tomato and potato. Four putative *GAME* genes (*GAME1*, *GAME2*, *GAME6*, *GAME11*) were found in eggplant that are organized in a form of a metabolic gene cluster on CH7 and two *GAME* genes (*GAME4* and *GAME12*) located next to each other on CH12 (**Figure 6C**). Thus, the genes for synthesis of SGAs α-solasonine and solamargine are likely positioned on eggplant CH7 and 12, in regions that are syntenic to those in which the SGAs metabolic gene clusters are located in tomato and potato. In the case of pepper, five putative *GAME* genes (*GAME1*, *GAME6*, *GAME11*, *GAME17* and *GAME18*) were positioned as cluster on CH7, while the cluster on CH12 was not detected (**Figure 6C**). **Supplementary Table S30** displays the putative eggplant orthologs (best hits) involved in glycoalkaloid biosynthesis.

A typical SGA contains two structural components; the aglycone, cholesterol derived skeleton containing nitrogen, and a carbohydrate side chain generally comprising of various combinations of glucose, galactose, xylose and rhamnose. Among the clustered genes in tomato and potato, GAME6 (a cytochrome P450) and GAME11 (a dioxygenase) are involved in the hydroxylation of cholesterol intermediates (e.g. 22, 26-dihydroxycholesterol) and the generation of a furostanol type aglycone^120^. This furostanol aglycone is further oxidized and transaminated by GAME4 and GAME12 respectively to produce the nitrogen containing steroidal alkaloid (SA) aglycone. Formation of the aglycone seems to be a common step in eggplant as supported by presence of GAME6, GAME11, GAME4 and GAME12 in these species (**Figure 6C** and **Supplementary Figure S7**).

The furostanol type aglycone also serves as a branching point for the formation of steroidal saponins (e.g. uttroside B) as observed in *GAME4*-silenced tomato/potato plants^120^. This explains the case of pepper in which GAME6 and GAME11 are present to likely generate a furostanol type aglycone which is further converted to steroidal saponins (e.g. capsicosides) and the absence of SGAs due to lack of GAME4 and GAME12 required for transamination.

In tomato and potato, the SA aglycone is decorated by various UDP-glycosyl transferases (UGTs) to generate the glycosylated forms of steroidal alkaloids. In tomato, α-tomatine and dehydrotomatine contain a lycotetrose moiety composed of galactose, glucose, glucose and xylose units that are likely added to the SA aglycone by GAME1, GAME17, GAME18 and GAME2, respectively. In potato, α-solanine and α-chaconine are generated by adding solatriose (galactose, glucose and rhamnose) and chacotriose (glucose, rhamnose, rhamnose), respectively. SA glycosylation is performed by SGT1 (galactosyltransferase), SGT2 (glucosyltransferase) and SGT3 (rhamnosyltransferase). In eggplant, α-solasonine and solamargine are glycosylated by solatriose and chacotriose, respectively (as the major potato SGAs). Phylogenetic analysis of GT family members demonstrated the presence of putative GAME1 (SGT1 or GAME1 ortholog), SGT2 (two putative homologs) and SGT3 enzymes in eggplant, forming a distinct clade with respective to the potato and tomato GTs catalyzing the glycosylation of SAs (**Supplementary Figure S8**). In the eggplant genome, we also found closest homologs for GAME17 and GAME18, two glucosyltransferases specifically catalyzing terminal glycosylation reactions of SAs in tomato. Among these GTs, SGT1 and SGT3 in eggplant are present on CH7 and are part of SGA metabolic cluster.

## S3.3 Fruit ripening regulators and coexpression analyses

Comparative expression of the genes encoding major ripening regulators: *RIPENING INHIBITOR (RIN), FRUITFULL1* and *2 (FUL1-2), NON RIPENING (NOR), COLORLESS NON-RIPENING (CNR)*^121^*, TOMATO AGAMOUS-LIKE 1 (TAGL1), AUXIN RESPONSE FACTOR 2A (ARF2A)* and *APETALA2a (AP2a)*^122^ was studied in different tissues of tomato, eggplant and pepper. With the exception of *CNR*, these genes showed ripening-associated expression in all three species, while *CNR* showed ripening-associated expression only in tomato (**Figure 4A**). The function of the MADS box gene, *TOMATO AGAMOUS-LIKE 1 (TAGL1)*^123^ is conserved in tomato and eggplant.

Co-expression analysis was carried out with CoExpNetViz^124^ using the tomato MADS-box encoding gene RIN and its closest ohnolog in eggplant and pepper were used as 'baits'. The known sequence of *CaRIN*^125^ was split by the annotation into two genes, *CA11g03200* and *CA11g03210*. For the purpose of the co-expression analysis, the expression values of *CA11g03200* and *CA11g03210* were summed and the two genes were treated as one. The analysis resulted in lists of co-expressed genes (r-value ≥ 0.6) (**Supplementary Table S31**). Gene names are color coded according to species co-expression.

## **S3.4** Pigment biosynthesis

### **S3.4.1 Phenylpropanoid biosynthesis**

In Solanaceae, pigments are accumulated in fruits early and late during ripening: chlorophyll and yellow xanthophylls in unripe (stage 2) tomato and pepper fruits, and lycopene and red or yellow xanthophylls in ripe (stage 3) tomatoes and red or yellow peppers, respectively. In eggplant, commercially ripe fruit accumulates anthocyanins in the epidermal cells that are responsible for its violet to black appearance, whereas carotenoids do not accumulate at high rates but rather are degraded in physiologically ripe fruits, contextually with a massive shift in the production of flavonols, similar to tomato and pepper, to the detriment of anthocyanins. A sequence similarity-based approach using Blast was applied employing genes or orthologs previously identified in eggplant or other species including tomato, Arabidopsis, grape and Petunia (**Supplementary Table S32**). The analysis resulted in the reconstruction of the whole process, including a number of anthocyanin regulatory genes already identified in allied species (**Supplementary Table S32).** Orthology with the other Solanaceae present in this study was based on synteny analyses with CoGe and, where not possible, by blast best-hit.

As expected, most of the anthocyanin-specific late biosynthetic genes LBGs^126^, starting from the *DIHYDROFLAVONOL 4-REDUCTASE* (*DFR*), were up-regulated in flower and young fruits and skin tissues at stage 1, with a marked decrease at stage 3 of fruit development (**Supplementary Figure S9**), in accordance with the down-regulation of the *ANTHOCYANIN1* (*ANT1*) and *JOHNANDFRANCESCA13* (*JAF13*) transcription factors thus, leading to the observed fruit phenotype at this stage which show a clear reduction of anthocyanin concentration^127^. Interestingly, a dramatic increase in expression was found in *FLAVONOL SYNTHASE* (*FLS*) in the skin of over-mature fruit, suggesting the existence of a metabolic shift from anthocyanin to flavonol production at stage 3 of ripening (**Figure 5A**). Indeed, it is well documented in other species that FLS and DFR enzymes compete for common substrates, dihydroflavonols, thus leading to flavonols and anthocyanins, respectively. This metabolic balance is strictly controlled by the activity of a R2R3-MYB factor, namely MYB12 which shows an expression profile very similar to that of FLS (**Supplementary Figure S9**), suggesting that this switch may be triggered by *SmMYB12* in eggplant, as already known in other species^128–131^. Interestingly, these genes are mainly expressed in the fruit epidermis, which is the main site of accumulation of both anthocyanin pigments and naringenin chalcone/flavonol co-pigments in the two species (Chappell-Maor et al., unpublished data). Such co-pigments accumulate mainly due to the high expression level of *CHALCONE SYNTHASE (CHS)* genes at stage 3 of development (**Supplementary Figure S9**). In tomato flowers and fruits at stage 2 and 3, flavonols are the major flavonoid derived compounds and are preferentially synthesized from dihydrokaempferol and dihydroquercetin by the action of F3H/F3’H and FLS; this correlates with high levels of *MYB12* and *FLS* expression observed at stages 2 and 3 of fruit ripening, in keeping with the high levels of flavonols synthesized in both stages. In eggplant flowers and fruits at stage 2, anthocyanin synthesis occurs via F3H/F3’5’H, while the flavonol pathway is expressed at basal level.

As a whole, in red tomato fruits flavonoid metabolism is directed towards the flavonol branch throughout fruit development, while in eggplant the metabolic balance between the anthocyanin and the flavonol branches changes during development. The accumulation of co-pigments, such as naringenin chalcone and flavonols at stage 3 of maturation (physiological ripeness), is in accordance with the peculiar fruit skin color transition from violet (stage 2, commercial ripeness) to yellow (**Figure 5A**).

Interestingly, an anthocyanin accumulating tomato line, Sunblack^122^, accumulates anthocyanins in fruit skin in a high light-dependent manner, due to the induction of *F3’5’H* and *DFR* and the down-regulation of *FLS* compared to the wild type Ailsa Craig; this suggests that under particular environmental conditions, the flavonoid pathway can be redirected towards different metabolites. In pepper, the *CHS4* gene is particularly induced in ripe fruits, suggesting that accumulation of naringenin chalcone should occur at this stage (**Supplementary Figure S9**).

Anthocyanin regulators are members of three different families namely R2R3-MYB, bHLH and WD40. In dicotyledonous species the early and late biosynthetic genes are controlled by different groups of regulatory factors. Indeed, the early biosynthetic genes (EBGs) are regulated at the transcriptional level by a set of redundant MYB TFs like the ortholog genes of *SlMYB1*2^130^, *AmMYB340* and *AmMYB305*^132^, while the expression of LBGs is under the control of a regulatory complex known as MYB-bHLH-WD40 (MBW) (**Supplementary Figure S9**) which is highly conserved among dicot species and is composed by at least one member of the three gene families. The spatial and temporal expression of the LBGs is determined by a combination of different families of TFs and by their interactions. In eggplant, most of the anthocyanin positive regulators share a marked decrease following fruit maturation; in particular, we have identified the eggplant orthologs of tomato *ANT1* and *AN2* (**Supplementary Table S32)** which are preferentially expressed during early fruit maturation stages and in flowers, in line with their regulatory role as R2R3-MYB encoding genes and with the transcriptional profile of *DFR*. Interestingly, due to their tissue-specificity, it can be hypothesized that different MBW complexes may act to promote anthocyanin synthesis by recruiting ANT1 and AN2 in the fruits and in flowers, respectively (**Supplementary Figure S9**). Moreover, the same cluster, which in tomato comprises 4 genes^133^, has only two genes in eggplant. We have also identified the eggplant orthologs of tomato AN1 and JAF13, two bHLH factors known to be involved in the MBW complex^133^. Interestingly, the profile of both *SmAN1* and *SmJAF13* resembles the one of *SmANT1* and *SmAN2*, as well as of *DFR* and *ANS*, thus corroborating the hypothesis that they play a role in the MBW regulatory complex. Differently from what has been shown in tomato^133^, the eggplant ortholog of *SlJAF13* was induced in the fruit skin at stage 1 and in flowers, thus suggesting its involvement in the anthocyanin regulatory complex in these two organs. In a previous study, we characterized the expression of some of these regulators through qRT-PCR in several eggplant tissues demonstrating that *SmANT1*, *SmAN2* and *SmAN1* are predominantly expressed where anthocyanins synthesis occurs. Moreover, we showed that the transient expression of *SmANT1* in tobacco leaves leads to anthocyanin synthesis^134^. Conversely, *SmAN11* shows a constitutive expression in both eggplant and tomato, in keeping with its role as a scaffold protein^132,134,135^.

### S3.4.2 Carotenoid biosynthesis

Carotenoids are present in the eggplant fruit at a concentration much lower than that found in tomato and pepper^136^. All biosynthetic genes could be identified in eggplant (**Supplementary Table S33) a**nd comparative examination evidenced a good degree of conservation of gene numbers encoding different biosynthetic steps; a similar situation was evidenced for carotenoid regulators (**Supplementary Table S33)** The schematic pathway is depicted in **Figure 5A**, while the expression profiles of structural and regulatory genes in the pathway are depicted in **Figures S10**.

Comparative expression profiling highlights the different regulatory strategies in fruits of the three plants: in tomato, two early genes involved in lycopene biosynthesis, *PSY1* and *ZISO*, are strongly induced during fruit ripening, while two lycopene cyclization genes, *LYCB2* and *CYCB,* are repressed, resulting in the accumulation of lycopene. In pepper, the induction of *PSY1* is accompanied by that of *CYCB/CCS* and *CHY2* and by sustained expression of beta-cyclases, resulting in the accumulation of hydroxylated beta-xanthophylls and of their derivatives, capsanthin and capsorubin. In eggplant, most genes show low, albeit detectable expression, in ripening fruits. This is an indication that the biosynthetic pathway is active, and that the low levels of xanthophyll pigments accumulated are due either to the unavailability of carotenoid-sequestering structures or to the action of carotenoid-degrading enzymes, similar to what observed in other fruits and flowers. The low expression of *PSY1* confirms that this is the main rate-limiting step in carotenoid biosynthesis in Solanaceous fruits^137^. Consistent to the second hypothesis, CAROTENOID CLEAVAGE DIOXYNENASE 4 (CCD4), which in several fruits converts xanthophylls into colorless apocarotenoids ^138,139^, is highly expressed throughout eggplant fruit ripening, while it is switched off in pepper and tomato fruits during late ripening. Several differences are found in the expression of pigment biosynthesis regulators in the three plants: for instance, the *SGR1* gene, whose mutations are responsible for the green flesh phenotype in tomato and pepper fruits^140^, is more expressed in these fruits than in those of eggplant, while *APRR-like 2*, shown by network analysis to be associated to carotenoid biosynthesis in tomato and pepper^141^ shows the opposite trend.

## S3.5 Cuticle biosynthesis

Cuticular waxes are a mixture of compounds such as long-chain fatty acids, alcohols, alkanes, esters or in some cases triterpenoids^142^. Cutin is defined as a polyester mainly formed by C16 and C18 hydroxy and hydroxy-epoxy fatty acid monomers while other compounds such as glycerol have also been suggested to be part of the cutin polymer^143^. The epidermis layer bordering the cuticle is the source of building blocks for its construction. Cutin monomers and waxes are transported from their sites of biosynthesis in the epidermis layer through the plasma membrane and cell to the extracellular matrix for cuticle assembly.

Based on a list of tomato genes previously associated with fruit cuticle assembly^144^ we have derived their eggplant orthologs as reciprocal best hits (**Supplementary Table S19)**. To corroborate their involvement in cuticle biosynthesis, we examined enrichment of gene expression in skin compared to flesh tissues at two ripening stages in both tomato and eggplant (**Supplementary Table S34-S35, Figure 5B**).

The number of genes enriched in eggplant skin developmental stages B and C were 1,862 and 1,723, respectively, with 412 genes enriched in both stages, while the number of genes enriched in tomato skin breaker and red ripening stages were 334 and 289, respectively, with 151 enriched in both stages. Orthologous eggplant cuticle genes have been mapped onto a scheme of cuticle assembly and skin-enriched genes (**Figure 5B**). The detection of skin associated genes in tomato and their orthologs in eggplant provided us with information on specific genes that are members of multi-gene families that are strong candidates to act in cuticle biosynthesis in eggplant. For example, we found six LONG CHAIN ACYL COA SYNTHESTASE (LACS) orthologs in eggplant but only a single one of them was skin enriched and moreover as its tomato counterpart. This specific *LACS* gene is a most promising candidate to attach a CoA moiety to the de novo synthesized C16/C18 fatty acids^145^. These activated fatty acids could either enter the fatty acid elongase (FAE) complex for further wax biosynthesis or alternatively be serving as precursor for cutin monomer formation. In the case of CER1, 5 homologs existed but only two showed skin-associated expression pointing their involvement in wax biosynthesis.

Interestingly, we found orthologous genes (based on **Supplementary Table S19**) that were enriched in eggplant skin only for a single protein partner out of the 4 FAE complex proteins (i.e. CER6^146^). In several cases, a single ortholog was found to be skin enriched in both eggplant and tomato suggesting it as an excellent future cuticle associated protein candidate in eggplant. This was the case for CYP86A4 and GPAT6 in the cutin biosynthesis pathway, FDH in wax metabolism as well as CUS1 (CUTIN SYNTHASE^147^) polymerizing cutin in the extracellular matrix.

In the past decade, several ATP-binding cassette (ABC)-type transporters, mainly in Arabidopsis, have been reported to act in the transport of cuticular lipids through the epidermis cell plasma membrane to the extracellular matrix^142,148^. ABCG11/AtWBC11/DSO and ABCG12/CER5 transporter proteins belong to the WHITE-BROWN COMPLEX (WBC) subfamily, members of which are half-transporter proteins that could function as homo or heterodimers (between each other). ABCG12/CER5 acts in cuticular wax transport, ABCG11/AtWBC11/DSO was reported to act in transport of wax and cutin monomers and a third transporter, ABCG13, likely acts in cutin transport in Arabidopsis petals^149^. A different, full size ABC transporter, PEC1/ABCG32, was associated with cutin transport^150^. Interestingly, while homologs of all these 4 proteins were found in the eggplant genome only one of them (a homolog of ABCG12/CER5) was enriched in the eggplant fruit skin tissues.

The analysis of skin associated genes in both fruit species revealed 3 different regulatory proteins that are highly likely involved in cuticle formation in eggplant. These included the homologs of HDG1/CD2, MIXTA and MYB30/96^151^. SHN proteins were associated with the regulation of cutin biosynthesis in both Arabidopsis and tomato^149,153^, yet, their homologs were not found enriched in the eggplant fruit skin tissues. This suggested that they might not play a significant role in cuticle formation in eggplant fruit. Alternatively, they might possess an additional regulatory function in the pericarp of eggplant fruit.

##
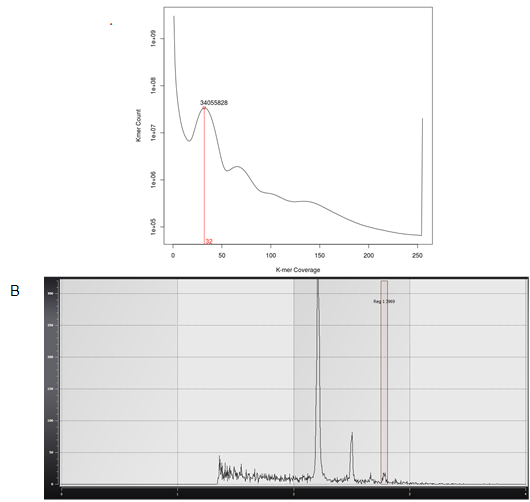
SUPPLEMENTARY FIGURES

**Supplementary Figure S1. a) K-mer frequency statistics**. Distribution of k-mer species according to the k-mer coverage depth in eggplant sequenced reads. Pair-End libraries with 400bp and 500bp insert size were used, 39,435,209,57623-mer were collected and frequency graph plotted. A peak of 23-mer frequency was reached at coverage depth of 32 with more than 34 million species, letting estimate a genome size of 1.04Gb. **b)** Flow cytometric histogram of eggplant nuclei DNA content. The four peaks correspond to 2x, 4x, 8x and 16x nuclei, as eggplant, like other plant species, undergoes to endo-reduplication events during cell and organ differentiation^154^. Standard control (highlighted in red) is *Ophiopogon planiscapus* 'Niger' (pg/2C 11.87). The 2C content was estimated at 2.49 pg, or 2.43 Gb. The analysis was performed by Plant Cytometry Services (Didam, The Netherlands).


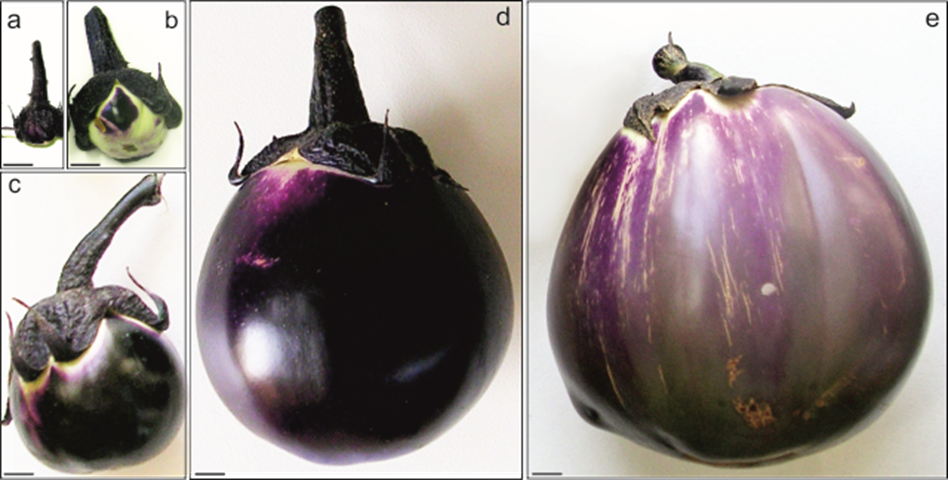


**Supplementary Figure S2. Eggplant fruits at 5 different developmental stages**: a) Fruits Ø 1 cm, at 3-4 days after flowering (DAF), with the fruit still almost fully covered by sepals; b) Fruits Ø 2-4 cm at 8-14 DAF (named stage 1); c) Fruits Ø 6 cm at 18-21 DAF; d) Fruits at commercial ripening (named stage 2) at approximately 38 DAF; e) Fruits at physiological ripening (named stage 3) at 55-60 DAF. Scale bar in each image represents 1cm.


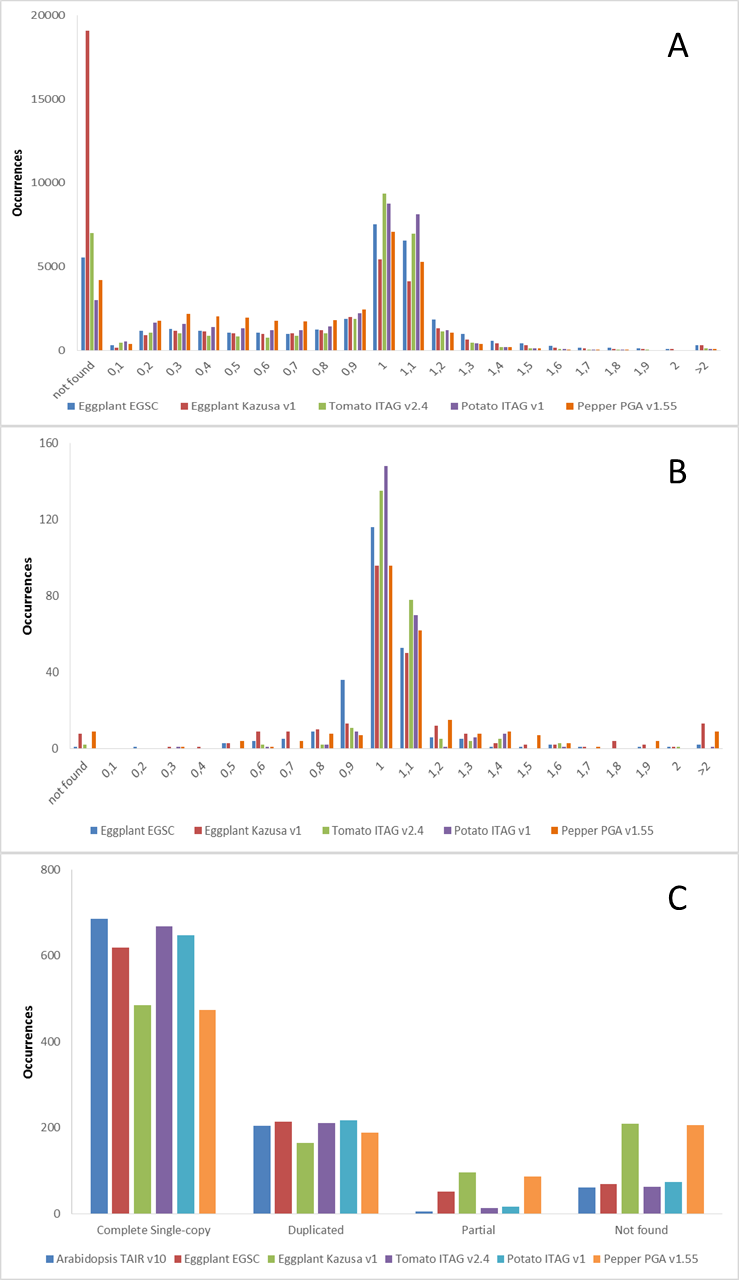
**Supplementary Figure S3. A**: Histogram of pairwise comparisons of predicted protein length ratios with their best hit in the Arabidopsis TAIR10^80^ annotation (BLASTP, e-value 10^-3^). **B**: Histogram of pairwise comparisons of predicted protein length ratios of the Arabidopsis 248 CEG proteins^78^ to their best hit in the various annotations (BLASTP, e-value 1e-3). **C**: Assessment of completeness metrics of the annotation with regard to the BUSCO^10^ gene sets (n= 1375).

**
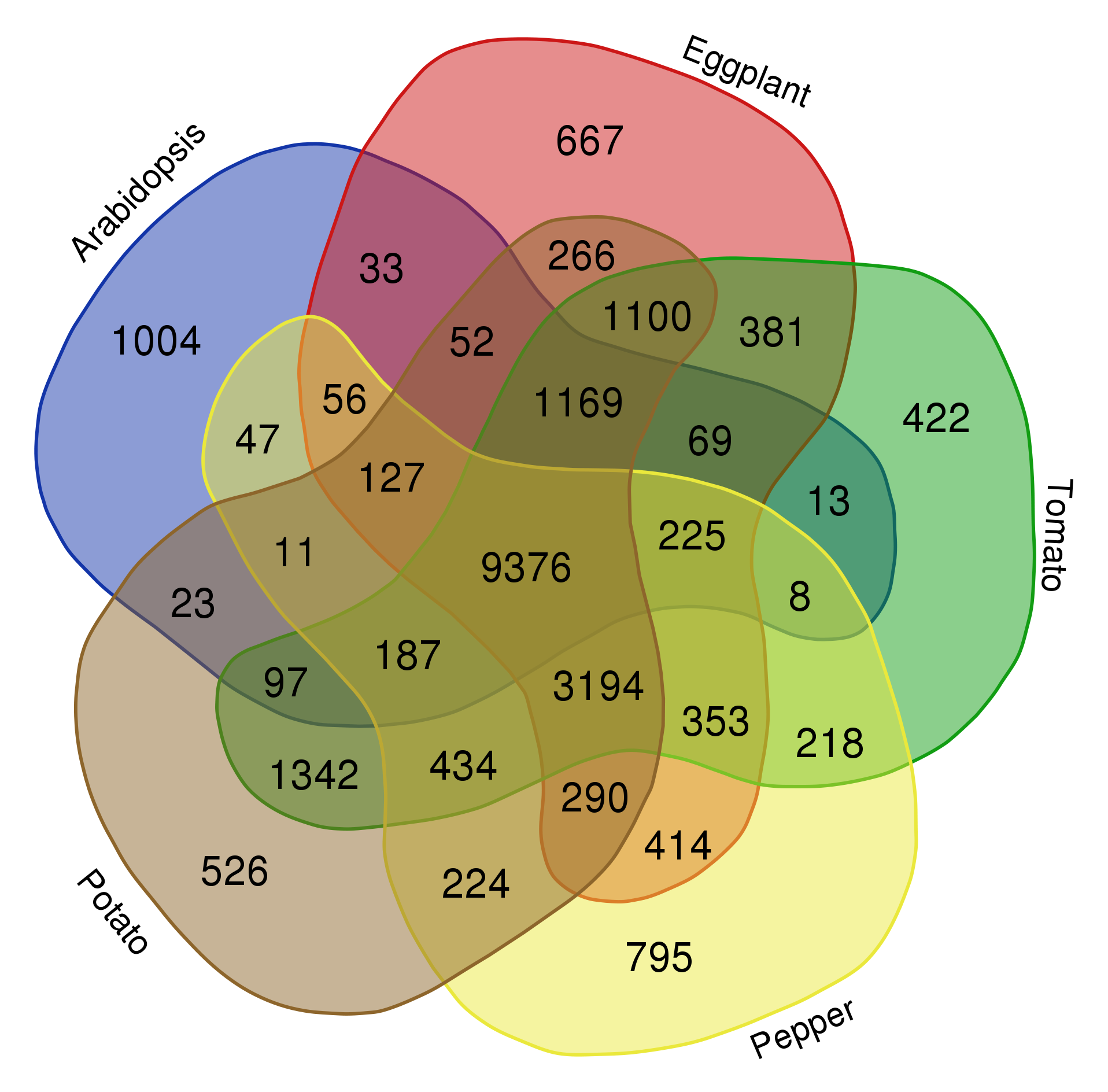
Supplementary Figure S4. Distribution of orthologous gene families in eggplant, tomato, potato, pepper and Arabidopsis, calculated with OrthoMCL**^81^


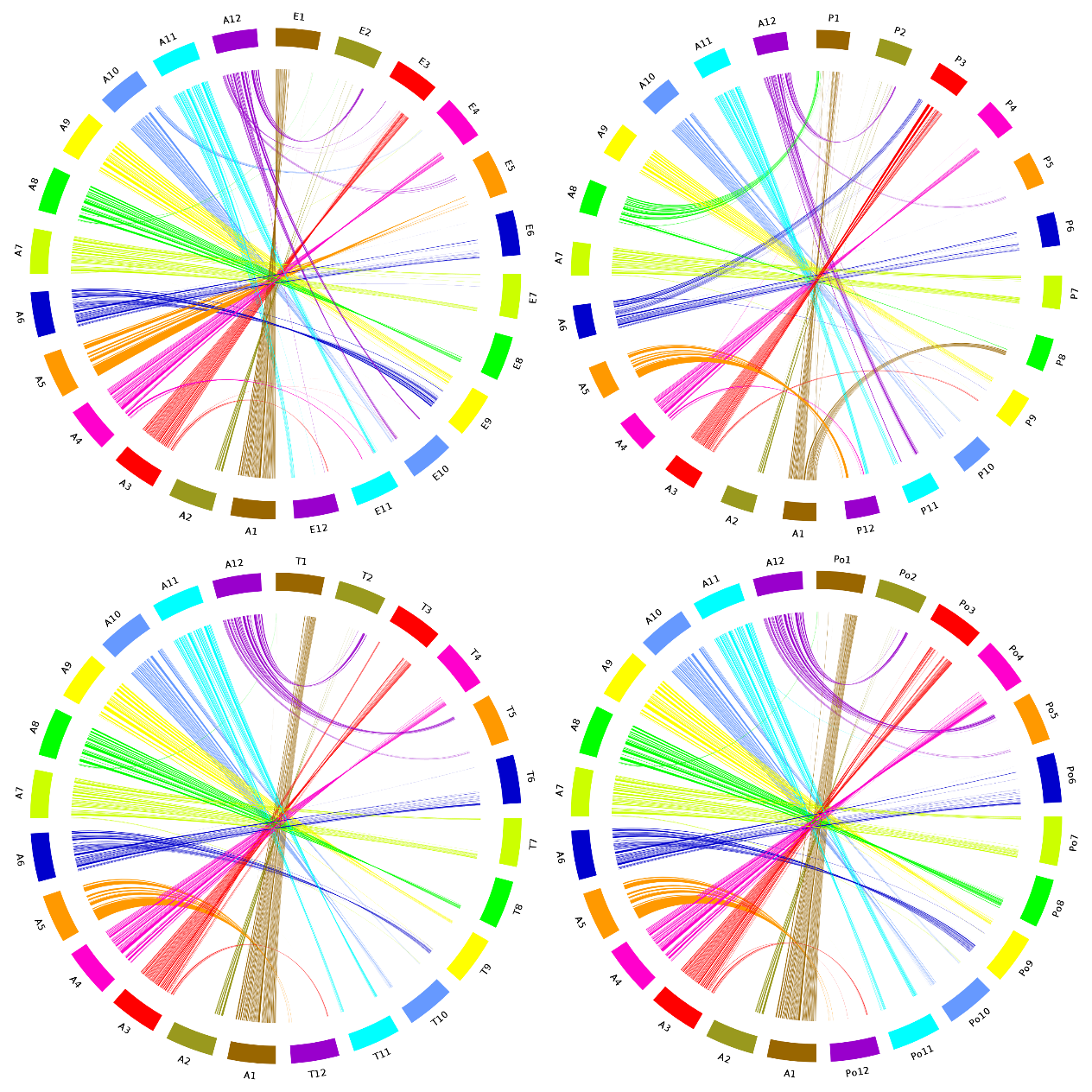
**Supplementary Figure S5.** **Ancestral Solanaceae chromosomes compared to eggplant (top left), pepper (top right), tomato (bottom left) and potato (bottom right).**


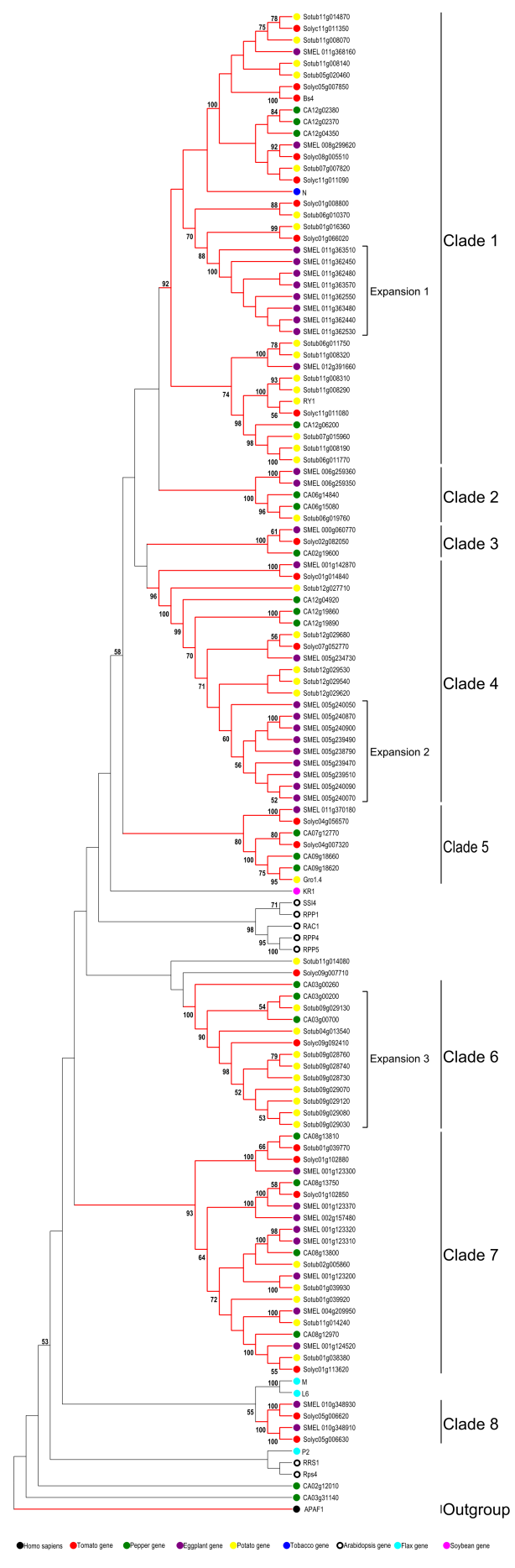


**Supplementary Figure S6.** **Evolutionary history of TNL full length genes in Solanaceae species (eggplant, tomato, potato and pepper).** Full NB-ARC domains of 128 TNL proteins (full-length genes) were used together with 15 well characterized plant reference R genes (short gene name) Two *S. melongena* ‘67/3’ TNL groups, including only members located on CH5 and 11 are indicated as subclade 1 and 2. Labels show the gene and the bootstrap values higher than 50 (out of 100), are indicated above the branches. The tree is drawn to scale, with branch lengths proportional to the number of substitutions per site. Species to which belong sequences are indicated by colored spots.


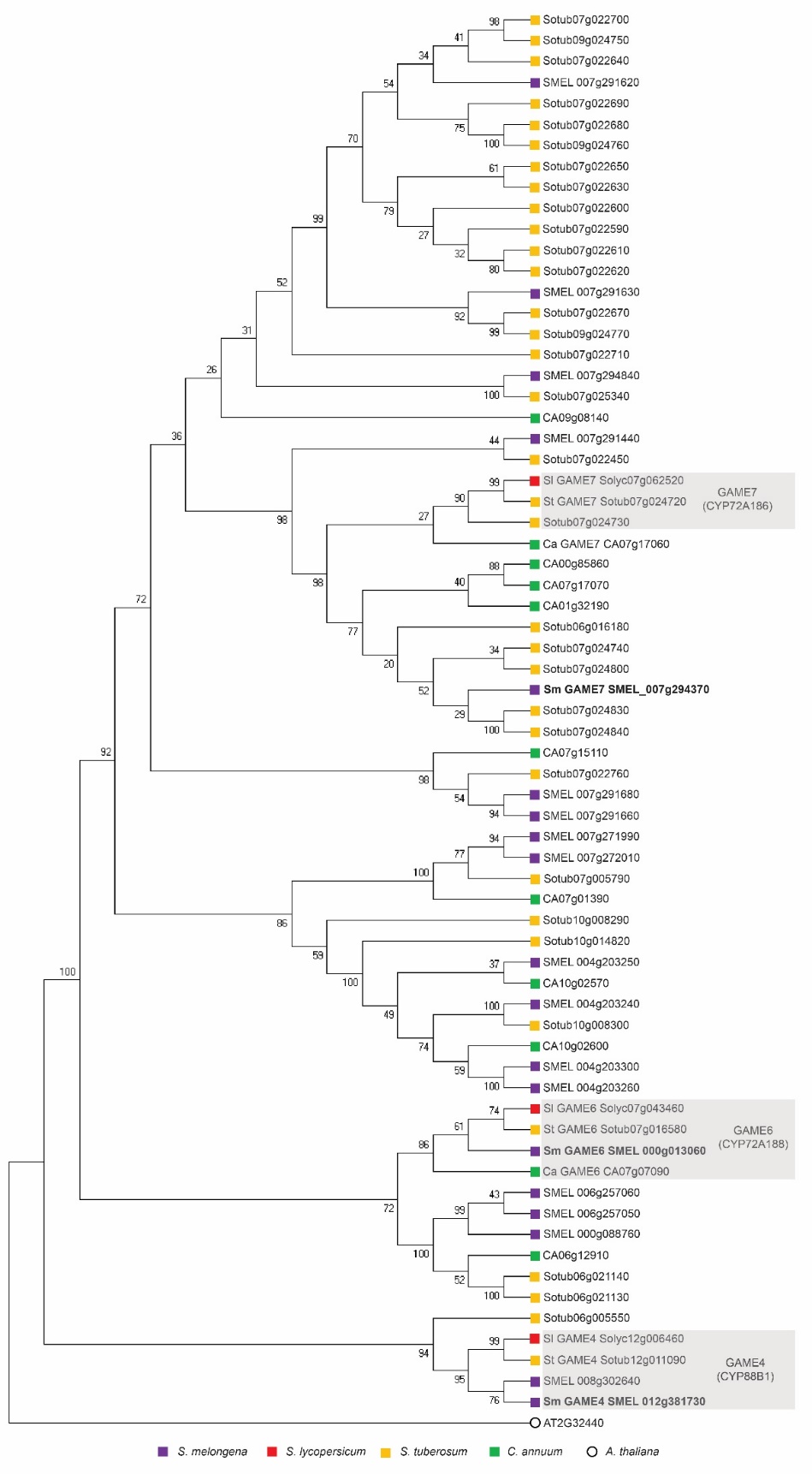
**Supplementary Figure S7. Phylogenetic tree of selected plant Cytochrome P450 enzymes in four Solanaceous plants**. Sequences from the following species were represented: tomato (Sl), potato (St), pepper (Ca), eggplant (Sm), and *Arabidopsis thaliana* (AT). Eggplant P450 enzymes participating in SGA biosynthesis are shown in square boxes. Protein sequences of known tomato Cytochrome P450 genes involved in SGA biosynthesis^116^ were aligned against eggplant, pepper, potato and Arabidopsis protein databases using BLASTP. For eggplant, pepper and potato, BLAST hits with an e-value lower than 4E-138 were selected. Arabidopsis best hit was taken as an outgroup.


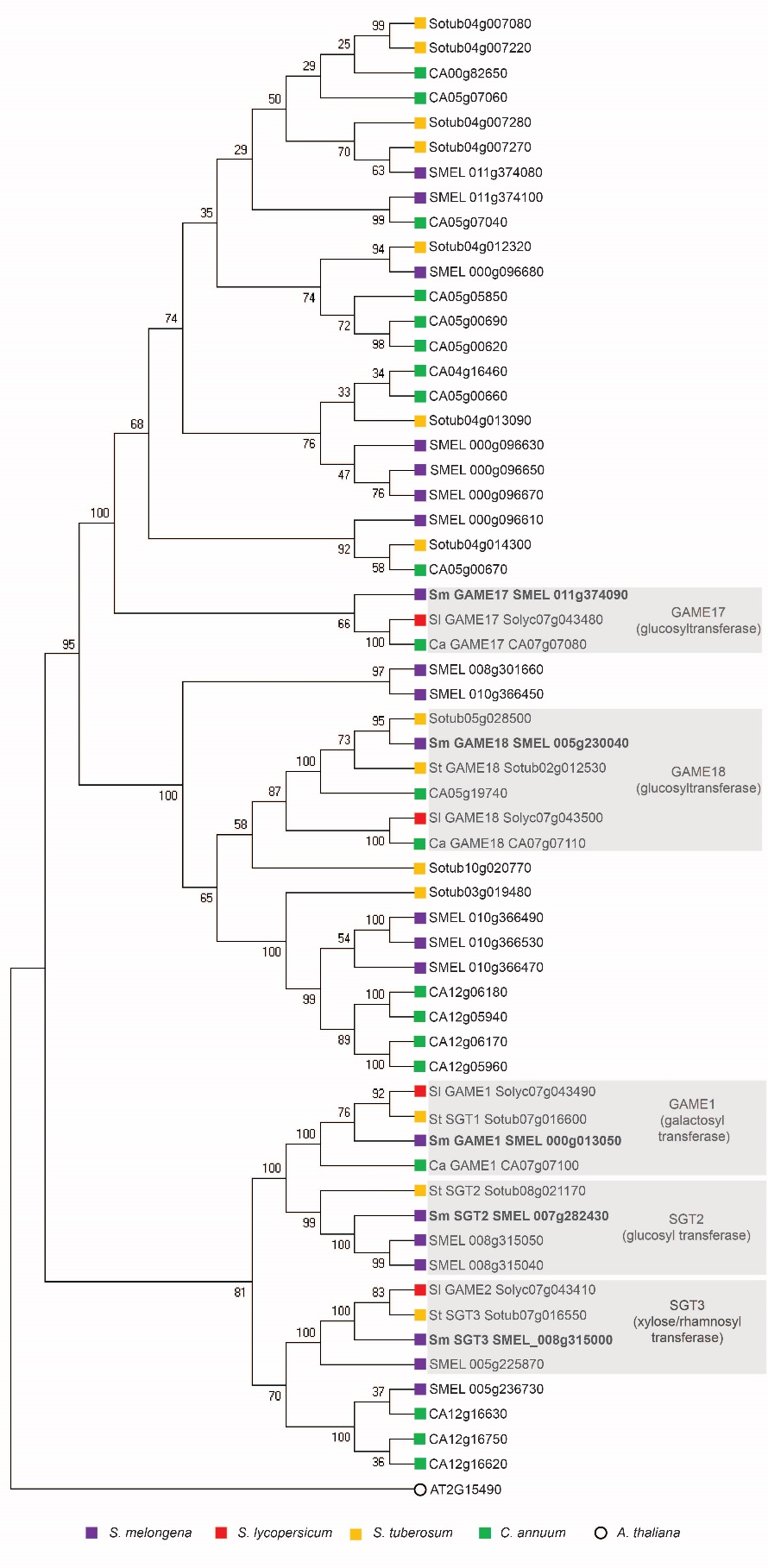
**Supplementary Figure S8. Phylogenetic relationships among plant glucosyltransferases participating in SGA biosynthesis**. Eggplant GTs likely to partake SGA biosynthesis are boxed. Protein sequences of known tomato glycosyltransferase genes involved in SGA biosynthesis^116^ were aligned against eggplant, pepper, potato and Arabidopsis protein databases using BLASTP. For eggplant, pepper and potato, BLAST hits with an e-value lower than 4E-138 were selected. Arabidopsis best hit was taken as an outgroup.

**
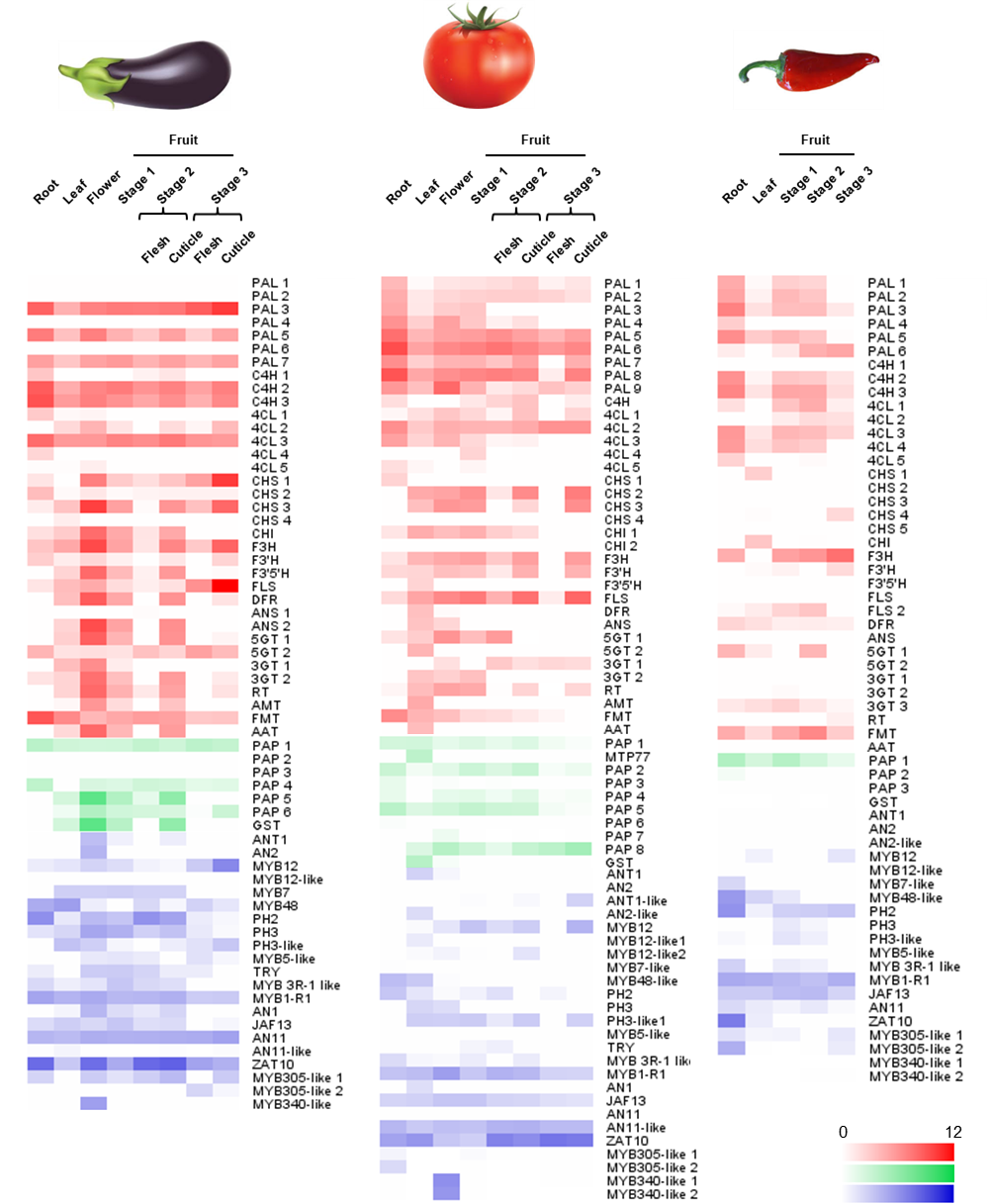
Supplementary Figure S9. Comparison of transcriptional profiles for anthocyanin biosynthesis, transport and regulatory candidate genes (depicted in red, green and blue, respectively).** Heat maps show log2-scaled reads per kilobase per million in eggplant and for its orthologs in tomato and pepper. Abbreviations are as in **Supplementary Table S34**.

**
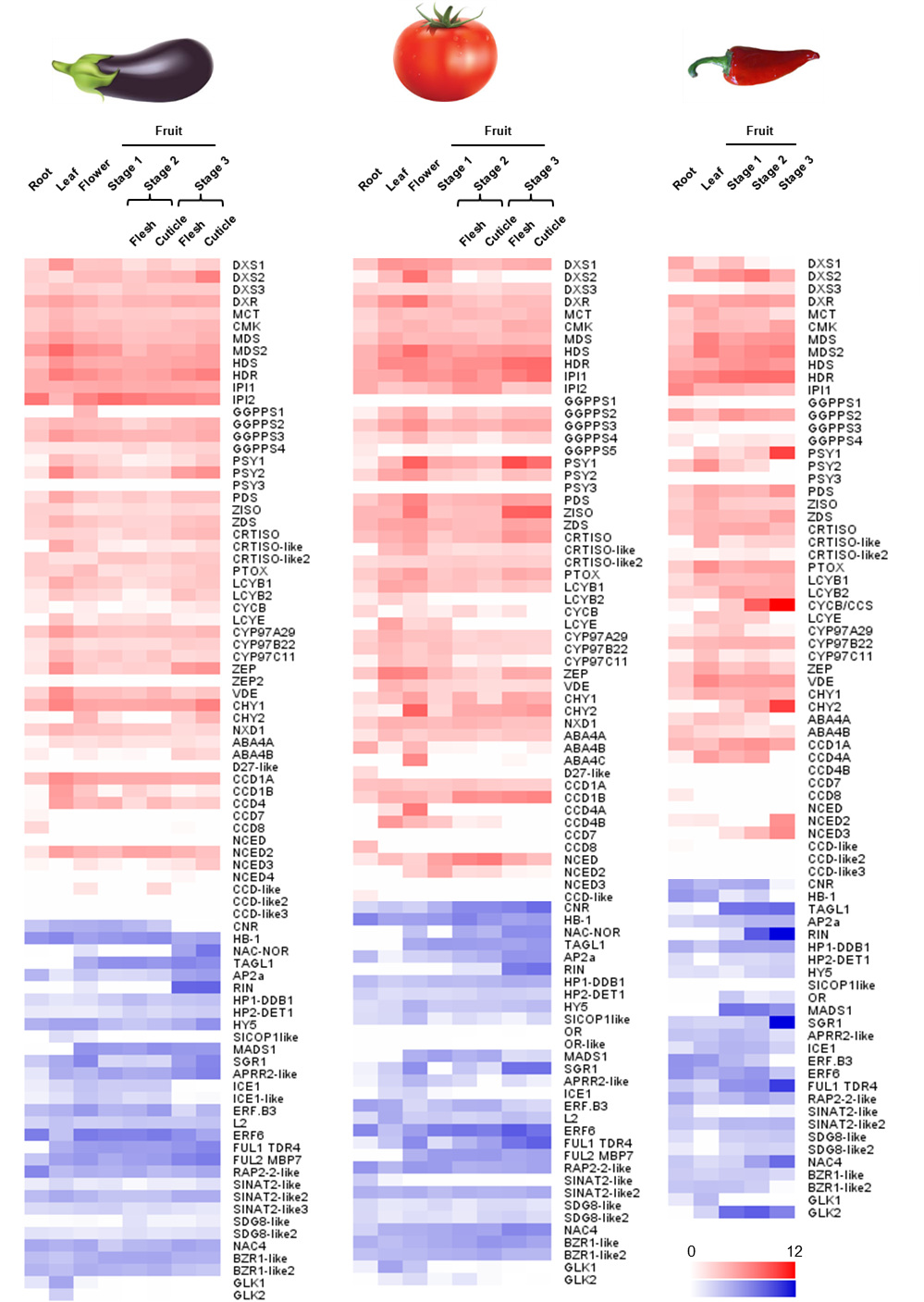
**

**Supplementary Figure S10.** **Comparison of transcriptional profiles of carotenoid biosynthetic and regulatory candidate genes depicted in red and blue, respectively.** Heat maps show log2-scaled reads per kilobase per million in eggplant and for its orthologs in tomato and pepper. Abbreviations are as in **Supplementary Table S35**.

# References

1. Carrier, G. et al. An efficient and rapid protocol for plant nuclear DNA preparation suitable for next generation sequencing methods. Am. J. Bot. 98, 15–17 (2011).
2. Portis, E. et al. QTL mapping in eggplant reveals clusters of yield-related loci and orthology with the tomato genome. PLoS One 9, e89499 (2014).
3. Toppino, L. et al. Mapping Quantitative Trait Loci Affecting Biochemical and Morphological Fruit Properties in Eggplant (Solanum melongena L.). Front. Plant Sci. 7, 256 (2016).
4. Barchi, L. et al. QTL analysis reveals new eggplant loci involved in resistance to fungal wilts. Euphytica 214, 20 (2018).
5. Elshire, R. J. et al. A robust, simple genotyping-by-sequencing (GBS) approach for high diversity species. PLoS One 6, 1–10 (2011).
6. Doyle, J. J. & Doyle, J. L. Isolation of plant DNA from fresh tissue. Focus (Madison). 12, 13–14 (1990).
7. Van Nieuwerburgh, F. et al. Illumina mate-paired DNA sequencing-library preparation using Cre-Lox recombination. Nucleic Acids Res. 40, e24 (2012).
8. Marçais, G. & Kingsford, C. A fast, lock-free approach for efficient parallel counting of occurrences of k-mers. Bioinformatics 27, 764–770 (2011).
9. Luo, R. et al. SOAPdenovo2: an empirically improved memory-efficient short-read de novo assembler. Gigascience 1, 18 (2012).
10. Simão, F. A., Waterhouse, R. M., Ioannidis, P., Kriventseva, E. V & Zdobnov, E. M. BUSCO: assessing genome assembly and annotation completeness with single-copy orthologs. *Bioinformatics* btv351- (2015). doi:10.1093/bioinformatics/btv351
11. Waterhouse, R. M., Tegenfeldt, F., Li, J., Zdobnov, E. M. & Kriventseva, E. V. OrthoDB: a hierarchical catalog of animal, fungal and bacterial orthologs. *Nucleic Acids Res.* **41,** D358-65 (2013).
12. VanBuren, R. et al. Single-molecule sequencing of the desiccation-tolerant grass Oropetium thomaeum. Nature 527, 508–511 (2015).
13. Cao, J. *et al.* Whole-genome sequencing of multiple *Arabidopsis thaliana* populations. *Nat. Genet.* **43,** 956–63 (2011).
14. Pendleton, M. *et al.* Assembly and diploid architecture of an individual human genome via single-molecule technologies. *Nat. Methods* **12,** 780–786 (2015).
15. Scaglione, D. *et al.* The genome sequence of the outbreeding globe artichoke constructed de novo incorporating a phase-aware low-pass sequencing strategy of F1 progeny. *Sci. Rep.* **6,** 19427 (2016).
16. Li, H. & Durbin, R. Fast and accurate short read alignment with Burrows–Wheeler transform. *Bioinformatics* **25,** 1754–1760 (2009).
17. Li, H. *et al.* The Sequence Alignment/Map format and SAMtools. *Bioinformatics* **25,** 2078–9 (2009).
18. Broman, K. W., Wu, H., Sen, S. & Churchill, G. A. R/qtl: QTL mapping in experimental crosses. *Bioinformatics* **19,** 889–90 (2003).
19. van Ooijen, J. W. JoinMap ® 4, Software for the calculation of genetic linkage maps in experimental populations. (2006).
20. Kosambi, D. D. The estimation of map distance from recombination values. **12(3),** 172–175 (1944).
21. van Os, H., Stam, P., Visser, R. G. F. & van Eck, H. J. SMOOTH: a statistical method for successful removal of genotyping errors from high-density genetic linkage data. *Theor. Appl. Genet.* **112,** 187–94 (2005).
22. Barchi, L. *et al.* Identification of SNP and SSR markers in eggplant using RAD tag sequencing. *BMC Genomics* **12,** (2011).
23. Kim, S. *et al.* Genome sequence of the hot pepper provides insights into the evolution of pungency in Capsicum species. *Nat. Genet.* **46,** 270–8 (2014).
24. Qin, C. *et al.* Whole-genome sequencing of cultivated and wild peppers provides insights into *Capsicum* domestication and specialization. *Proc. Natl. Acad. Sci.* **111,** 5135–5140 (2014).
25. The Tomato genome Consortium. The tomato genome sequence provides insights into fleshy fruit evolution. *Nature* **485,** 635–41 (2012).
26. The Potato Genome Sequencing Consortiu*.* Genome sequence and analysis of the tuber crop potato. *Nature* **475,** 189–195 (2011).
27. Simpson, J. T. *et al.* ABySS: a parallel assembler for short read sequence data. *Genome Res.* **19,** 1117–23 (2009).
28. Kent, W. J. BLAT--the BLAST-like alignment tool. *Genome Res.* **12,** 656–64 (2002).
29. Smit, AFA, Hubley, R & Green, P. RepeatMasker Open-4.0.
30. Kent, W. J., Baertsch, R., Hinrichs, A., Miller, W. & Haussler, D. Evolution’s cauldron: duplication, deletion, and rearrangement in the mouse and human genomes. *Proc. Natl. Acad. Sci. U. S. A.* **100,** 11484–9 (2003).
31. UCSC. UCSC reciprocal. Available at: http://genomewiki.ucsc.edu/index.php/HowTo:_Syntenic_Net_or_Reciprocal_Best.
32. Mennella, G. *et al.* Characterization of health-related compounds in eggplant (Solanum melongena L.) lines derived from introgression of allied species. *J. Agric. Food Chem.* **58,** 7597–603 (2010).
33. The Tomato genome Consortium. The tomato genome sequence provides insights into fleshy fruit evolution. *Nature* **485,** 635–41 (2012).
34. Bolger, A. M., Lohse, M. & Usadel, B. Trimmomatic: A flexible trimmer for Illumina sequence data. *Bioinformatics* **30,** 2114–2120 (2014).
35. Kim, D. *et al.* TopHat2: accurate alignment of transcriptomes in the presence of insertions, deletions and gene fusions. *Genome Biol.* **14,** R36 (2013).
36. Trapnell, C. *et al.* Transcript assembly and quantification by RNA-Seq reveals unannotated transcripts and isoform switching during cell differentiation. *Nat. Biotechnol.* **28,** 511–5 (2010).
37. Schulz, M. H., Zerbino, D. R., Vingron, M. & Birney, E. Oases: Robust de novo RNA-seq assembly across the dynamic range of expression levels. *Bioinformatics* **28,** 1086–1092 (2012).
38. Campbell, M. S. *et al.* MAKER-P: a tool kit for the rapid creation, management, and quality control of plant genome annotations. *Plant Physiol.* **164,** 513–24 (2014).
39. Smit, AFA, Hubley, R. RepeatModeler Open-1.0. 2008–2015
40. Kohany, O., Gentles, A. J., Hankus, L. & Jurka, J. Annotation, submission and screening of repetitive elements in Repbase: RepbaseSubmitter and Censor. *BMC Bioinformatics* **7,** 474 (2006).
41. Shearer, L. A. *et al.* Fluorescence in situ hybridization and optical mapping to correct scaffold arrangement in the tomato genome. *G3 (Bethesda).* **4,** 1395–405 (2014).
42. Staton, S. E. *et al.* The sunflower (*Helianthus annuus* L.) genome reflects a recent history of biased accumulation of transposable elements. *Plant J.* **72,** 142–53 (2012).
43. Ellinghaus, D., Kurtz, S. & Willhoeft, U. LTRharvest, an efficient and flexible software for de novo detection of LTR retrotransposons. *BMC Bioinformatics* **9,** 18 (2008).
44. Steinbiss, S., Willhoeft, U., Gremme, G. & Kurtz, S. Fine-grained annotation and classification of de novo predicted LTR retrotransposons. *Nucleic Acids Res.* **37,** 7002–13 (2009).
45. Punta, M. *et al.* The Pfam protein families database. *Nucleic Acids Res.* **40,** D290-301 (2012).
46. hmmer. Available at: http://hmmer.janelia.org/.
47. Kurtz, S. The Vmatch large scale sequence analysis software - a manual. (2010).
48. Wicker, T. *et al.* A unified classification system for eukaryotic transposable elements. *Nat. Rev. Genet.* **8,** 973–82 (2007).
49. Wicker, T., Matthews, D. E. & Keller, B. TREP: a database for Triticeae repetitive elements. *Trends Plant Sci.* **7,** 561–562 (2002).
50. Vitte, C., Panaud, O. & Quesneville, H. LTR retrotransposons in rice (*Oryza sativa*, L.): recent burst amplifications followed by rapid DNA loss. *BMC Genomics* **8,** 218 (2007).
51. Baucom, R. S., Estill, J. C., Leebens-Mack, J. & Bennetzen, J. L. Natural selection on gene function drives the evolution of LTR retrotransposon families in the rice genome. *Genome Res.* **19,** 243–54 (2009).
52. Sievers, F. *et al.* Fast, scalable generation of high-quality protein multiple sequence alignments using Clustal Omega. *Mol. Syst. Biol.* **7,** 539 (2011).
53. Yang, Z. PAML 4: phylogenetic analysis by maximum likelihood. *Mol. Biol. Evol.* **24,** 1586–91 (2007).
54. Strasburg, J. L. & Rieseberg, L. H. Molecular demographic history of the annual sunflowers *Helianthus annuus* and *H. petiolaris*--large effective population sizes and rates of long-term gene flow. *Evolution* **62,** 1936–50 (2008).
55. Giordani, T., Cavallini, A. & Natali, L. The repetitive component of the sunflower genome. *Current Plant Biology* **1,** 45–54 (2014).
56. Li, F. *et al.* Genome sequence of the cultivated cotton *Gossypium* *arboreum*. *Nat. Genet.* **46,** 567–572 (2014).
57. Xu, Q. *et al.* The draft genome of sweet orange (*Citrus* *sinensis*). *Nat. Genet.* **45,** 59–66 (2012).
58. Schmutz, J. *et al.* A reference genome for common bean and genome-wide analysis of dual domestications. *Nat. Genet.* **46,** 707–713 (2014).
59. Project, I. R. G. S. & Sasaki, T. The map-based sequence of the rice genome. *Nature* **436,** 793–800 (2005).
60. D’Hont, A. *et al.* The banana (*Musa* *acuminata*) genome and the evolution of monocotyledonous plants. *Nature* **488,** 213–217 (2012).
61. Guigó, R., Knudsen, S., Drake, N. & Smith, T. Prediction of gene structure. *J. Mol. Biol.* **226,** 141–157 (1992).
62. Parra, G., Blanco, E. & Guigó, R. GeneID in Drosophila. *Genome Res.* **10,** 511–5 (2000).
63. Stanke, M. *et al.* AUGUSTUS: ab initio prediction of alternative transcripts. *Nucleic Acids Res.* **34,** W435-9 (2006).
64. Korf, I., Flicek, P., Duan, D. & Brent, M. R. Integrating genomic homology into gene structure prediction. *Bioinformatics* **17 Suppl 1,** S140-8 (2001).
65. Trapnell, C. *et al.* Differential analysis of gene regulation at transcript resolution with RNA-seq. *Nat. Biotechnol.* **31,** 46–53 (2012).
66. The UniProt Consortium. UniProt: a hub for protein information. *Nucleic Acids Res.* **43,** D204-212 (2014).
67. Jones, P. *et al.* InterProScan 5: genome-scale protein function classification. *Bioinformatics* **30,** 1236–40 (2014).
68. Sigrist, C. J. A. *et al.* New and continuing developments at PROSITE. *Nucleic Acids Res.* **41,** D344-7 (2013).
69. Attwood, T. K. *et al.* The PRINTS database: a fine-grained protein sequence annotation and analysis resource--its status in 2012. *Database (Oxford).* **2012,** bas019 (2012).
70. Bru, C. *et al.* The ProDom database of protein domain families: more emphasis on 3D. *Nucleic Acids Res.* **33,** D212-5 (2005).
71. Letunic, I., Doerks, T. & Bork, P. SMART 7: recent updates to the protein domain annotation resource. *Nucleic Acids Res.* **40,** D302-5 (2012).
72. Haft, D. H. *et al.* TIGRFAMs and Genome Properties in 2013. *Nucleic Acids Res.* **41,** D387-95 (2013).
73. Wu, C. H. *et al.* PIRSF: family classification system at the Protein Information Resource. *Nucleic Acids Res.* **32,** D112-4 (2004).
74. Gough, J., Karplus, K., Hughey, R. & Chothia, C. Assignment of homology to genome sequences using a library of hidden Markov models that represent all proteins of known structure. *J. Mol. Biol.* **313,** 903–19 (2001).
75. de Lima Morais, D. A. *et al.* SUPERFAMILY 1.75 including a domain-centric gene ontology method. *Nucleic Acids Res.* **39,** D427-34 (2011).
76. Lees, J. *et al.* Gene3D: a domain-based resource for comparative genomics, functional annotation and protein network analysis. *Nucleic Acids Res.* **40,** D465-71 (2012).
77. Mi, H., Muruganujan, A. & Thomas, P. D. PANTHER in 2013: modeling the evolution of gene function, and other gene attributes, in the context of phylogenetic trees. *Nucleic Acids Res.* **41,** D377-86 (2013).
78. The Gene Ontology Consortium. Gene Ontology Consortium: going forward. *Nucleic Acids Res.* **43,** D1049-1056 (2014).
79. Hirakawa, H. *et al.* Draft genome sequence of eggplant (*Solanum* *melongena* L.): the representative solanum species indigenous to the old world. *DNA Res.* **21,** 649–60 (2014).
80. Lamesch, P. *et al.* The Arabidopsis Information Resource (TAIR): improved gene annotation and new tools. *Nucleic Acids Res.* **40,** D1202-10 (2012).
81. Li, L., Stoeckert, C. J. & Roos, D. S. OrthoMCL: identification of ortholog groups for eukaryotic genomes. *Genome Res.* **13,** 2178–89 (2003).
82. Altschul, S. F., Gish, W., Miller, W., Myers, E. W. & Lipman, D. J. Basic local alignment search tool. *J. Mol. Biol.* **215,** 403–10 (1990).
83. Mathelier, A. & Carbone, A. MIReNA: finding microRNAs with high accuracy and no learning at genome scale and from deep sequencing data. *Bioinformatics* **26,** 2226–34 (2010).
84. Kozomara, A. & Griffiths-Jones, S. miRBase: integrating microRNA annotation and deep-sequencing data. *Nucleic Acids Res.* **39,** D152-7 (2011).
85. Chen, W. *et al.* Tuning LeSPL-CNR expression by SlymiR157 affects tomato fruit ripening. *Sci. Rep.* **5,** 7852 (2015).
86. Cuperus, J. T., Fahlgren, N. & Carrington, J. C. Evolution and functional diversification of MIRNA genes. *Plant Cell* **23,** 431–42 (2011).
87. Chávez Montes, R. A. *et al.* Sample sequencing of vascular plants demonstrates widespread conservation and divergence of microRNAs. *Nat. Commun.* **5,** 3722 (2014).
88. Bonnet, E., He, Y., Billiau, K. & Van de Peer, Y. TAPIR, a web server for the prediction of plant microRNA targets, including target mimics. *Bioinformatics* **26,** 1566–8 (2010).
89. Du, Z., Zhou, X., Ling, Y., Zhang, Z. & Su, Z. agriGO: a GO analysis toolkit for the agricultural community. *Nucleic Acids Res.* **38,** W64-70 (2010).
90. Cingolani, P. *et al.* A program for annotating and predicting the effects of single nucleotide polymorphisms, SnpEff: SNPs in the genome of *Drosophila* *melanogaster* strain w1118; iso-2; iso-3. *Fly (Austin).* **6,** 80–92 (2012).
91. Lyons, E., Pedersen, B., Kane, J. & Freeling, M. The value of nonmodel genomes and an example using synmap within CoGe to dissect the hexaploidy that predates the Rosids. *Trop. Plant Biol.* **1,** 181–190 (2008).
92. Haas, B. J., Delcher, A. L., Wortman, J. R. & Salzberg, S. L. DAGchainer: a tool for mining segmental genome duplications and synteny. *Bioinformatics* **20,** 3643–6 (2004).
93. Tang, H. *et al.* Screening synteny blocks in pairwise genome comparisons through integer programming. *BMC Bioinformatics* **12,** 102 (2011).
94. Lynch, M. & Conery, J. S. The evolutionary fate and consequences of duplicate genes. *Science* **290,** 1151–5 (2000).
95. Wikström, N., Savolainen, V. & Chase, M. W. Evolution of the angiosperms: calibrating the family tree. *Proc. Biol. Sci.* **268,** 2211–20 (2001).
96. Abrouk, M. *et al.* Palaeogenomics of plants: synteny-based modelling of extinct ancestors. *Trends Plant Sci.* **15,** 479–87 (2010).
97. Wang, D. *et al.* How do variable substitution rates influence Ka and Ks calculations? *Genomics. Proteomics Bioinformatics* **7,** 116–27 (2009).
98. Wang, X., Shi, X., Hao, B., Ge, S. & Luo, J. Duplication and DNA segmental loss in the rice genome: implications for diploidization. *New Phytol.* **165,** 937–46 (2005).
99. Schnable, J. C., Freeling, M. & Lyons, E. Genome-wide analysis of syntenic gene deletion in the grasses. *Genome Biol. Evol.* **4,** 265–77 (2012).
100. Sankoff, D., Zheng, C. & Zhu, Q. The collapse of gene complement following whole genome duplication. *BMC Genomics* **11,** 313 (2010).
101. Abrouk, M. *et al.* Grass microRNA gene paleohistory unveils new insights into gene dosage balance in subgenome partitioning after whole-genome duplication. *Plant Cell* **24,** 1776–92 (2012).
102. Thomas, B. C., Pedersen, B. & Freeling, M. Following tetraploidy in an Arabidopsis ancestor, genes were removed preferentially from one homeolog leaving clusters enriched in dose-sensitive genes. *Genome Res.* **16,** 934–46 (2006).
103. Pont, C., Murat, F., Confolent, C., Balzergue, S. & Salse, J. RNA-seq in grain unveils fate of neo- and paleopolyploidization events in bread wheat (*Triticum* *aestivum* L.). *Genome Biol.* **12,** R119 (2011).
104. Wu, F. & Tanksley, S. D. Chromosomal evolution in the plant family Solanaceae. *BMC Genomics* **11,** 182 (2010).
105. Salse, J. In silico archeogenomics unveils modern plant genome organisation, regulation and evolution. *Curr. Opin. Plant Biol.* **15,** 122–30 (2012).
106. Pevzner, P. & Tesler, G. Genome rearrangements in mammalian evolution: lessons from human and mouse genomes. *Genome Res.* **13,** 37–45 (2003).
107. Alekseyev, M. A. & Pevzner, P. A. Breakpoint graphs and ancestral genome reconstructions. *Genome Res.* **19,** 943–57 (2009).
108. Perrin, A., Varré, J.-S., Blanquart, S. & Ouangraoua, A. ProCARs: Progressive Reconstruction of Ancestral Gene Orders. *BMC Genomics* **16 Suppl 5,** S6 (2015).
109. Jones, D. T., Taylor, W. R. & Thornton, J. M. The rapid generation of mutation data matrices from protein sequences. Comput. Appl. Biosci. 8, 275–82 (1992).
110. Katoh, K. & Standley, D. M. MAFFT multiple sequence alignment software version 7: improvements in performance and usability. Mol. Biol. Evol. 30, 772–80 (2013).
111. Tamura, K., Stecher, G., Peterson, D., Filipski, A. & Kumar, S. MEGA6: Molecular Evolutionary Genetics Analysis Version 6.0. Mol. Biol. Evol. 30, 2725–2729 (2013).
112. Andolfo, G. et al. Overview of tomato (Solanum lycopersicum) candidate pathogen recognition genes reveals important Solanum R locus dynamics. New Phytol. 197, 223–37 (2013).
113. Andolfo, G. et al. Defining the full tomato NB-LRR resistance gene repertoire using genomic and cDNA RenSeq. BMC Plant Biol. 14, 120 (2014).
114. Aversano, R. et al. The Solanum commersonii genome sequence provides insights into adaptation to stress conditions and genome evolution of wild potato relatives. Plant Cell 27, 954–68 (2015).
115. González, C. et al. Stress-response balance drives the evolution of a network module and its host genome. Mol. Syst. Biol. 11, 827 (2015).
116. Hayashi, K. & Yoshida, H. Refunctionalization of the ancient rice blast disease resistance gene Pit by the recruitment of a retrotransposon as a promoter. Plant J. 57, 413–25 (2009).
117. Eich, E. Solanaceae and Convolvulaceae: Secondary Metabolites: Biosynthesis, Chemotaxonomy, Biological and Economic Significance (A Handbook). (Springer Berlin Heidelberg, 2008).
118. Cárdenas, P. D. et al. The bitter side of the nightshades: Genomics drives discovery in Solanaceae steroidal alkaloid metabolism. Phytochemistry 113, 24–32 (2015).
119. Friedman, M. Chemistry and Anticarcinogenic Mechanisms of Glycoalkaloids Produced by Eggplants, Potatoes, and Tomatoes. J. Agric. Food Chem. 63, 3323–3337 (2015).
120. Itkin, M. et al. Biosynthesis of antinutritional alkaloids in solanaceous crops is mediated by clustered genes. Science 341, 175–9 (2013).
121. Manning, K. et al. A naturally occurring epigenetic mutation in a gene encoding an SBP-box transcription factor inhibits tomato fruit ripening. Nat. Genet. 38, 948–952 (2006).
122. Seymour, G. B., Østergaard, L., Chapman, N. H., Knapp, S. & Martin, C. Fruit Development and Ripening. Annu. Rev. Plant Biol. 64, 219–241 (2013).
123. Vrebalov, J. et al. Fleshy fruit expansion and ripening are regulated by the Tomato SHATTERPROOF gene TAGL1. Plant Cell 21, 3041–62 (2009).
124. Tzfadia, O. et al. CoExpNetViz: Comparative Co-Expression Networks Construction and Visualization Tool. Front. Plant Sci. 6, 1194 (2016).
125. Dong, T. et al. A non-climacteric fruit gene CaMADS-RIN regulates fruit ripening and ethylene biosynthesis in climacteric fruit. PLoS One 9, e95559 (2014).
126. Povero, G., Gonzali, S., Bassolino, L., Mazzucato, A. & Perata, P. Transcriptional analysis in high-anthocyanin tomatoes reveals synergistic effect of Aft and atv genes. J. Plant Physiol. 168, 270–279 (2011).
127. Stommel, J. R. & Dumm, J. M. Coordinated regulation of biosynthetic and regulatory genes coincides with anthocyanin accumulation in developing eggplant fruit. J. Amer. Soc. Hort. Sci. 140, 129–135 (2015).
128. Luo, P. et al. Disequilibrium of Flavonol Synthase and Dihydroflavonol-4-Reductase expression associated tightly to white vs. red color flower formation in plants. Front. Plant Sci. 6, 1257 (2015).
129. Yuan, Y.-W., Rebocho, A. B., Sagawa, J. M., Stanley, L. E. & Bradshaw, H. D. Competition between anthocyanin and flavonol biosynthesis produces spatial pattern variation of floral pigments between Mimulus species. Proc. Natl. Acad. Sci. U. S. A. 113, 2448–53 (2016).
130. Zhang, Y. et al. Multi-level engineering facilitates the production of phenylpropanoid compounds in tomato. Nat. Commun. 6, 8635 (2015).
131. Luo, J. et al. AtMYB12 regulates caffeoyl quinic acid and flavonol synthesis in tomato: expression in fruit results in very high levels of both types of polyphenol. Plant J. 56, 316–26 (2008).
132. Petroni, K. & Tonelli, C. Recent advances on the regulation of anthocyanin synthesis in reproductive organs. Plant Sci. 181, 219–229 (2011).
133. Kiferle, C. et al. Tomato R2R3-MYB Proteins SlANT1 and SlAN2: same protein activity, different roles. PLoS One 10, e0136365 (2015).
134. Docimo, T. et al. Phenylpropanoids accumulation in eggplant fruit: characterization of biosynthetic genes and regulation by a MYB transcription factor. Front. Plant Sci. 6, 1233 (2015).
135. van Nocker, S. & Ludwig, P. The WD-repeat protein superfamily in Arabidopsis: conservation and divergence in structure and function. BMC Genomics 4, 50 (2003).
136. EL-Qudah, J. M. & others. Identification and quantification of major carotenoids in some vegetables. Am. J. Appl. Sci. 6, 492 (2009).
137. Giuliano, G. Plant carotenoids: genomics meets multi-gene engineering. Curr. Opin. Plant Biol. 19, 111–117 (2014).
138. Ohmiya, A., Kishimoto, S., Aida, R., Yoshioka, S. & Sumitomo, K. Carotenoid cleavage dioxygenase (CmCCD4a) contributes to white color formation in chrysanthemum petals. Plant Physiol. 142, 1193–201 (2006).
139. Brandi, F. et al. Study of ‘Redhaven’ peach and its white-fleshed mutant suggests a key role of CCD4 carotenoid dioxygenase in carotenoid and norisoprenoid volatile metabolism. BMC Plant Biol. 11, 24 (2011).
140. Barry, C. S., McQuinn, R. P., Chung, M.-Y., Besuden, A. & Giovannoni, J. J. Amino acid substitutions in homologs of the STAY-GREEN protein are responsible for the green-flesh and chlorophyll retainer mutations of tomato and pepper. Plant Physiol. 147, 179–87 (2008).
141. Pan, Y. et al. Network inference analysis identifies an APRR2-like gene linked to pigment accumulation in tomato and pepper fruits. Plant Physiol. 161, 1476–85 (2013).
142. Pighin, J. A. et al. Plant cuticular lipid export requires an ABC transporter. Science 306, 702–4 (2004).
143. Pollard, M., Beisson, F., Li, Y. & Ohlrogge, J. B. Building lipid barriers: biosynthesis of cutin and suberin. Trends Plant Sci. 13, 236–46 (2008).
144. Bolger, A. et al. The genome of the stress-tolerant wild tomato species Solanum pennellii. Nat. Genet. 46, 1034–8 (2014).
145. Kunst, L. & Samuels, A. L. Biosynthesis and secretion of plant cuticular wax. Prog. Lipid Res. 42, 51–80 (2003).
146. Leide, J., Hildebrandt, U., Reussing, K., Riederer, M. & Vogg, G. The developmental pattern of tomato fruit wax accumulation and its impact on cuticular transpiration barrier properties: effects of a deficiency in a beta-ketoacyl-coenzyme A synthase (LeCER6). Plant Physiol. 144, 1667–79 (2007).
147. Yeats, T. H. et al. The identification of cutin synthase: formation of the plant polyester cutin. Nat. Chem. Biol. 8, 609–11 (2012).
148. Panikashvili, D. et al. The Arabidopsis DESPERADO/AtWBC11 transporter is required for cutin and wax secretion. Plant Physiol. 145, 1345–60 (2007).
149. Panikashvili, D. & Aharoni, A. ABC-type transporters and cuticle assembly: Linking function to polarity in epidermis cells. Plant Signal. Behav. 3, 806–9 (2008).
150. Bessire, M. et al. A member of the PLEIOTROPIC DRUG RESISTANCE family of ATP binding cassette transporters is required for the formation of a functional cuticle in Arabidopsis. Plant Cell 23, 1958–70 (2011).
151. Lara, I., Belge, B. & Goulao, L. F. A focus on the biosynthesis and composition of cuticle in fruits. J. Agric. Food Chem. 63, 4005–19 (2015).
152. Aharoni, A. et al. The SHINE clade of AP2 domain transcription factors activates wax biosynthesis, alters cuticle properties, and confers drought tolerance when overexpressed in Arabidopsis. Plant Cell 16, 2463–80 (2004).
153. Shi, J. X. et al. SHINE transcription factors act redundantly to pattern the archetypal surface of Arabidopsis flower organs. PLoS Genet. 7, e1001388 (2011).
154. Chevalier, C. et al. Endoreduplication and fruit growth in tomato: evidence in favour of the karyoplasmic ratio theory. J. Exp. Bot. 65, 2731–2746 (2014).
